# Conserved features of cohesin binding along fission yeast chromosomes

**DOI:** 10.1186/gb-2009-10-5-r52

**Published:** 2009-05-19

**Authors:** Christine K Schmidt, Neil Brookes, Frank Uhlmann

**Affiliations:** 1 Chromosome Segregation Laboratory, Cancer Research UK London Research Institute, Lincoln's Inn Fields, London WC2A 3PX, UK; 2 Bioinformatics and Biostatistics Service, Cancer Research UK London Research Institute, Lincoln's Inn Fields, London WC2A 3PX, UK; 3 Current address: National Cancer Institute, NIH, Bethesda, MD 20892, USA; 4 Current address: Trinity Centre for High Performance Computing, Trinity College, Dublin 2, Ireland

## Abstract

**Background:**

Cohesin holds sister chromatids together to enable their accurate segregation in mitosis. How, and where, cohesin binds to chromosomes are still poorly understood, and recent genome-wide surveys have revealed an apparent disparity between its chromosomal association patterns in different organisms.

**Results:**

Here, we present the high-resolution analysis of cohesin localization along fission yeast chromosomes. This reveals that several determinants, thought specific for different organisms, come together to shape the overall distribution. Cohesin is detected at chromosomal loading sites, characterized by the cohesin loader Mis4/Ssl3, in regions of strong transcriptional activity. Cohesin also responds to transcription by downstream translocation and accumulation at convergent transcriptional terminators surrounding the loading sites. As cells enter mitosis, a fraction of cohesin leaves chromosomes in a cleavage-independent reaction, while a substantial pool of cohesin dissociates when it is cleaved at anaphase onset. We furthermore observe that centromeric cohesin spreads out onto chromosome arms during mitosis, dependent on Aurora B kinase activity, emphasizing the plasticity of cohesin behavior.

**Conclusions:**

Our findings suggest that features that were thought to differentiate cohesin between organisms collectively define the overall behavior of fission yeast cohesin. Apparent differences between organisms might reflect an emphasis on different aspects, rather than different principles, of cohesin action.

## Abstract 2

High-resolution analysis of cohesin localization on fission yeast chromosomes reveals that several determinants, previously thought to be organism-specific, come together to shape overall distribution.

## Background

After DNA replication in S phase, sister chromatids are held together by the cohesin complex. This allows DNA break repair by homologous recombination in G2 and bipolar attachment of the spindle to sister kinetochores in mitosis. At anaphase onset, sister chromatid cohesion is resolved to trigger chromosome segregation (reviewed in [1,2]). Cohesin is an essential, conserved protein complex consisting of at least four subunits, Psm1, Psm3, Rad21 and Psc3 in fission yeast, as well as a less firmly associated fifth subunit, Pds5 (orthologs of budding yeast Smc1, Smc3, Scc1, Scc3 and Pds5, respectively) [3,4]. Biochemical studies and electron micrographs have shown that cohesin forms large proteinaceous rings. Together with strong experimental evidence, this has fostered the idea that cohesin binds to and holds sister chromatids together by topological embrace [5].

Several studies have investigated cohesin's chromosomal binding sites in different model organisms. Despite its conserved function in DNA repair and mitosis, no common rule has emerged that defines these sites. In budding yeast, cohesin appears to be excluded from transcribed open reading frames (ORFs) and accumulates almost exclusively at convergent RNA polymerase II (Pol II) transcriptional termination sites (called 'convergent sites' in the following) [6-8]. In contrast, in mammalian cells cohesin colocalizes along chromosomes with CTCF, a DNA-binding zinc-finger protein required for transcriptional insulation, with no strong preference with respect to ORF location or orientation [9,10]. The functional interaction with CTCF has highlighted an additional conserved, but poorly understood, role of cohesin in transcriptional regulation [11-13]. Although CTCF is conserved in the fruit fly, cohesin exhibits yet a different binding pattern in this organism, associating with highly transcribed genes throughout the non-repetitive genome [13,14].

A preliminary analysis in fission yeast found cohesin enriched at convergent sites [7], although closer examination of the pattern, which we report here, reveals further determinants of binding. In addition, the fission yeast heterochromatin protein 1 (HP1) ortholog, Swi6, acts to enrich cohesin at centromeres and telomeres [15,16]. Swi6 interacts with cohesin, and it has been suggested that it is also involved in cohesin recruitment to convergent sites along chromosome arms [17]. A possible contribution of heterochromatin to human centromeric cohesin enrichment has remained controversial [18,19]. In particular, it is unclear how heterochromatin could maintain centromeric cohesin during mitosis, when HP1 dissociates from chromatin after aurora B kinase-dependent phosphorylation of histone H3 [20,21].

Little is known about the Mis4/Ssl3 cohesin loader (orthologs of budding yeast Scc2/Scc4), a protein complex that is required for cohesin's association with chromosomes [3,22,23]. How the cohesin loader recognizes its binding sites on chromosomes, and how it promotes cohesin loading at these sites, are poorly understood. In budding yeast, Scc2/Scc4 binding correlates with high transcriptional activity along chromosome 6 [7], and a recent genome-wide survey found tRNA genes, other RNA polymerase III (Pol III) transcribed genes, and Pol II-transcribed genes encoding ribosomal protein components among its binding sites [24]. While budding yeast cohesin appears to translocate away from these loading sites to accumulate at convergent sites [7], *Drosophila *cohesin has been found to largely colocalize with the Scc2 ortholog Nipped-B [14]. In *Xenopus *oocyte extracts, binding of the Scc2/Scc4 cohesin loader to transcriptionally silent chromosomes depends on the pre-replicative complex involved in initiation of DNA replication [25,26]. The localization of Scc2/Scc4 in transcriptionally active somatic cells has not yet been studied. Mutations in human Scc2 are the cause of Cornelia de Lange syndrome, a severe developmental disorder, which has been taken to suggest a contribution of the Scc2/Scc4 complex, in conjunction with cohesin, to transcriptional regulation [27].

Chromosome segregation at anaphase onset is triggered when the protease separase is activated to cleave cohesin's Rad21 subunit [3,28,29]. In higher eukaryotes, but not in budding yeast, a significant fraction of cohesin is already released from chromosome arms as chromosomes condense in prophase. This prophase pathway of cohesin removal is independent of separase, but depends on cohesin phosphorylation by Polo-like kinase and on the cohesin destabilizer Wapl [29,30]. The regulation of mitotic cohesin removal in fission yeast remains poorly characterized. Only a small fraction of cohesin is thought to be cleaved at anaphase onset, but whether and where cohesin is removed from chromosome arms during prophase is not known. A cytological study found that cohesin remains chromosome-bound throughout mitosis [3].

Here we have analyzed the localization of fission yeast cohesin and its loader throughout the mitotic cell cycle using chromatin immunoprecipitation followed by hybridization to high density oligonucleotide tiling arrays. We find that cohesin is enriched at convergent sites but, unlike in budding yeast, only approximately half of all convergent sites are bound by cohesin. In addition, we find cohesin away from convergent sites, coinciding with its Mis4/Ssl3 loading complex at tRNA genes, ribosomal protein genes and additional strongly transcribed genes. As cells enter mitosis, a small fraction of cohesin is released from chromosomes in a cleavage-independent reaction, while a substantial fraction dissociates from chromosomes as it is cleaved at anaphase onset. Detectable amounts of cohesin remain associated with chromosomes during anaphase, suggesting that not all of cohesin participates in sister chromatid cohesion. We find that as centromeric Swi6 dissociates from chromosomes in mitosis, cohesin spreads from centromeres onto neighboring sequences. Our findings suggest that features that were thought to differentiate cohesin behavior between organisms collectively define the overall behavior of fission yeast cohesin. Apparent differences between organisms could reflect an emphasis on different aspects, rather than different principles, of cohesin behavior.

## Results

### Cohesin binding to an ordered subset of convergent sites along chromosome arms

**Figure 1:**
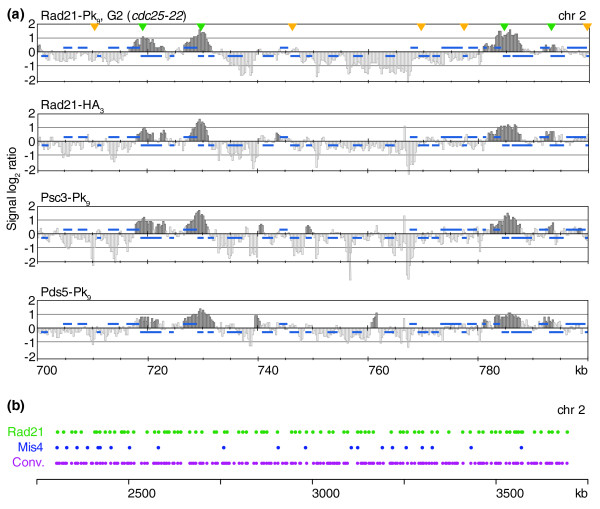
Cohesin binding to a subset of convergent sites along fission yeast chromosome arms. **(a) **Comparison of the binding patterns of the cohesin subunits Rad21-Pk_9 _in G2-arrested cells, and Rad21-HA_3_, Psc3-Pk_9 _and Pds5-Pk_9 _in exponentially growing cells. Enrichment of DNA fragments in the immunoprecipitate relative to a whole genome DNA sample is shown along a 100 kb region of fission yeast chromosome 2. Each bar represents the average of 11 oligonucleotide probes within adjacent 250 bp windows. The y-axis scale is log_2_. Dark grey signals represent significant binding as described [53]. Blue bars above and below the midline represent ORFs transcribed from left to right and opposite, respectively. Convergent sites that are bound, or not bound, by cohesin are marked with green and yellow arrowheads, respectively. **(b) **The location of Rad21-Pk_9 _peaks (green), assigned as described in Figure S1 in Additional data file 1, along 1.5 Mb of chromosome 2 is compared to the binding sites of the cohesin loader Mis4/Ssl3 (blue; compare Figure 3) and the distribution of convergent sites (Conv., purple).

To analyze the binding pattern of cohesin along fission yeast chromosomes, we hybridized cohesin chromatin immunoprecipitates to oligonucleotide tiling arrays covering two of the three fission yeast chromosomes (chromosomes 2 and 3). ChIP against three different components of cohesin, fused to two different epitope tags (Rad21-Pk_9_, Rad21-HA_3_, Psc3-Pk_9 _and Pds5-Pk_9_), yielded a reproducible binding pattern. The pattern was indistinguishable between exponentially growing cell populations and cells arrested in G2 using the thermosensitive *cdc25-22 *mutation (Figure 1a). Using a peak picking algorithm, we identified 228 binding sites along chromosome 2 (Figure S1 in Additional data file 1). With a length of 4.6 Mb, chromosome 2 represents about one-third of the fission yeast genome. Of the peaks, 214 (94%) overlapped with convergent sites along the chromosome arms. However, unlike in budding yeast where cohesin is found at almost every convergent site [6-8], only 52% of all convergent sites were bound by cohesin in fission yeast (Figure 1b). In addition, there were 14 assigned cohesin peaks away from convergent sites (see below).

Sister chromatid cohesion along chromosome arms facilitates DNA break repair by homologous recombination [31]. While it has not been addressed whether proximity to a cohesin binding site affects the efficiency of DNA repair, it is assumed that sister chromatid cohesion at frequent intervals maintains contact between sister chromatids. The mean distance between cohesin binding sites along chromosome 2 was 13.1 kb, the median 16.5 kb. To address whether an organizing principle underlies the distribution of cohesin peaks among the convergent sites, we used a bootstrapping approach to randomize the distribution of 228 peaks among all available convergent sites (Figure S2 in Additional data file 1). The average median distance between cohesin peaks in 10,000 random distributions was 14.5 kb, somewhat closer than the observed median distance. In contrast, the greatest distance between two neighboring binding sites in random distributions ranged from 64.9 and 230.4 kb, on average 104.6 kb, almost twice the actual greatest observed distance between convergent site peaks of 60.9 kb. This suggests that a mechanism exists that ensures even distribution of cohesin binding among convergent sites (*p *< 0.0001).

We also analyzed whether the orientation of ORFs along chromosomes, which defines the pattern of convergent sites, was ordered in any way. A previous analysis in budding yeast found a pattern of ORF orientations close to a random distribution, in which any ORF is equally likely to be followed by an ORF pointing in the same or opposite direction [7]. Along the three fission yeast chromosomes, ORFs are significantly more likely to be followed by an ORF in the opposite (54.13%) than in the same direction (45.87%, χ^2^-test, *p *= 3 × 10^-9^). Consequently, in addition to the non-random distribution of cohesin peaks among the convergent sites, a larger and more frequent number of convergent sites exists along fission yeast chromosomes than expected by chance.

### Gene arrangement and expression, but not Swi6, contribute to defining cohesin's pattern among convergent sites

**Figure 2:**
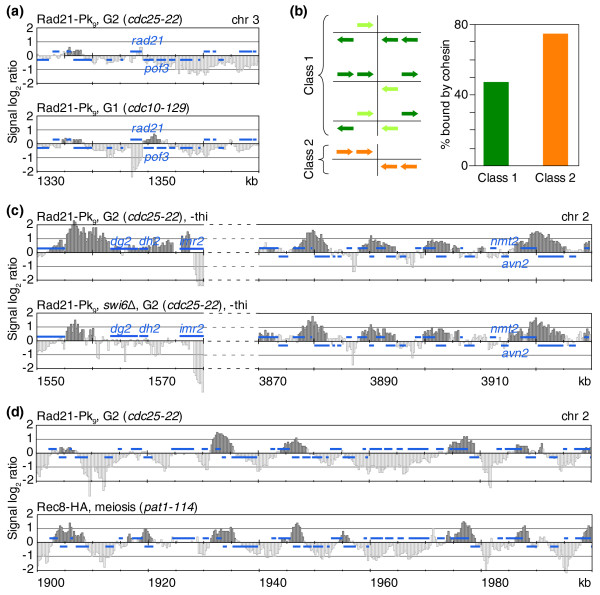
Determinants of cohesin distribution among convergent sites. **(a) **Cohesin translocation and accumulation in response to *rad21 *upregulation. The Rad21-Pk_9 _pattern along a 40 kb region of chromosome 2 is compared in cells arrested in G2 (*cdc25-22*) or G1 (*cdc10-129*) when *rad21 *expression is low or high, respectively. Blue bars above and below the midline represent ORFs transcribed from left to right and opposite, respectively. **(b) **Sites where more than one ORF converge from both sides are more likely bound by cohesin. A schematic of class 1 and 2 convergent sites, and the percentage of these that are bound by cohesin along chromosome 2, are depicted. **(c) **Swi6 contributes to cohesin enrichment at centromeres, but not at convergent sites. The Rad21-Pk_9 _pattern at the centromere (outer, *dg2 *and *dh2*, and inner, *imr2*, centromeric repeat sequences are indicated), and along a 60 kb region on the right arm of chromosome 2, is shown in G2-arrested wild-type or *swi6Δ *cells. Cells were grown in medium lacking thiamine (-thi) to induce *nmt2 *(compare [17]). **(d) **More frequent cohesin occupation of convergent sites in meiosis. The cohesin pattern in G2-arrested cells (Rad21-Pk_9_) is compared to that in cells progressing through meiosis (Rec8-HA) along a 100 kb region on chromosome 2 (Rec8-HA profile from [34]).

To understand why some, but not all, convergent sites are bound by cohesin, we first addressed whether fission yeast cohesin responds to Pol II transcription by downstream translocation, similar to budding yeast [6,7]. As an example, we analyzed cohesin at the convergent site between the *rad21 *and *pof3 *genes. *rad21 *expression is high during G1 and S phase, but low in G2 [32]. In cells arrested in G2 using the *cdc25-22 *allele, only a small cohesin peak was detectable that largely overlapped with the *rad21 *ORF (Figure 2a). In contrast, in cells arrested in G1 using the *cdc10-129 *allele, the *rad21 *ORF was clear of cohesin and instead a large cohesin peak accumulated downstream of *rad21*. This suggests that cohesin responded to *rad21 *transcriptional upregulation with downstream translocation. The increased peak size in response to *rad21 *expression further suggests that active transcription at the convergent site contributes to the accumulation of cohesin. These results are consistent with a recent report of increased cohesin accumulation at the *nmt1*/*gut2 *convergent site on chromosome 3 when *nmt1 *expression is induced [17].

We next tested whether the transcriptional strength of convergent gene pairs generally correlated with cohesin binding. As an indicator for transcriptional activity, we compared the transcript abundance measured under growth conditions similar to those used in our experiments [33]. This revealed that convergent genes surrounding cohesin binding sites are more strongly transcribed than those lacking cohesin (2,325.5 versus 1,848.0 median relative mRNA levels of genes flanking cohesin-bound and cohesin-free convergent sites, respectively, *p *= 0.011; Figure S3 in Additional data file 1). In addition, we noticed that convergent sites with gene runs of more than one ORF in each direction were more likely to be bound by cohesin. We grouped convergent sites along chromosome 2 in two classes, class 1 containing all convergent sites where from one or both sides only one gene terminates (n = 412), and class 2 containing the remaining sites with at least two convergent genes on both sides (n = 96; Figure 2b). While 74% of class 2 convergent sites were bound by cohesin, only 46% of class 1 were. This suggests that in addition to the strength of transcription, the number of genes that point towards a convergent site influences the chance of cohesin binding.

It has been suggested that transcriptional readthrough at convergent sites promotes double stranded RNA-dependent heterochromatin formation, which in turn underlies cohesin recruitment [17]. We therefore tested whether the heterochromatin protein Swi6, thought to recruit cohesin, played a role in generating the observed cohesin pattern. We grew wild-type and *swi6Δ *cells in synthetic medium lacking thiamine, conditions under which the *nmt2 *gene is transcribed at the *nmt2*/*avn2 *convergent site that has been studied as an example. In contrast to the prediction, the cohesin pattern along chromosome arms, including the *nmt2*/*avn2 *convergent site, remained unchanged in *swi6Δ *cells (Figure 2c, right). We detected only small amounts of cohesin at the *nmt2*/*avn2 *convergent site, a class 1 convergent site following the above classification. At the centromeric repeats, cohesin levels were reduced in the absence of Swi6 (Figure 2c, left). These findings are consistent with previous reports implicating Swi6 in cohesin recruitment to centromeric heterochromatin, but not chromosome arms [15,16].

We also compared the observed cohesin pattern during the mitotic cell cycle with that during meiosis, when the Rad21 subunit is largely replaced by its meiotic paralog Rec8 [34]. Figure 2d shows that during meiosis, cohesin appears to be more uniformly distributed among convergent sites; 346 (68%) of all convergent sites along chromosome 2 were cohesin-bound in meiosis, compared to 262 (52%) during mitosis. This difference could stem in part from an altered transcriptional profile during meiosis, or could be the consequence of a distinct chromosome architecture during meiotic prophase - for example, a specific requirement of cohesin for homolog pairing or recombination.

### The Mis4/Ssl3 cohesin loader coincides with highly transcribed genes

**Figure 3:**
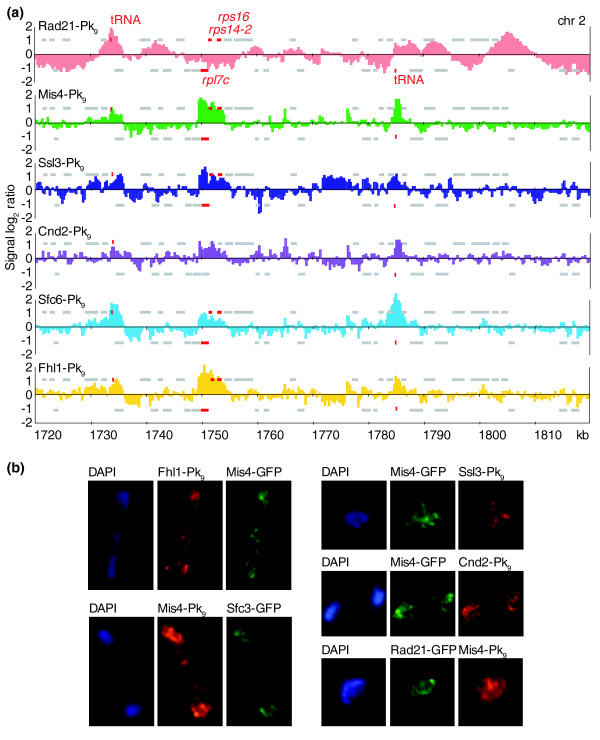
The cohesin loader Mis4/Ssl3 partly colocalizes with cohesin at tRNA and ribosomal protein genes. **(a) **Comparison of Rad21, Mis4, Ssl3, Cnd2, Sfc6 and Fhl1 localization, all fused to a Pk_9 _epitope tag, by chromatin immunoprecipitation. Cnd2 was analyzed in an *nda3-KM311 *strain arrested in mid-mitosis at the restrictive temperature, and the other proteins were analyzed in exponentially proliferating cells. A 100 kb region of chromosome 2 is shown. Grey bars above and below the midline represent ORFs transcribed from left to right and opposite, respectively. tRNA and ribosomal protein genes are highlighted in red. **(b) **Cytological colocalization analysis. Spread chromosomes were stained with antibodies against the Pk epitope tag, and against GFP, to detect the indicated pairs of proteins. DNA was counterstained with DAPI.

To compare cohesin's pattern to its likely sites of chromosomal loading, we analyzed the localization of the cohesin loader subunits Mis4 and Ssl3. The two subunits showed a largely overlapping pattern of binding (Figure 3a; Figure S4 in Additional data file 1). The pattern along chromosome arms remained unchanged between exponentially proliferating wild-type cells and *cdc10-129 *cells arrested in G1, a cell cycle phase that is important for cohesin loading. At core centromeric sequences, we detected strong Mis4 binding in proliferating cells that was less pronounced in the G1 arrest (Figure S5 in Additional data file 1). Using our peak picking algorithm, we detected 72 peaks of Mis4 binding along the arms of chromosome 2. Mis4/Ssl3 showed a largely distinct binding pattern from that of cohesin. The peaks were generally sharper (3.4 kb average width, compared to 6.5 kb for cohesin) and only 30 overlapped with convergent sites. Despite the largely distinct positions of the peak maxima, 56 of the Mis4/Ssl3 peaks showed an overlap with a broad cohesin peak. In the search for an underlying determinant of Mis4/Ssl3 binding sites, we noticed a striking correlation with tRNA and ribosomal protein genes, which we have also observed for the budding yeast cohesin loader Scc2/Scc4 (Figure 3a; Figure S4 in Additional data file 1) [24]. Of the 72 peaks identified, 34 were assigned within 5 kb of a tRNA or ribosomal protein gene. Upon visual inspection, virtually all 38 tRNA and 42 ribosomal protein genes along the arms of chromosome 2 were found associated with Mis4/Ssl3, even though some of the peaks fell below the detection threshold of our peak picking algorithm. The colocalization of the cohesin loader Mis4/Ssl3 with tRNA genes is also apparent at the tRNA gene clusters flanking fission yeast centromeres, which were excluded from the above analysis (Figure S4 in Additional data file 1).

Mis4/Ssl3 binding sites at tRNA genes overlapped with the binding profile of the Pol III transcription factor TFIIIC subunit Sfc6, while at ribosomal protein genes it colocalized with the forkhead domain containing protein Fhl1 (Figure 3a). Fhl1 is a possible fission yeast ortholog of the transcription factor Fhl1 that controls ribosomal protein gene expression in budding yeast [35]. We found Sfc6 also associated with ribosomal protein genes, and Fhl1 with tRNA genes, though with weaker signal intensities (Figure 3a). We do not currently know whether Sfc6 contributes to transcriptional control of ribosomal protein genes, and Fhl1 to that of tRNA genes, or whether the weaker levels of association may reflect indirect association, mediated by interactions between ribosomal protein and tRNA gene loci. Similar to budding yeast, Mis4/Ssl3 binding sites are also binding sites for the chromosomal condensin complex, which may mediate such interactions (Figure 3a) [24,36].

To analyze the localization patterns of the above proteins using a complementary technique, we performed immunostaining of spread chromosomes. The staining patterns obtained were consistent with colocalization of Mis4 with Ssl3, condensin, the TFIIIC subunit Sfc3 and Fhl1, and confirmed a largely distinct localization of Mis4 and cohesin (Figure 3b).

tRNA and ribosomal protein genes are strongly transcribed by Pol III and Pol II, respectively. Together, these loci account for approximately half of the detected Mis4/Ssl3 binding sites. We therefore tested whether Mis4/Ssl3 binding sites were generally characterized by high expression levels. The median mRNA level of genes bound by Mis4/Ssl3, excluding tRNA genes, was almost twice the median of all unbound genes (2,918 versus 1,559, *p *< 10^-12^, Wilcoxon signed ranks test; genes with expression level '0' were excluded from the analysis; Figure S6 in Additional data file 1). When the strongly transcribed ribosomal protein genes were excluded from the analysis, the remaining Mis4/Ssl3 bound genes still showed significantly higher expression than the unbound genes (median mRNA level 2,331, *p *< 10^-5^). However, not all highly expressed genes are Mis4/Ssl3 binding sites; 389 genes along chromosome 2 showed mRNA levels greater than the median of all Mis4/Ssl3-bound genes, yet are not associated with the cohesin loader. This indicates that Mis4/Ssl3 associates with highly expressed genes, but that a high level of transcription is not a sufficient determinant for binding. Rather, certain groups of highly transcribed genes may contain features - for example, promoter elements or associated transcription factors - that form a cohesin loading site.

### The relationship between cohesin and its loading sites

**Figure 4:**
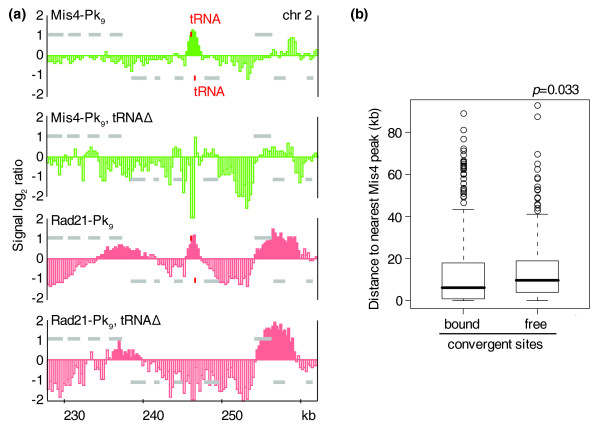
The relationship between cohesin and its loader. **(a) **Deletion of two tRNA genes removes a cohesin loading site, but does not affect the surrounding cohesin pattern. Mis4-Pk_9 _and Rad21-Pk_9 _association over a 35 kb region of chromosome 2 is compared between strains containing, or deleted for, the two tRNA genes SPBTRNAARG.04 and SPBTRNAGLY.05. Grey bars above and below the midline represent ORFs transcribed from left to right and opposite, respectively. The two tRNA genes are highlighted in red. **(b) **Distance distribution of cohesin-bound and cohesin-free convergent sites to their nearest Mis4 binding sites. Boxes indicate boundaries of the 25th to 75th percentile surrounding the median (solid line). Whiskers encompass 1.5 times the interquartile range, outliers are indicated. A Wilcoxon signed ranks test suggests that cohesin-bound convergent sites are closer to their nearest loading site than cohesin-free convergent sites.

Having identified the binding sites of the Mis4/Ssl3 cohesin loader, we analyzed its relationship with the cohesin distribution along chromosome arms. This revealed that the 14 cohesin peaks along chromosome 2 that did not overlap with a convergent site coincided with Mis4/Ssl3; seven of these are at tRNA gene loci (for example, Figure 4a), and one is at a ribosomal protein gene. At three of the seven tRNA loci the direction of Pol III-mediated transcription converges with, while at the remaining sites the tRNA gene(s) are parallel with or divergent from, the surrounding genes. In addition, we counted 15 instances where Mis4/Ssl3 coincided with a shoulder of a cohesin peak that extended beyond a convergent site (for example, Figure 3a at 1,785 kb). This suggests that at least some of cohesin's loading sites also function as cohesin binding sites. These observations are reminiscent of the distribution of cohesin and its loader subunit Nipped-B in *Drosophila*, where the two proteins show a largely overlapping pattern close to highly expressed genes. We do not currently know whether cohesin that we detect at its loading sites represents the stably loaded complex, or reflects an intermediate of loading prior to its translocation.

We next asked whether the distribution of loading sites also influences the cohesin pattern among convergent sites. We therefore compared the distances of convergent sites that were bound by cohesin, or not, from their respective nearest Mis4/Ssl3 binding sites. The median distance of cohesin-bound convergent sites to the nearest Mis4/Ssl3 site was 3.9 kb, significantly less than convergent sites that were free of cohesin (7.4 kb, *p *= 0.033; Figure 4b). This suggests that the loading site distribution contributes to defining the cohesin pattern along chromosomes arms.

If Mis4/Ssl3 association sites promote cohesin binding in their surroundings, deletion of a loading site should alter the cohesin pattern. To test this, we deleted a 489 bp sequence containing two adjacent tRNA genes that form a Mis4/Ssl3 and cohesin binding site on the left arm of chromosome 2 (Figure 4a). In response to the deletion, Mis4/Ssl3 binding to this locus disappeared. Cohesin was also no longer detected at this site, consistent with the notion that cohesin loading had been disrupted. However, the cohesin distribution at convergent sites in the vicinity of the former loading site remained unchanged. This suggests that establishment of the overall cohesin pattern in the surroundings did not depend on this specific Mis4/Ssl3 binding site. Despite the tRNA deletion, residual Mis4/Ssl3 remained detectable in the vicinity (Figure 4a). Cohesin loading by Mis4/Ssl3 might therefore be less restricted to its peaks of binding than the pattern suggests. Alternatively, other determinants - for example, gene orientation and transcriptional activity - might define cohesin distribution at this locus.

### Prophase removal and cohesin cleavage during mitosis

**Figure 5:**
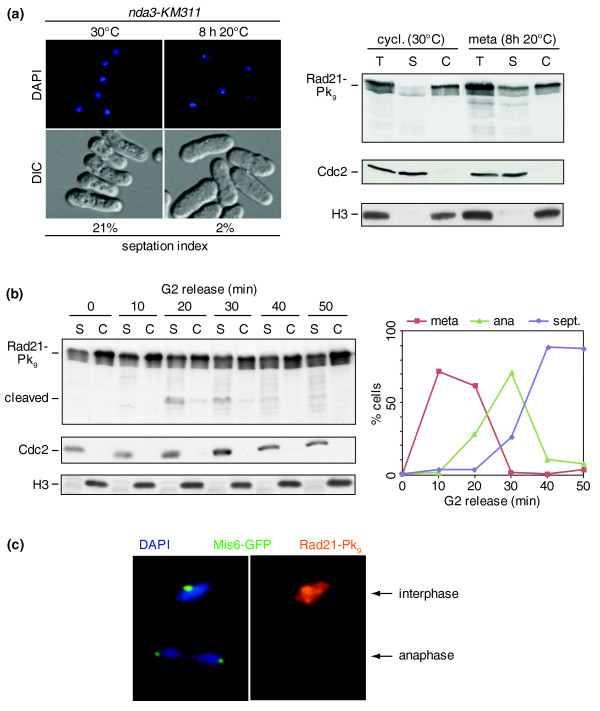
Cohesin removal from chromosomes during mitosis. **(a) **A small increase of soluble cohesin in mitosis. Total cell extracts (T) of an *nda3-KM311 *strain, either during exponential growth when most cells are in G2, or after arrest in mid-mitosis with condensed chromosomes at 20°C, were separated into soluble (S) and chromosome-bound (C) fractions. The distribution of Rad21-Pk_9 _was analyzed by western blotting. Cdc2 and histone H3 served as soluble and chromosomal loading controls, respectively. DAPI-stained condensed mitotic chromosomes, and the low septation index, confirmed the arrest. DIC, differential interference contrast. **(b) **Prophase dissociation and anaphase (ana) cleavage of cohesin during synchronous mitotic progression. The cell fractionation experiment was repeated at time points after release of cells from a *cdc25-22*-imposed G2 arrest. Mitotic progression was monitored by spindle morphology and septation (sept.) index. **(c) **Residual chromosomal cohesin during anaphase. Chromosome spreads from an exponentially growing culture were stained against Rad21-Pk_9_. The kinetochore marker Mis6-GFP was used to identify chromosomes in anaphase.

We next analyzed cohesin behavior along fission yeast chromosomes during loss of sister chromatid cohesion in mitosis. It is thought that only a small part of fission yeast cohesin is cleaved as sister chromatids split at anaphase onset [3], so we asked whether part of cohesin is removed from chromosomes already during prophase, as observed in higher eukaryotes [29,30]. To test this, we compared the cohesin distribution between chromatin-bound and soluble fractions as cells enter mitosis. In an exponentially growing population of cells, carrying the cold-sensitive β-tubulin mutation *nda3-KM311*, almost all cohesin was found chromatin-bound (Figure 5a). After these cells were arrested in mid-mitosis with condensed chromosomes at restrictive temperature, we detected increased levels of soluble cohesin, while a large fraction of cohesin remained chromatin-associated. This suggests that a small fraction of cohesin dissociates from chromosomes in mitosis, while the majority of cohesin remains chromosome-bound.

In order to follow cohesin through mitosis, we arrested a cell population in G2 using the thermosensitive *cdc25-22 *mutation and released the culture into synchronous mitotic progression at permissive temperature. As cells entered mitosis (10 minutes), we again detected increased cohesin levels in the soluble fraction, consistent with removal of a small cohesin pool during prophase (Figure 5b). At the time of anaphase (20-30 minutes), Rad21 cleavage products became detectable and chromosomal cohesin levels dropped by about half. As the carboxy-terminal Rad21 cleavage fragments are expected to be rapidly turned over by the N-end rule pathway [37], it is hard to estimate the total amount of cohesin cleaved during anaphase. Considering the amount of cohesin loss at this time, we estimate that up to half of the cohesin might be cleaved. The appearance of the cleavage product mainly in the soluble fraction is consistent with the idea that cohesin dissociates from chromosomes after cleavage. The loss of cohesin from chromosomes during anaphase could also be visualized by immunostaining of cohesin on spread chromosomes. Cells in anaphase displayed a distinctly reduced cohesin signal, consistent with its removal from chromosomes (Figure 5c). A lower level of cohesin remained visible even on anaphase chromosomes, confirming that a subpool of cohesin is not removed from chromosomes during anaphase and, therefore, may not have participated in sister chromatid cohesion.

### Reciprocal regulation of Mis4/Ssl3 and condensin during mitosis

**Figure 6:**
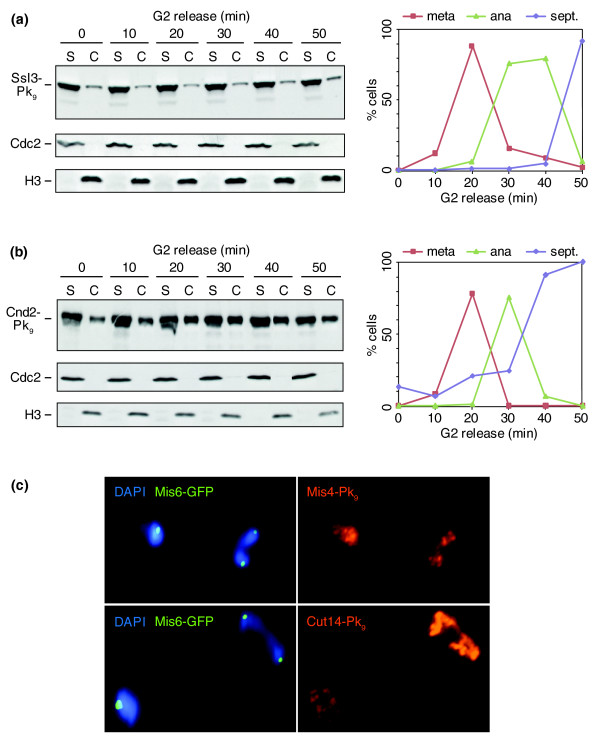
Chromosome association of the cohesin loader and condensin during mitosis. **(a) **Cell fractionation of a culture progressing through synchronous mitosis after release from *cdc25-22 *arrest was performed as in Figure 5b. The distribution of the cohesin loader subunit Ssl3-Pk_9 _was analyzed. **(b) **As (a), but the chromosomal association of the condensin subunit Cnd2-Pk_9 _was detected. **(c) **Cytological comparison of the staining intensities of the cohesin loader subunit Mis4-Pk_9_, and the condensin subunit Cut14-Pk_9_, on spread chromosomes. Anaphase cells were identified among cells in other cell cycle stages by two segregating Mis6-GFP signals.

It has been observed in vertebrate cells that the cohesin loader complex dissociates from chromosomes as cells enter mitosis, while it remains constitutively chromosome-bound throughout the cell cycle in budding yeast [22,25,38]. To study the behavior of the fission yeast Mis4/Ssl3 cohesin loader during mitosis, we repeated the chromatin fractionation experiment and analyzed the subcellular distribution of Ssl3. In G2-arrested cells, the majority of Ssl3 resided in the soluble fraction, while a subfraction was chromatin-bound (Figure 6a). This distribution was similar to that observed in an exponentially proliferating cell population (data not shown). Upon synchronous release into progression through mitosis, there was no marked change and Ssl3 remained detectable on chromosomes throughout the time-course. We also analyzed Mis4/Ssl3 localization by staining spread chromosomes. The kinetochore marker Mis6 served to identify spreads from cells in mitosis when chromosomes segregate. This confirmed that Mis4 remains chromatin-bound throughout mitosis (Figure 6c).

Because of the colocalization of Mis4/Ssl3 with condensin, and the role of the budding yeast cohesin loader in mitotic chromosome condensation [24], we analyzed the binding pattern of fission yeast condensin with chromosomes during mitosis. As fission yeast cells enter mitosis, condensin accumulates in the nucleus due to mitotic phosphorylation of its Cut3 subunit [39]. In G2 cells, a small but significant fraction of condensin fractionated with the chromosome-bound material. This fraction increased during mitosis when approximately half of cellular condensin was chromosome-bound (Figure 6b). The increase of chromosomal condensin in mitosis could also be clearly seen on immunostained spread chromosomes (Figure 6c). This suggests that as cells enter mitosis, Mis4/Ssl3 is retained on chromosomes while levels of condensin increase.

### Spreading of centromeric cohesin onto chromosome arms during mitosis

We next investigated whether cohesin removal in mitosis affected certain regions of the chromosome more than others. We therefore followed the cohesin binding pattern in cells synchronously progressing through mitosis by chromatin immunoprecipitation. The cohesin pattern remained largely unchanged throughout G2, metaphase and anaphase, although the height of peaks along chromosome arms decreased (Figure S7 in Additional data file 1). This suggests that cohesin is not removed from a subset of its binding sites during mitosis, but that its association among most binding sites was uniformly reduced.

**Figure 7:**
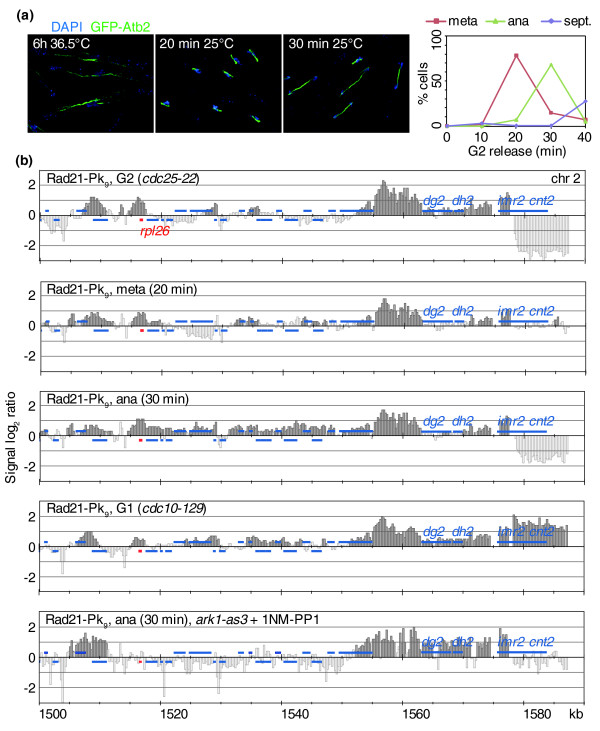
Aurora B kinase-dependent spreading of centromeric cohesin during mitosis. **(a) **Synchronous progression through mitosis after release from *cdc25-22*-imposed G2 arrest. Examples of cells at the indicated time points are shown. Microtubules were visualized by a GFP-Atb2 fusion protein; DNA is counterstained with DAPI. The percentages of cells with short metaphase (meta) or elongated anaphase (ana) spindles, as well as the septation (sept.) index, were counted in at least 100 cells at each time point. **(b) **Chromatin immunoprecipitation against Rad21-Pk_9 _during the timecourse in (a) is compared to G1-arrested cells, and to *cdc25-22*-synchronized anaphase cells lacking aurora B kinase activity due to chemical inhibition of *ark1-as3*. A 100 kb region including the centromere of chromosome 2 is shown. Outer, *dg2 *and *dh2*, and inner, *imr2*, centromeric repeat sequences and the central core, *cnt2*, are indicated. Additional blue bars above and below the midline represent ORFs transcribed from left to right and opposite, respectively. A ribosomal protein gene is highlighted in red.

A noticeable change to the cohesin pattern during mitosis became apparent around centromeres. Over a region of approximately 50 kb surrounding the centromeres, cohesin appeared to spread along chromosome arms, showing little preference for convergent sites (Figure 7). The spreading started as cells entered metaphase and became most prominent during anaphase. We also observed a similar, although less pronounced, spreading around centromeres in cells arrested in G1 using the *cdc10-129 *thermosensitive mutation. This suggests that spreading of cohesin around centromeres is cell cycle stage specific. Cohesin is enriched at the centromeric repeat regions, tethered by the heterochromatin protein Swi6 [15,16]. In mitosis, Swi6 levels are reduced after aurora B kinase-dependent phosphorylation of histone H3, and it reaccumulates as cells enter the subsequent S phase [40]. To test whether loss of Swi6-dependent cohesin anchoring at centromeric repeats could be the reason for cohesin spreading onto chromosome arms, we repeated the above experiment but inhibited the activity of aurora B kinase using an ATP analog (1NM-PP1)-sensitive allele of the kinase, *ark1-as3 *[41]. When 5 μM 1NM-PP1 was added to G2-arrested *ark1-as3 *cells 15 minutes before their release into progression through mitosis, cohesin spreading during anaphase was no longer observed. Compared to *ark1 *wild-type control cells, heterochromatic accumulation of Swi6-green fluorescent protein (GFP) remained detectable throughout mitosis after Ark1 inhibition (data not shown). Therefore, as Swi6 dissociates from centromeres during mitosis, cohesin is mobilized and able to spread out onto neighboring sequences. The activity of aurora B kinase also promotes mitotic condensin enrichment with fission yeast chromosomes [42], which could also contribute to the observed spreading of centromeric cohesin.

## Discussion

Cohesin is an essential chromosomal protein complex with ubiquitous roles in chromosome segregation, DNA repair and transcriptional regulation. Despite its conserved function, recent studies have revealed striking apparent disparities between its chromosomal association patterns in different organisms. Our analysis of cohesin localization along fission yeast chromosomes has contributed to reconciling different aspects of cohesin behavior on chromosomes and suggests that differences between organisms may reflect an emphasis on individual aspects of cohesin behavior rather than different principles of action.

Cohesin loading onto chromosomes starts in a reaction catalyzed by the Mis4/Ssl3 (Scc2/Scc4; Nipped-B) complex. How the Mis4/Ssl3 complex is recruited to chromosomes is still largely unknown, but in budding yeast and *Drosophila *its binding sites correlate with strongly expressed genes [7,14,24]. We find that the same is true in fission yeast, although strong transcription by itself is not sufficient to create a Mis4/Ssl3 binding site. Rather, certain features of a subset of strongly transcribed genes might make them cohesin loading sites, the nature of which remains to be elucidated. In *Drosophila*, the chromosomal association pattern of the cohesin complex largely overlaps with that of its loading factor [14]. In contrast, budding yeast cohesin is thought to translocate away from its loading sites to accumulate in regions of convergent transcriptional termination [7]. In fission yeast, we see evidence for both retention of cohesin at a subset of its loading sites as well as translocation to convergent sites. This opens the possibility that cohesin associates with chromosomes in the same way in these organisms, with more or less pronounced retention at its loading sites. While no evidence for cohesin translocation to convergent sites has been obtained along *Drosophila *chromosomes, we cannot exclude that a similar process takes place. Intergenic regions at convergent sites are larger in higher eukaryotes, as compared to the yeasts, so that cohesin in these regions may not form peaks of association that can be easily detected. We do not yet know the location of human Scc2/Scc4 along chromosomes, and whether cohesin's peaks of association at CTCF binding sites are indicative of its loading sites or of locations distinct from the loader. Apparently normal total levels of cohesin on chromosomes in the absence of CTCF suggest that CTCF might act as a cohesin anchor, as exemplified by fission yeast Swi6, rather than a loading factor [9].

Cohesin fulfils roles both in sister chromatid cohesion as well as transcriptional regulation. It is possible that cohesin's function at its loading sites, and at distant convergent sites, differs. Cohesin at its loading site may maintain a more dynamic state of binding, conducive for promoting reversible interactions between gene regulatory regions. tRNA genes, which we identified as cohesin loading sites in budding and fission yeast, are known transcriptional insulators [43]. The *Drosophila *cohesin loader Nipped-B together with cohesin also regulate transcriptional insulation [12], and the identification of cohesin as a component of human CTCF insulators suggests that this aspect of cohesin function is conserved [9,10]. At convergent sites, distant from the loading sites, cohesin may reach a more stable mode of interaction required for enduring sister chromatid cohesion.

The distribution of cohesin binding sites along fission yeast chromosome arms is not random and shows signs of an ordered arrangement. Only about half of all convergent sites are bound by cohesin, and these are spaced such that large gaps between binding sites are avoided. This appears to be achieved not by a single mechanism, but by a number of factors, including gene arrangement, transcription levels and proximity to a loading site. Of note, the gene arrangement along fission yeast chromosomes favors alterations in gene orientation at the expense of parallel gene runs. As a consequence, the fission yeast genome contains 107 more convergent sites as possible cohesin binding sites than expected by chance. We do not know whether the benefit of regularly spaced cohesin along chromosome arms contributed to the selection of this non-random gene order. Non-randomness of the gene orientation is less pronounced in budding yeast [7]. In this organism, virtually all convergent sites are cohesin-bound, thus reducing the possible impact of parallel gene runs. The higher frequency of cohesin binding sites in budding yeast could be the consequence of a larger number of loading sites. In comparison to 274 tRNA genes in budding yeast, the fission yeast genome contains only 196 tRNA genes, many of which are clustered close to centromeres.

As cells enter mitosis, a small fraction of cohesin is released from chromosomes in an apparently cleavage-independent reaction. This could be related to the prophase pathway of cohesin removal that is observed in higher eukaryotes [29,30]. At anaphase onset, a larger proportion of cohesin is lost from chromosomes as cohesin is cleaved. The distribution of the remaining chromosomal cohesin stays largely unchanged along chromosome arms. This suggests that cohesin removal in prophase and anaphase does not affect specific cohesin locations, but uniformly acts on all chromosome arm-bound cohesin. A substantial part of cohesin remains associated with chromosomes throughout mitosis, which is therefore unlikely to be involved in sister chromatid cohesion. It could be that this pool of cohesin associated with chromosomes during G2, which in fission yeast takes up a large part of the cell cycle, and therefore did not participate in the establishment of sister chromatid cohesion during S phase. This pool may promote cohesin functions outside of sister chromatid cohesion - for example, transcriptional regulation - which may continue throughout mitosis. Whether and how separase is able to discriminate during cohesin cleavage between molecules that are, or are not, involved in sister chromatid cohesion are not known. The recent identification of Smc3 acetylation as a cohesin modification that promotes sister chromatid cohesion opens the possibility for such a distinction [44,45]. Cohesin that persists on chromosomes during mitosis may then participate in cohesion establishment in the subsequent S phase, thus supplementing new cohesin loading during the short fission yeast G1 phase.

At centromeres, we observed that cohesin spreads out onto chromosome arms during mitosis. This could be because Swi6-dependent anchoring at the outer centromeric repeats is lost at this time. Initial cohesin tethering to the outer centromeric repeats ensures persisting cohesin enrichment around the centromere, which forms the basis for bi-orientation of sister chromatids on the mitotic spindle [15,16]. Release from this tether in mitosis may, in turn, facilitate the dynamic centromere breathing behavior that is part of the bi-orientation process [46]. Whether the enrichment of the cohesin loader Mis4/Ssl3 at core centromeric sequences plays a role in mitotic cohesin behavior remains to be investigated. These considerations suggest a possible explanation for the phenotype of mammalian cells lacking centromeric heterochromatin [18,19]. Cohesin is still found enriched at centromeres, suggesting that cohesin loading occurs independently of heterochromatin. Nevertheless, centromere cohesion may be compromised because cohesin anchoring to its underlying DNA sequences is lost, resulting in the observed reduction of the centromeric constriction. A fuller understanding of cohesin behavior at higher eukaryotic centromeres will have to await identification of the DNA sequences that it associates with.

## Conclusions

Using high resolution chromosome-wide analysis of the localization of fission yeast cohesin and its loading factor, we describe previously unappreciated principles of eukaryotic chromosome organization. Transcriptional features along chromosomes describe both the loading sites as well as determinants of the final pattern of cohesin localization. Our analysis goes towards reconciling distinct characteristics of cohesin binding in different model organisms. Cohesin is retained at a subset of its loading sites, with the potential of contributing to transcriptional insulation, while also responding to active transcription by accumulation at convergent sites. The behavior of fission yeast cohesin during mitosis underscores the existence of cohesin subpools with different dynamic properties along chromosomes.

## Materials and methods

### Strains, media and molecular genetics

A detailed strain list can be found in Table S1 in Additional data file 1. Gene deletions and epitope tagging were performed by gene targeting using PCR products [47,48]. Deletion of the two tRNA genes SPBTRNAGLY.05 and SPBTRNAARG.04 on the left arm of chromosome 2 was achieved by replacing the region between coordinates 326,235 and 326,724 (as annotated in GeneDB) with a *Kluyveromyces lactis URA3 *auxotrophic marker, complementing *Schizosaccharomyces pombe ura4*, flanked by 142 bp direct repeats [49]. The *URA3 *insertion was then lost after counterselection on 5-fluoroorotic acid, leaving one 142 bp repeat behind. Cells were grown in YE4S rich medium or EMM2 minimal medium with amino acid supplements at 25°C [50].

### Cell cycle synchronization

For G2 arrest, strains carrying the thermosensitive *cdc25-22 *allele were shifted to 36.5°C for 6 hours in EMM2 medium. Subsequent synchronous release into progression through mitosis was achieved by rapid shaking of the culture flasks in ice-water until the medium cooled down to 25°C and further incubation at this temperature. Arrest and release were monitored by spindle morphology and the septation index. Mitotic metaphase arrest was achieved in strains bearing the *nda3-KM311 *cold-sensitive mutation by shifting the cultures to 20°C for 6-8 hours in YE4S medium. G1 arrest was performed with cells carrying the *cdc10-129 *allele, shifted to 36°C for 4 hours in YE4S. G1 arrest was monitored by flow cytometric analysis of DNA content.

### Chromosome spreading and cell fractionation

Chromosome spreading was performed as described previously [51]. For cell fractionation into soluble and chromosome-bound pools, spheroplasts were prepared as for chromosome spreads and processed as described [52], but spheroplasts were resuspended in 20 mM HEPES/KOH pH 7.9, 1.5 mM MgAc, 50 mM KAc, 10% glycerol, 0.5 mM dithiothreitol (DTT), 1 mM phenylmethylsulphonyl fluoride (PMSF), 2.4 μg/ml chymostatin, 20 μg/ml leupeptin, 40 μg/ml aprotinin, 1.5 μg/ml pepstatin, 2 mM benzamidine and lysed by addition of 1% Triton X-100.

### Chromatin immunoprecipitation

Chromatin immunoprecipitation analysis was performed as described [7]. In brief, 2 × 10^9 ^cells were fixed in 1% formaldehyde for 30 minutes at room temperature. A multibead shocker (Yasui Kikai, Osaka, Japan) was used to prepare cell extracts by glass bead breakage, followed by sonication to retrieve 400-800 bp long DNA fragments. α-HA mouse monoclonal antibody clone 16B12 (Roche, Mannheim, Germany) or α-Pk clone SV5-Pk1 (Serotec, Kidlington, UK) were added, and immunocomplexes adsorbed to protein A coupled magnetic beads (Dynal Biotech ASA, Oslo, Norway). SDS eluates were incubated overnight at 65°C to reverse the crosslink, and DNA fragments were purified and amplified using random prime PCR. After biotin labeling, the DNA cocktail was hybridized to oligonucleotide tiling arrays covering fission yeast chromosomes 2 and 3 (S_pombea520106F, Affymetrix P/N 520106). The microarray data in this report have been deposited with the Gene Expression Omnibus, accession number [GEO:GSE13517].

### Other techniques

DNA content was measured by flow cytometry of propidium iodide-stained ethanol-fixed cells using a FACScanner (Becton Dickinson, Franklin Lakes, NJ, USA). For microscopy, DNA was stained with 4',6-diamidino-2-phenylindole (DAPI), septa were stained with calcofluor and microtubules were visualized in methanol-fixed cells expressing a GFP-Atb2 fusion protein. 1NM-PP1 (Toronto Research Chemicals Inc., cat. No. A603003, North York, On., Canada), used to inhibit the *ark1-as3 *allele, was diluted into the culture medium from a 1 mM stock solution in DMSO. Antibodies used for cytology and western blotting were α-Pk clone SV5-Pk1 (Serotec), α-GFP clone TP401 (Torrey Pines Biolabs, Houston, TX, USA), α-histone H3 antiserum ab1791 (Abcam, Cambridge, UK) and α-PSTAIRE serum, recognizing Cdc2, sc-53 (Santa Cruz Biotechnology, Santa Cruz, CA, USA).

## Abbreviations

DAPI: 4',6-diamidino-2-phenylindole; GFP: green fluorescent protein; HP1: heterochromatin protein 1; ORF: open reading frame; Pol II: RNA polymerase II; Pol III: RNA polymerase III.

## Authors' contributions

CKS performed the experiments and drafted the manuscript, NB carried out the statistical analyses, FU supervised the project, all authors read and approved the final manuscript.

## Additional data files

The following additional data are available with the online version of this paper: a PDF containing Figures S1-S7 and Table S1 (Additional data file 1).

## Supplementary Material

Additional data file 1 Figure S1 shows cohesin association along fission yeast chromosome 2. Figure S2 is a graph depicting the maximal distance analysis between neighboring cohesin peaks. Figure S3 is a graph demonstrating higher expression levels of cohesin-bound convergent gene pairs. Figure S4 shows Mis4 and Ssl3 association along chromosome 2 and analyzing their overlap with tRNA and ribosomal protein genes. Figure S5 compares the Mis4 association pattern in exponentially growing and G1 arrested cells. Figure S6 is a graph depicting Mis4/Ssl3 binding site correlation with strongly expressed genes. Figure S7 shows that the cohesin pattern along chromosome arms remains qualitatively unchanged during cohesin removal in mitosis. Table S1 contains details on the yeast strains used in this study. Click here for file
