# Supplementary material for: Conserved features of cohesin binding along fission yeast chromosomes
Source: Genome Biol. 2009 May 19;10(5):R52. doi: 10.1186/gb-2009-10-5-r52 (PMC2718518; doi:10.1186/gb-2009-10-5-r52)
Supplement: Additional data file 1 — Figure S1 shows cohesin association along fission yeast chromosome 2. Figure S2 is a graph depicting the maximal distance analysis between neighboring cohesin peaks. Figure S3 is a graph demonstrating higher expression levels of cohesin-bound convergent gene pairs. Figure S4 shows Mis4 and Ssl3 association along chromosome 2 and analyzing their overlap with tRNA and ribosomal protein genes. Figure S5 compares the Mis4 association pattern in exponentially growing and G1 arrested cells. Figure S6 is a graph depicting Mis4/Ssl3 binding site correlation with strongly expressed genes. Figure S7 shows that the cohesin pattern along chromosome arms remains qualitatively unchanged during cohesin removal in mitosis. Table S1 contains details on the yeast strains used in this study. [file gb-2009-10-5-r52-S1.pdf]

**Figure S1**

Cohesin association along fission yeast chromosome 2. (**a**) Association profile of cohesin along chromosome 2. *cdc25-22* cells were arrested in G2 by shifting to the restrictive temperature of 36.5° C for six hours in minimal EMM2 medium and processed for ChIP against the epitope-tagged cohesin subunit Rad21-$Pk_9$. The binding profile of Rad21-$Pk_9$ along fission yeast chromosome 2, relative to a whole genome DNA sample, is shown. The y-axis scale is $\log_2$. Every bar represents the average of 11 oligonucleotide probes within 250 bp windows. Peaks in solid red were assigned according to the following criteria: Signal intensities were smoothed using a sliding 2.25 kb window. Local maxima were identified, and those with a raw signal intensity above 0.3 were chosen as a peak. Peaks extend from their maximum to both sides until the raw signal $\log_2$ ratio reaches a value below 0. The peak position was defined as the midpoint within the peak. Using these criteria 228 peaks were identified along chromosome 2. The illustrated cohesin binding map shows all open reading frames (ORFs) as grey bars above and below the midline, transcribed from left to right and right to left, respectively. tRNA genes, and genes encoding protein components of the large (*rpl*) and small (*rps*) ribosomal subunit are highlighted in red. The coordinates are of the NCBI fission yeast genome release NC003423.1 (25 Jan 2005). (**b**) List of the cohesin peaks identified in (a). The position, average height and width of each of the 228 peaks is indicated.

(a)

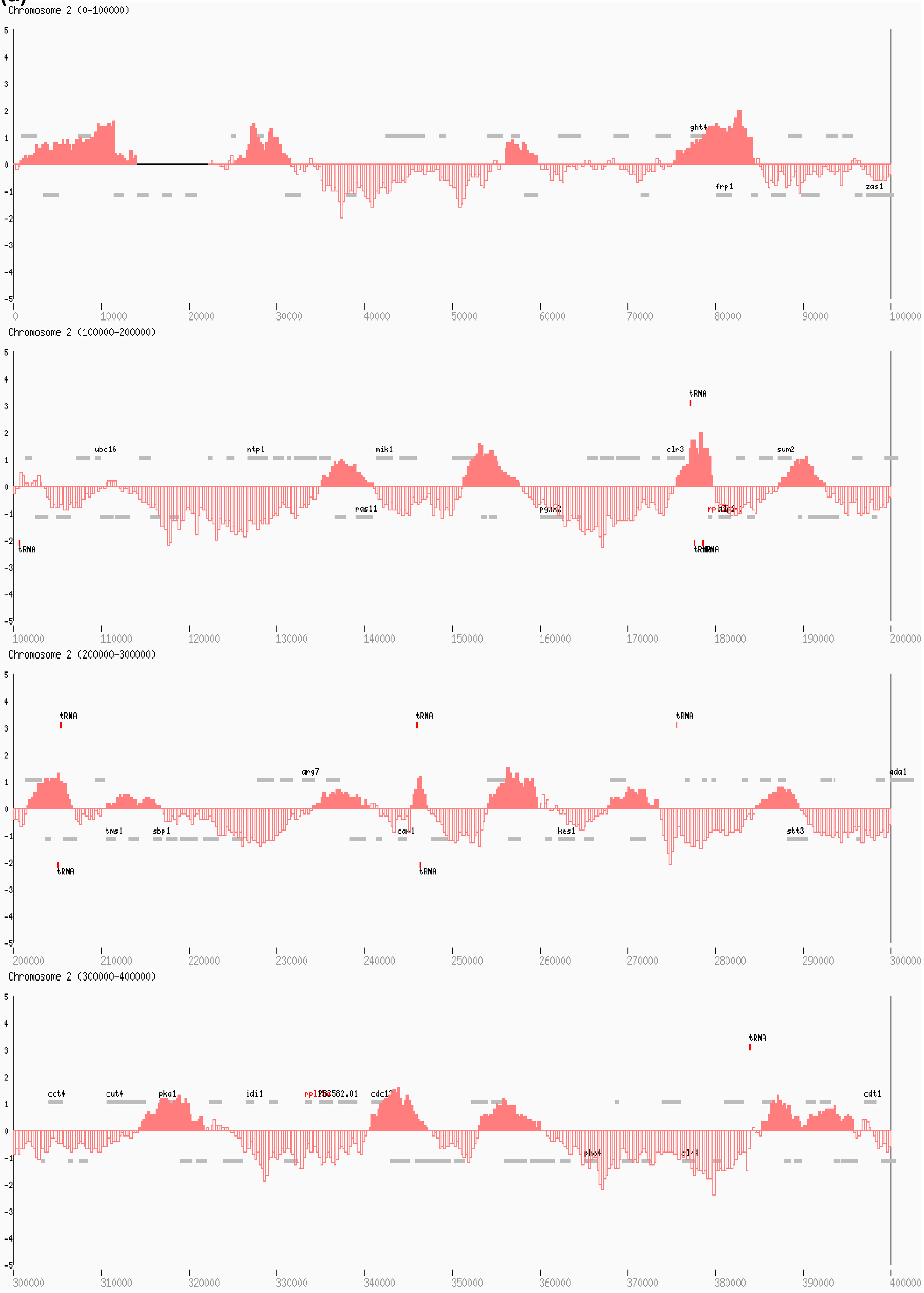
Chromosome 2 (0-100000)
ght4
frp1
zas1
Chromosome 2 (100000-200000)
tRNA
ubc16
ntp1
mik1
clr3
sum2
ras11
Chromosome 2 (200000-300000)
tRNA
arg7
ada1
tms1
sbp1
cam1
kes1
stt3
Chromosome 2 (300000-400000)
tRNA
cct4
cut4
pka1
idi1
cdt1

Chromosome 2 (400000-500000)

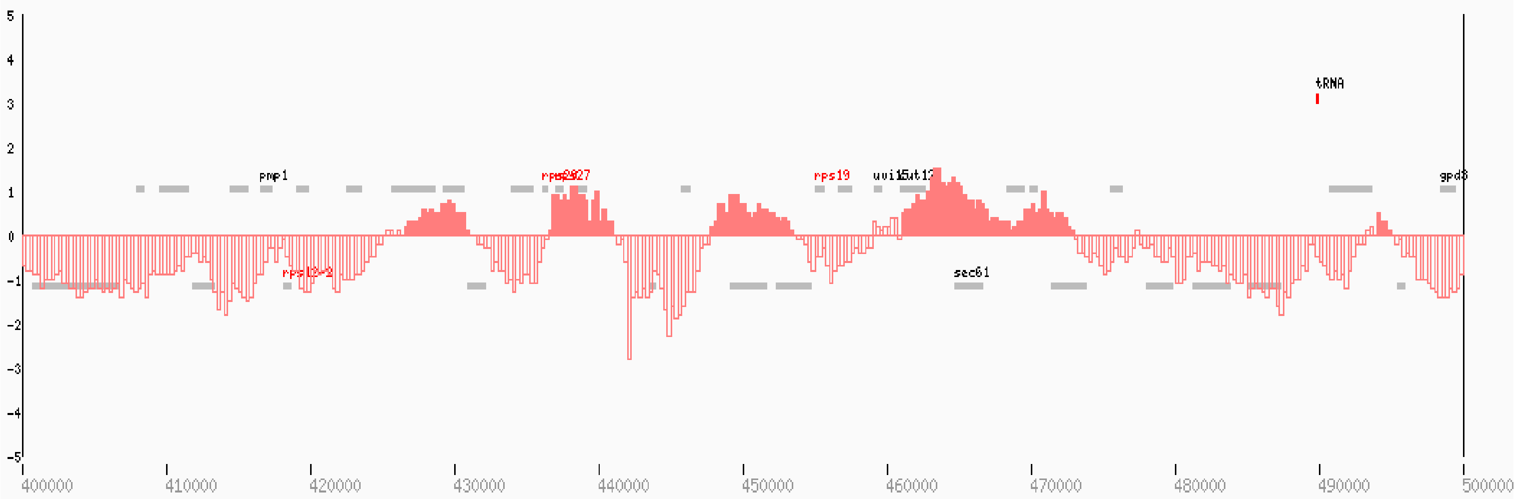


Chromosome 2 (500000-600000)

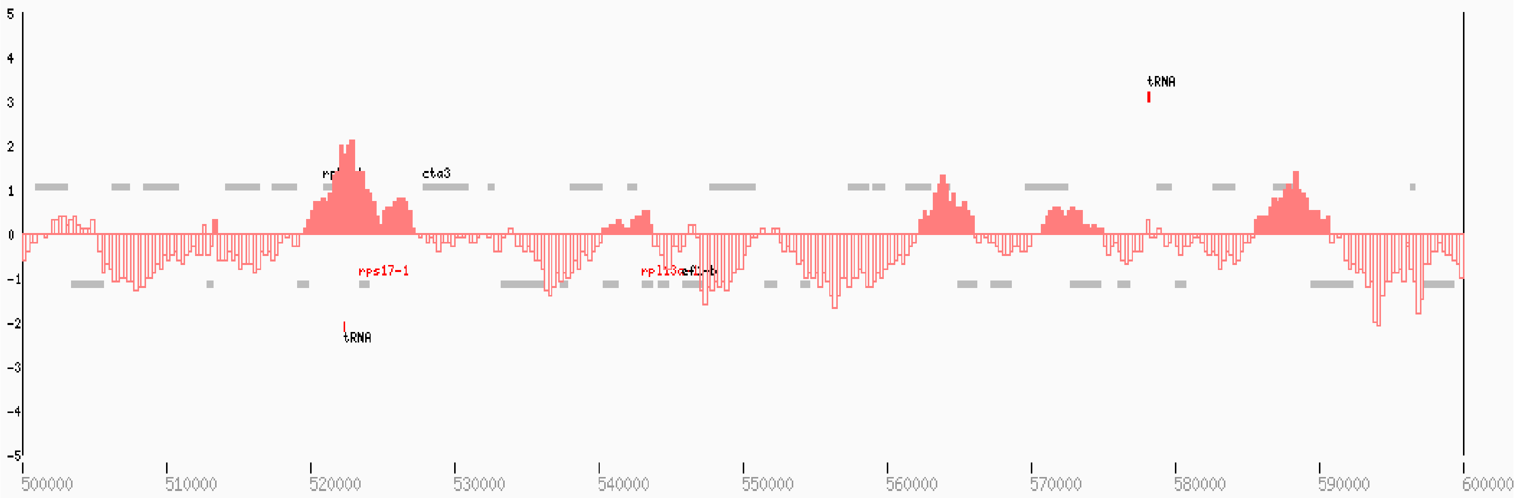


Chromosome 2 (600000-700000)

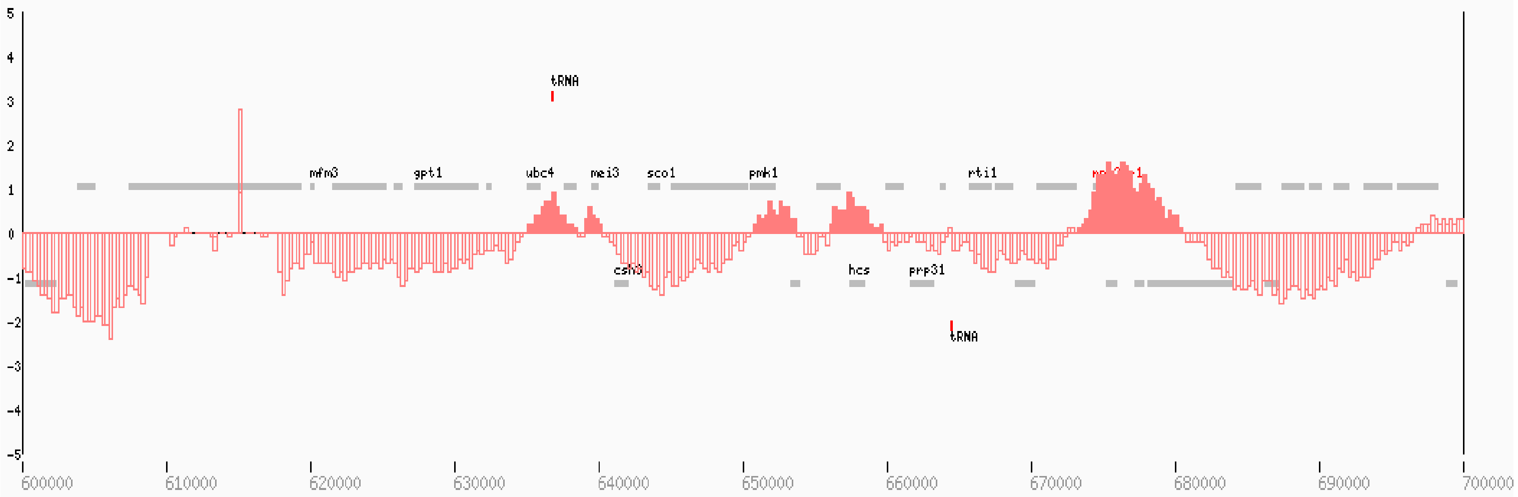


Chromosome 2 (700000-800000)

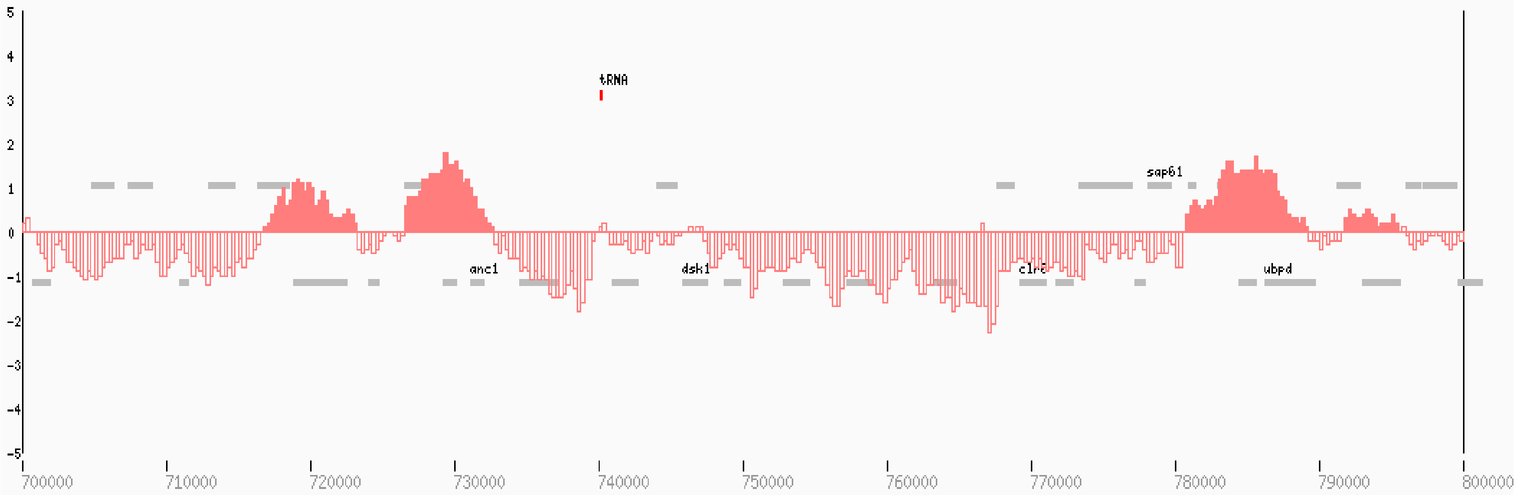

Chromosome 2 (800000-900000)

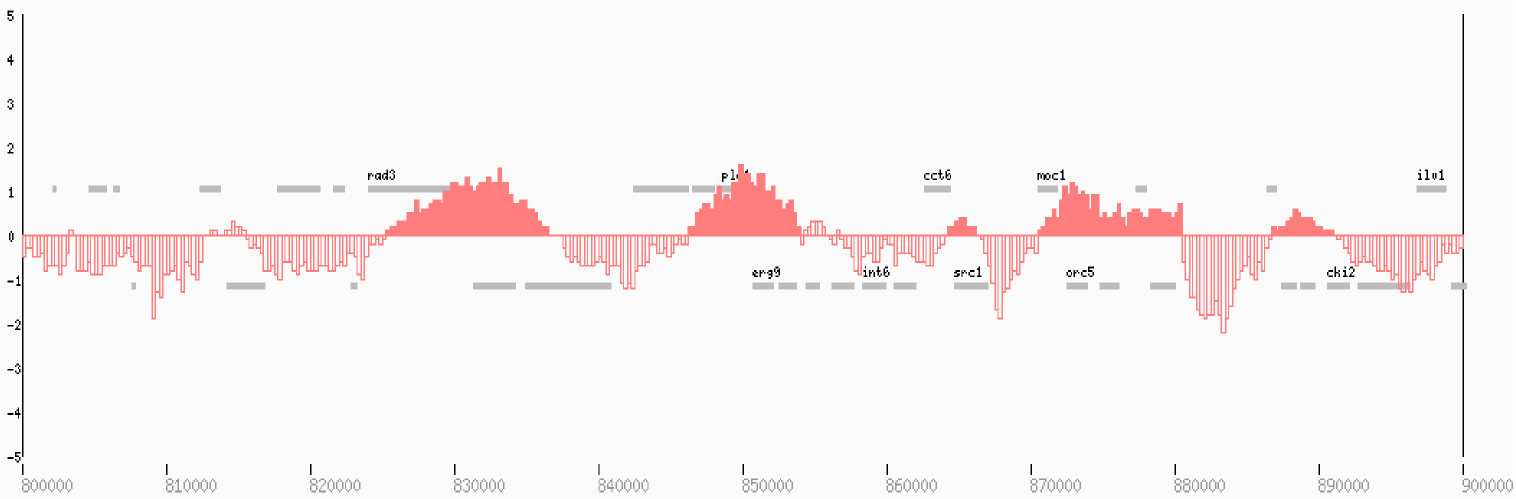


Chromosome 2 (900000-1000000)

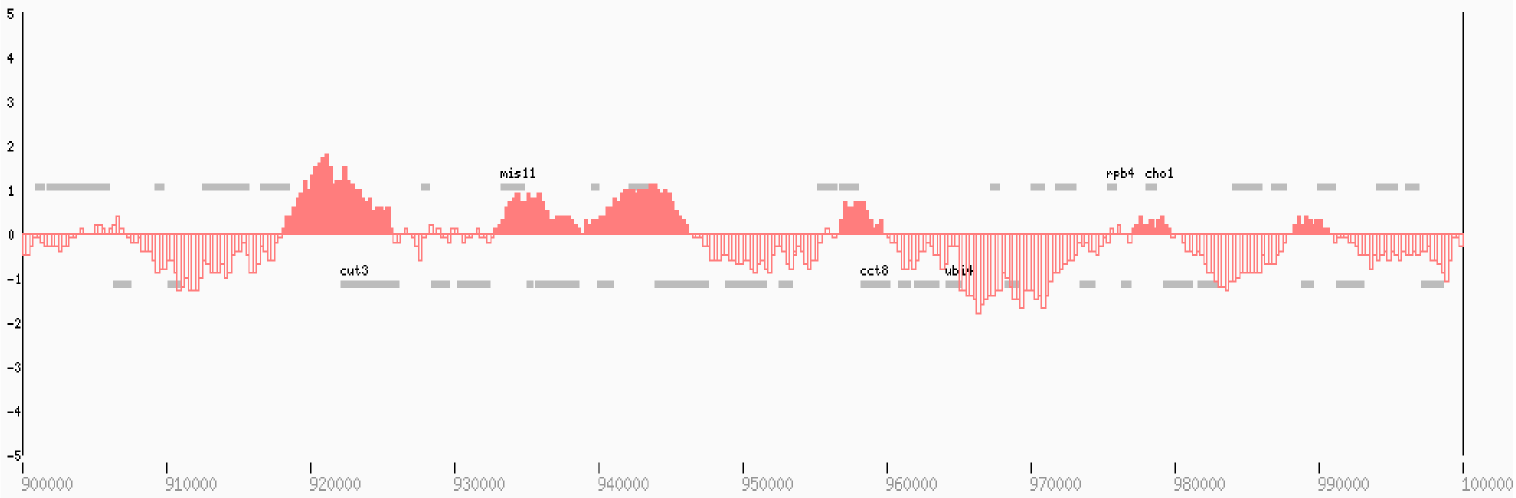


Chromosome 2 (1000000-1100000)

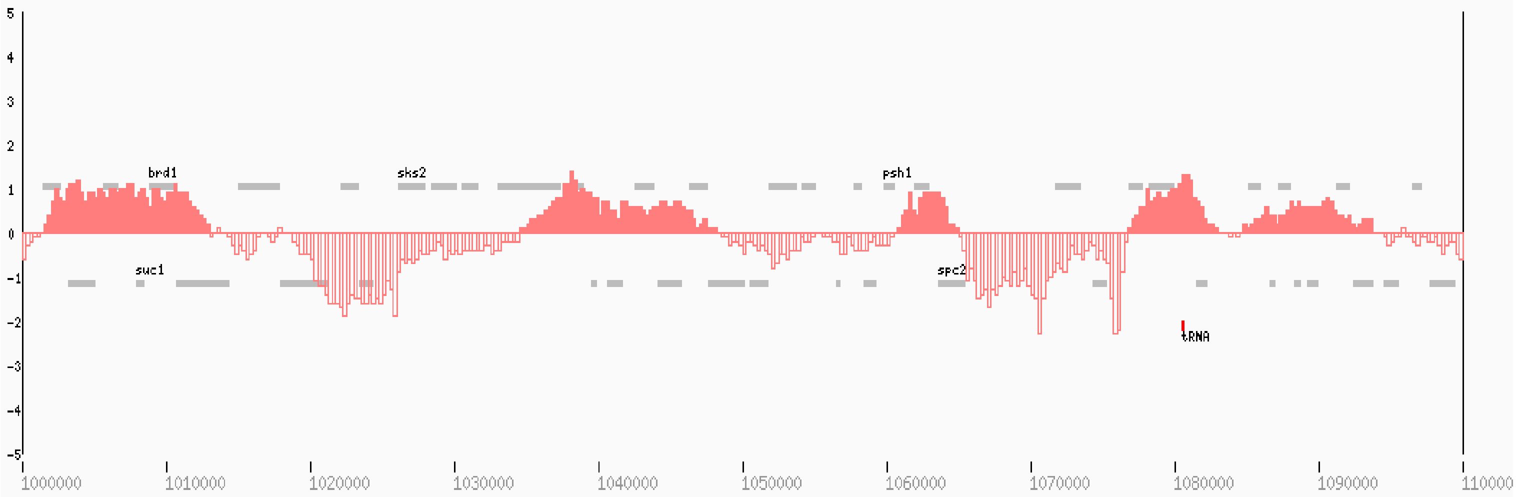


Chromosome 2 (1100000-1200000)

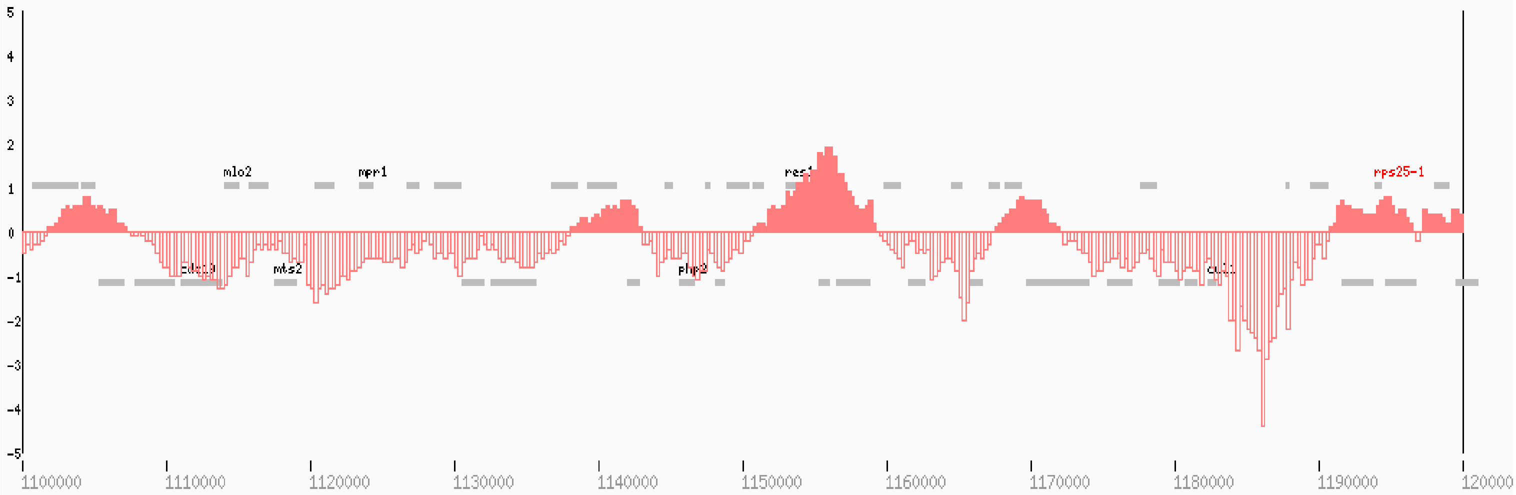

Chromosome 2 (1200000-1300000)

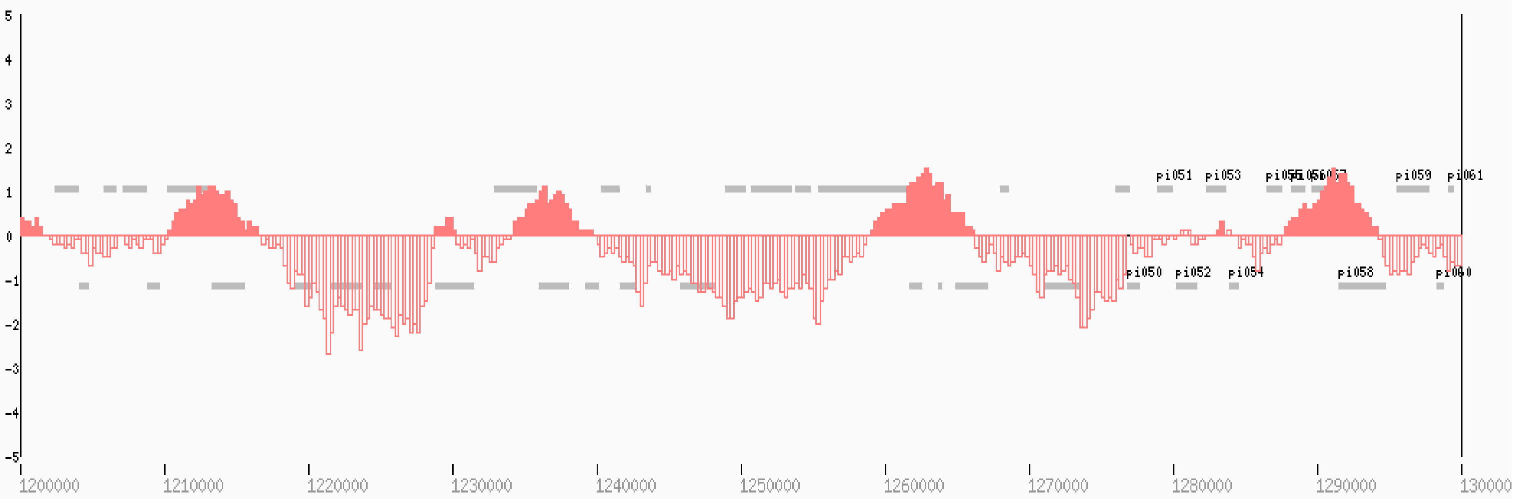


Chromosome 2 (1300000-1400000)

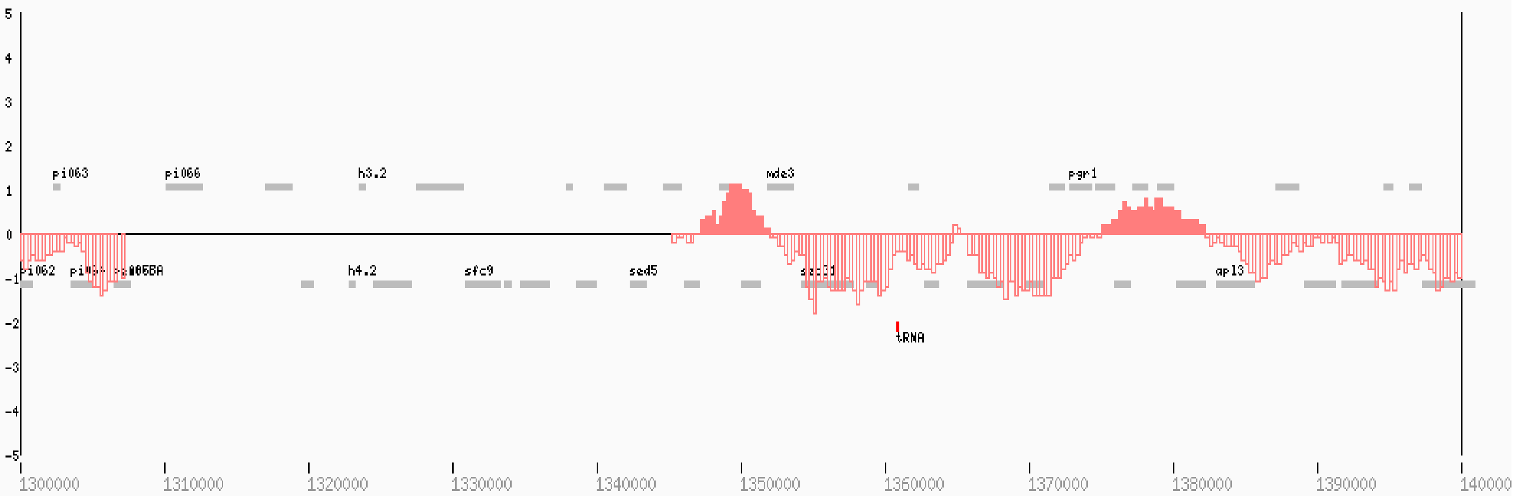


Chromosome 2 (1400000-1500000)

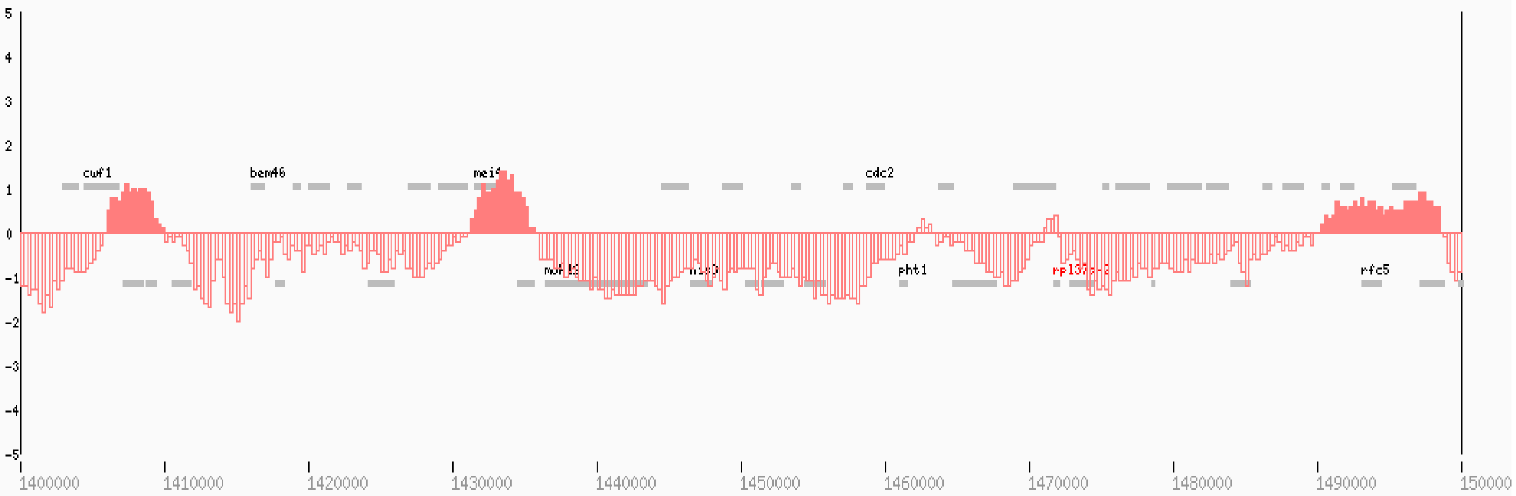


Chromosome 2 (1500000-1600000)

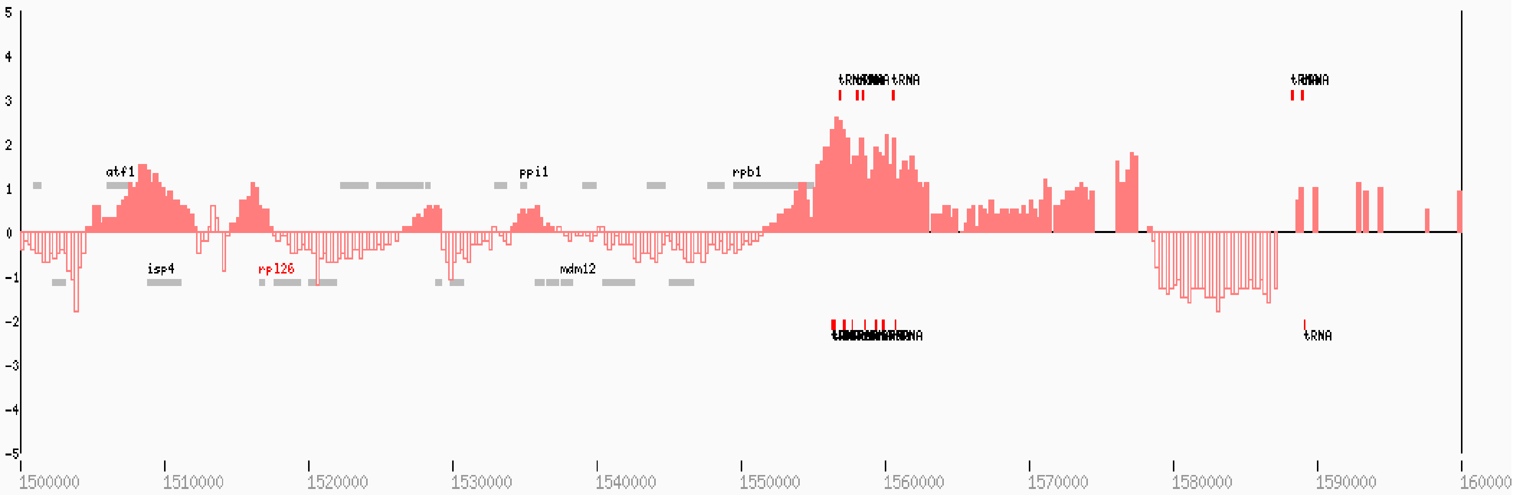

Chromosome 2 (1600000-1700000)

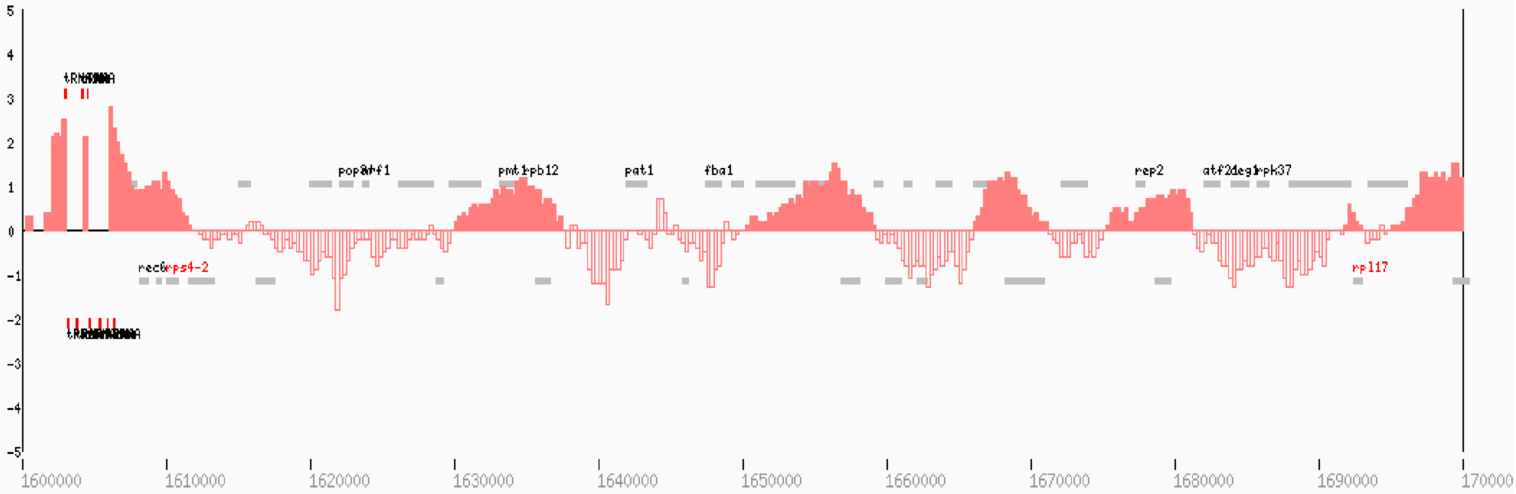


Chromosome 2 (1700000-1800000)

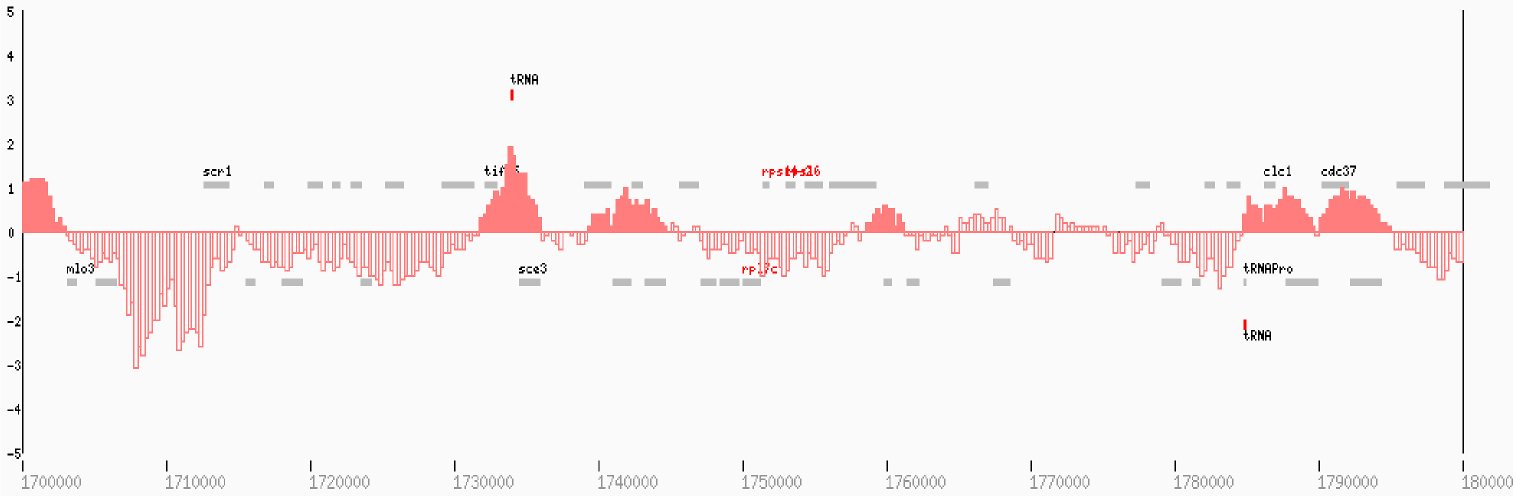


Chromosome 2 (1800000-1900000)

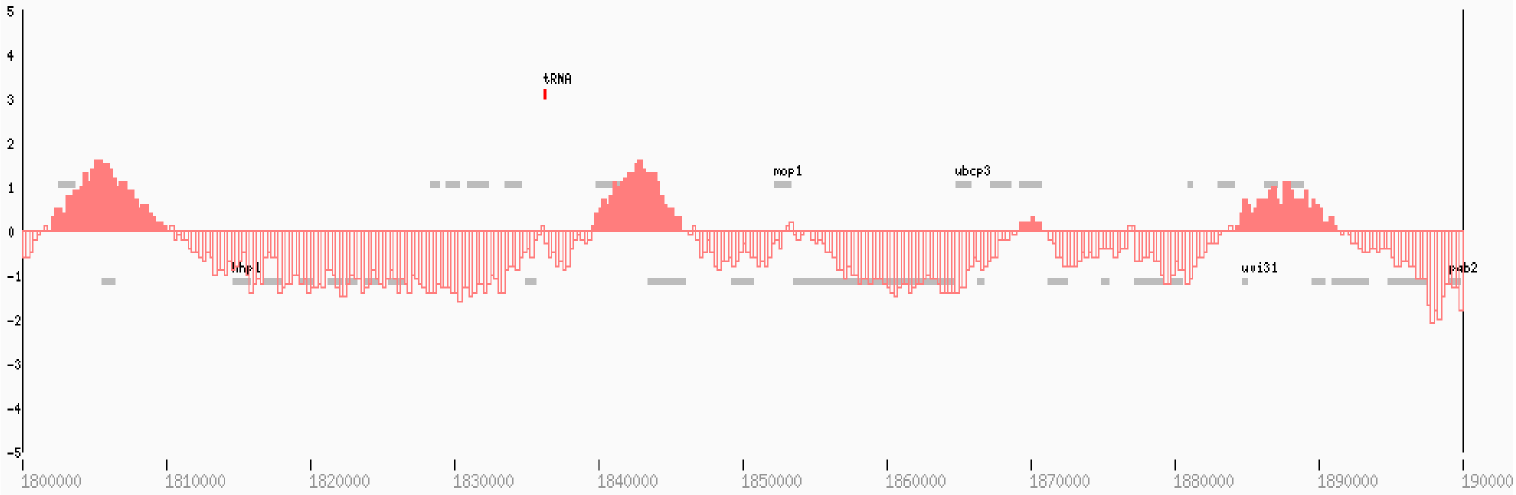


Chromosome 2 (1900000-2000000)

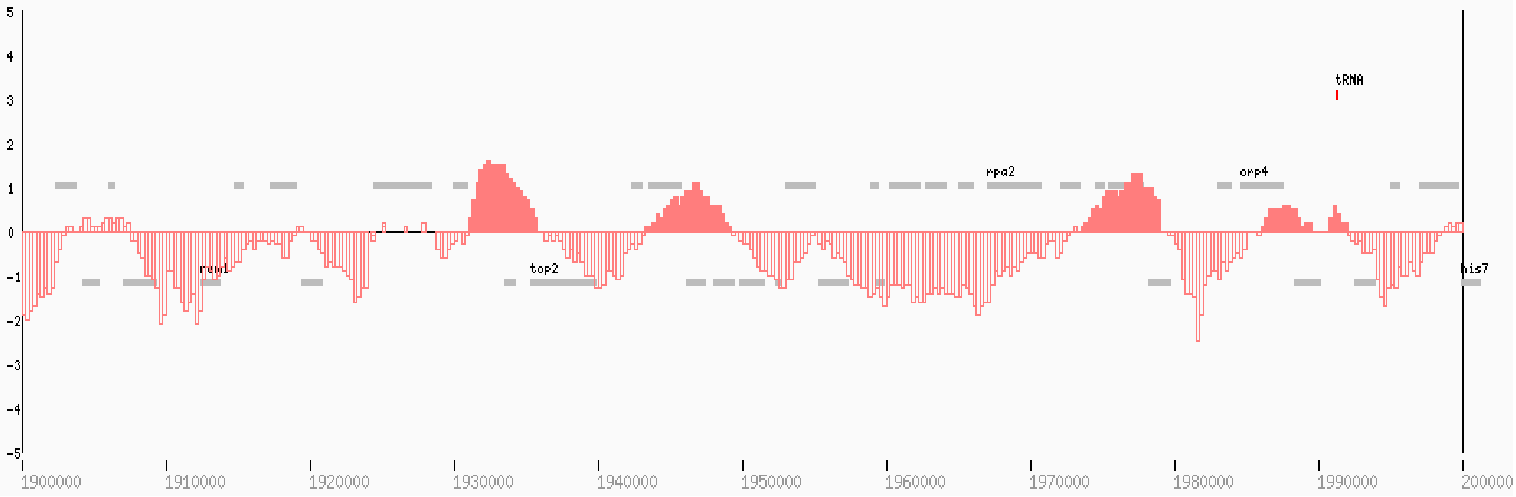

Chromosome 2 (2000000-2100000)

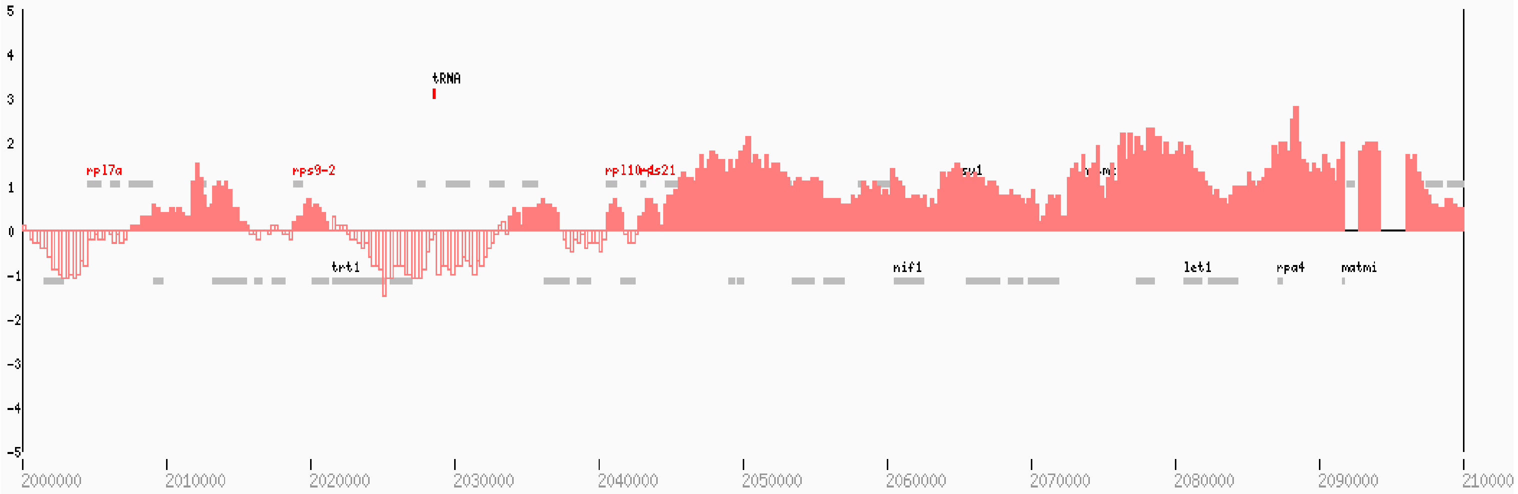


Chromosome 2 (2100000-2200000)

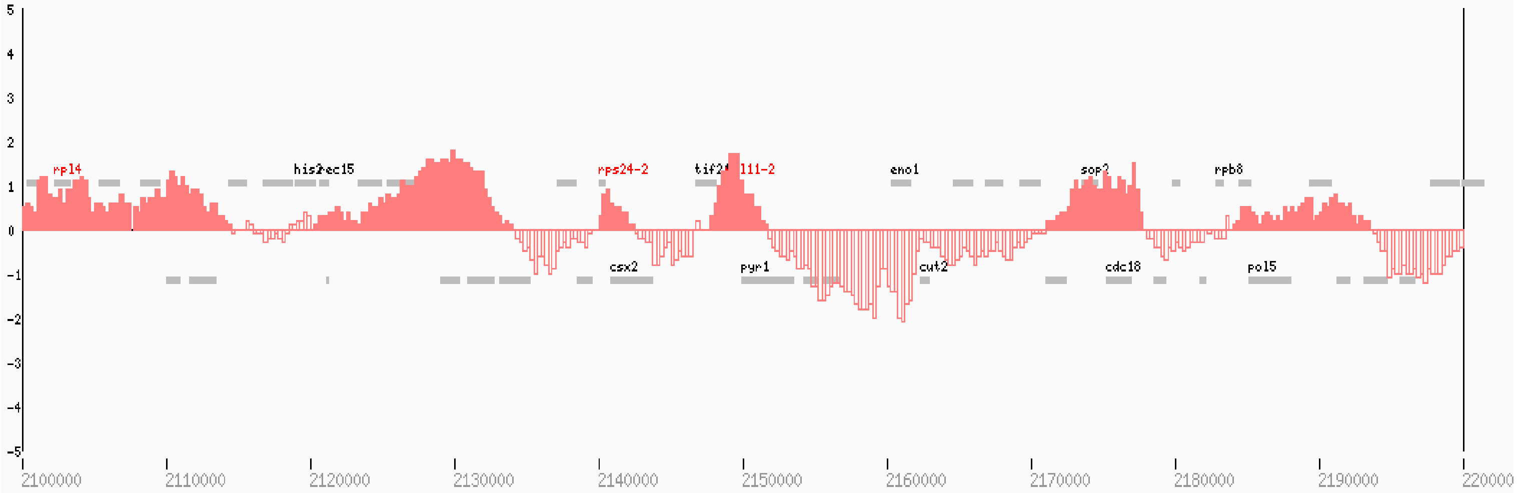


Chromosome 2 (2200000-2300000)

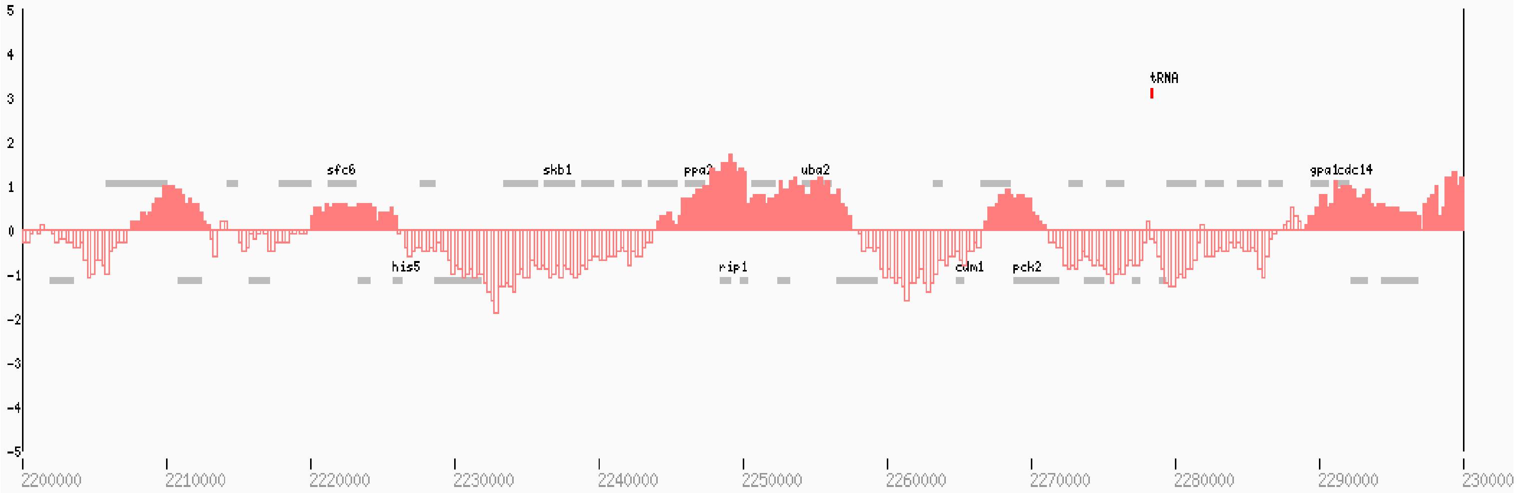


Chromosome 2 (2300000-2400000)

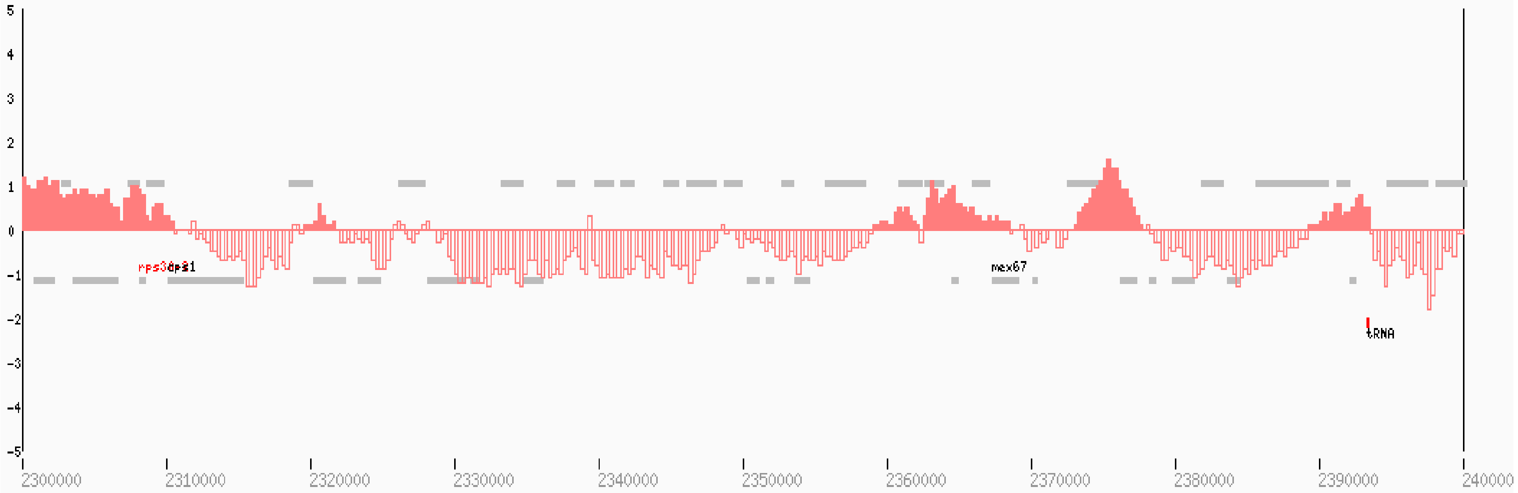

Chromosome 2 (2400000-2500000)

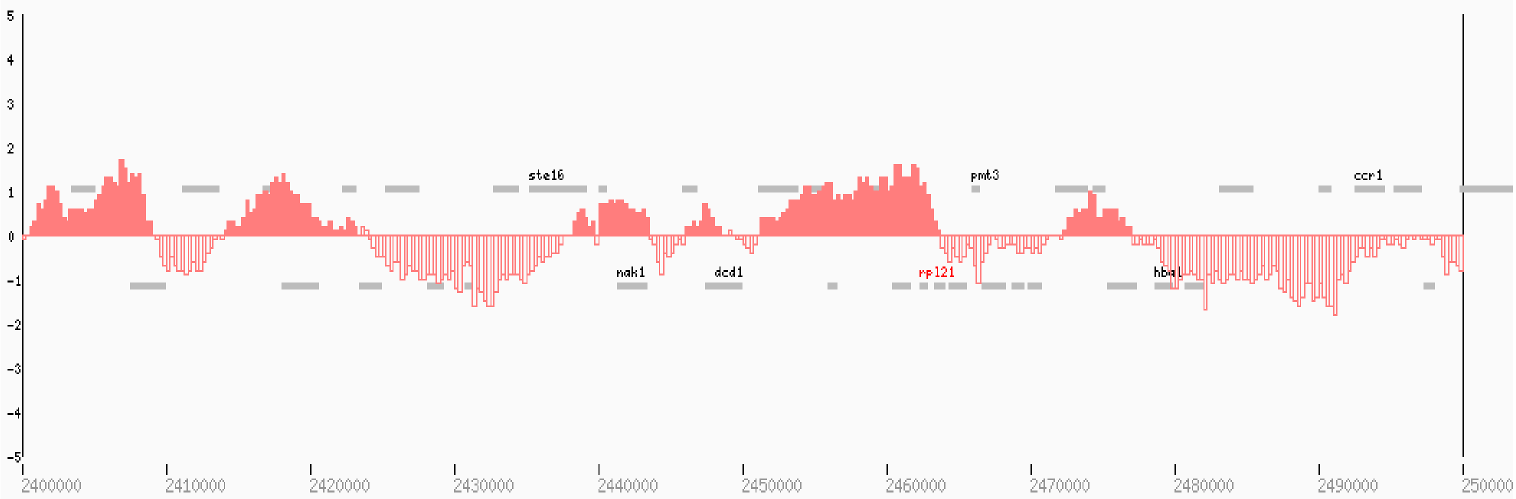


Chromosome 2 (2500000-2600000)

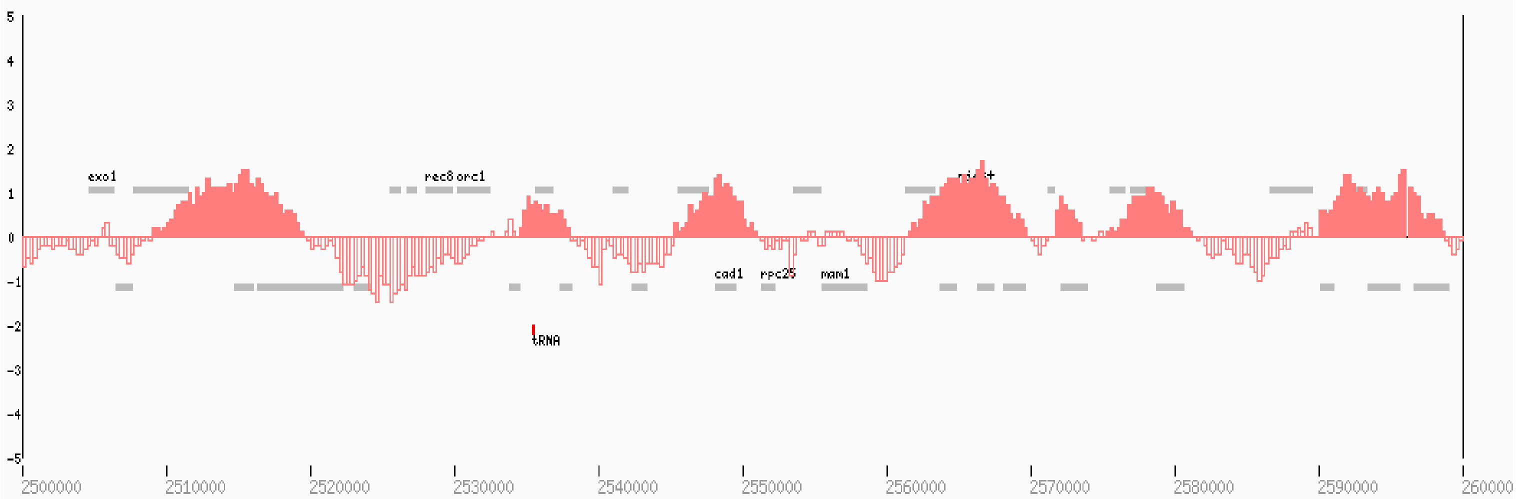


Chromosome 2 (2600000-2700000)

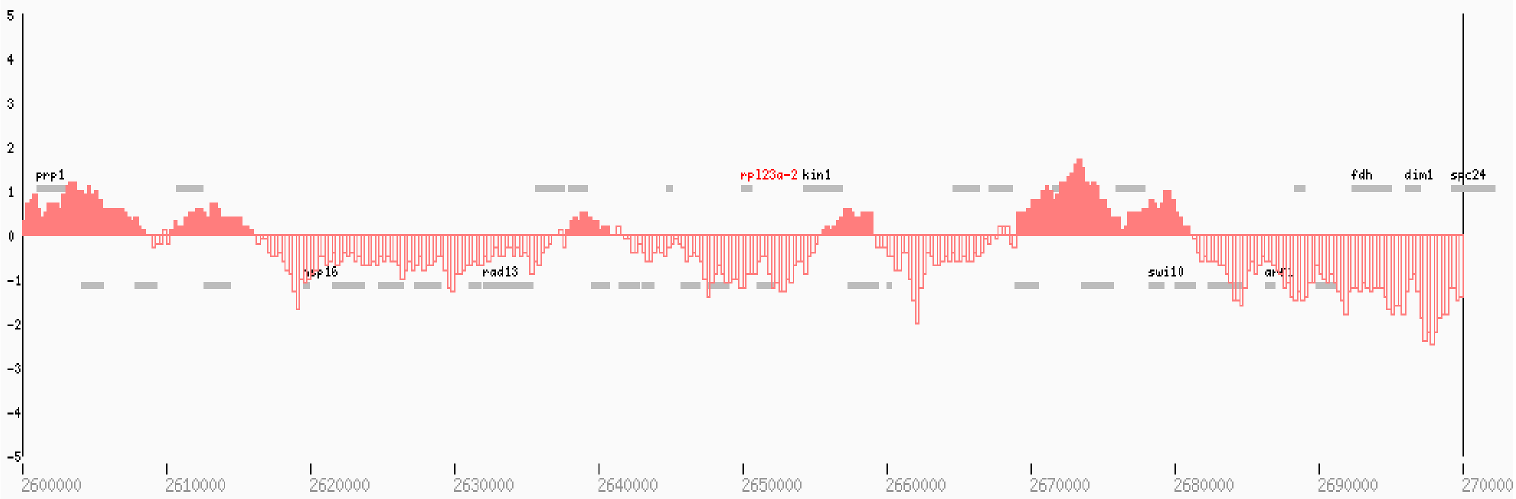


Chromosome 2 (2700000-2800000)

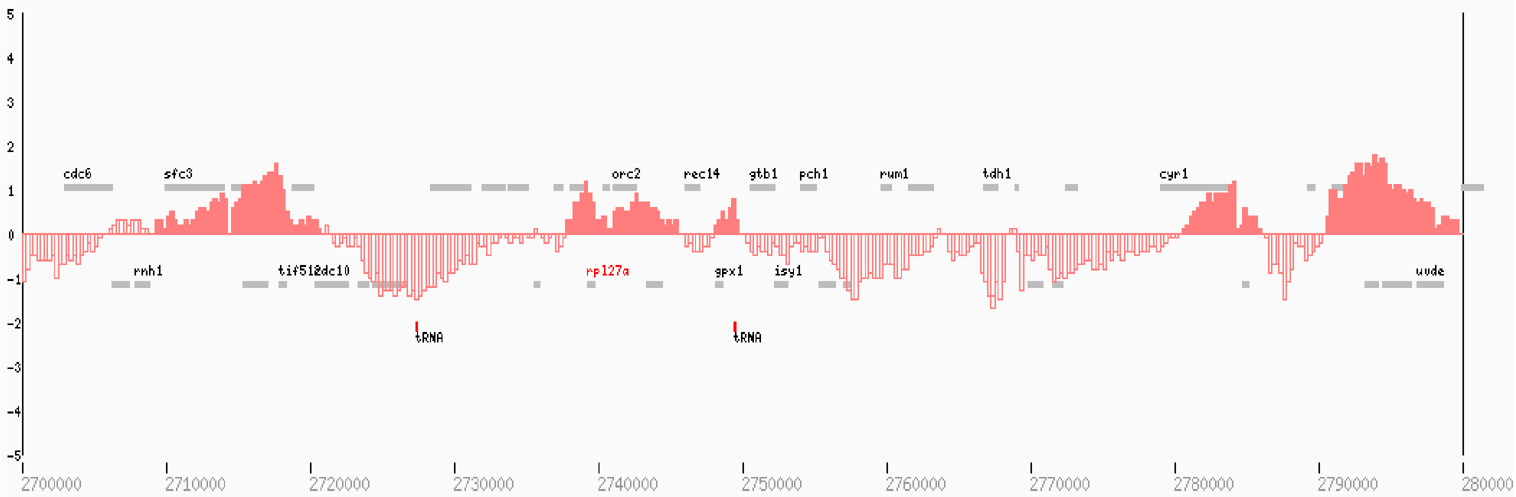

Chromosome 2 (2800000-2900000)

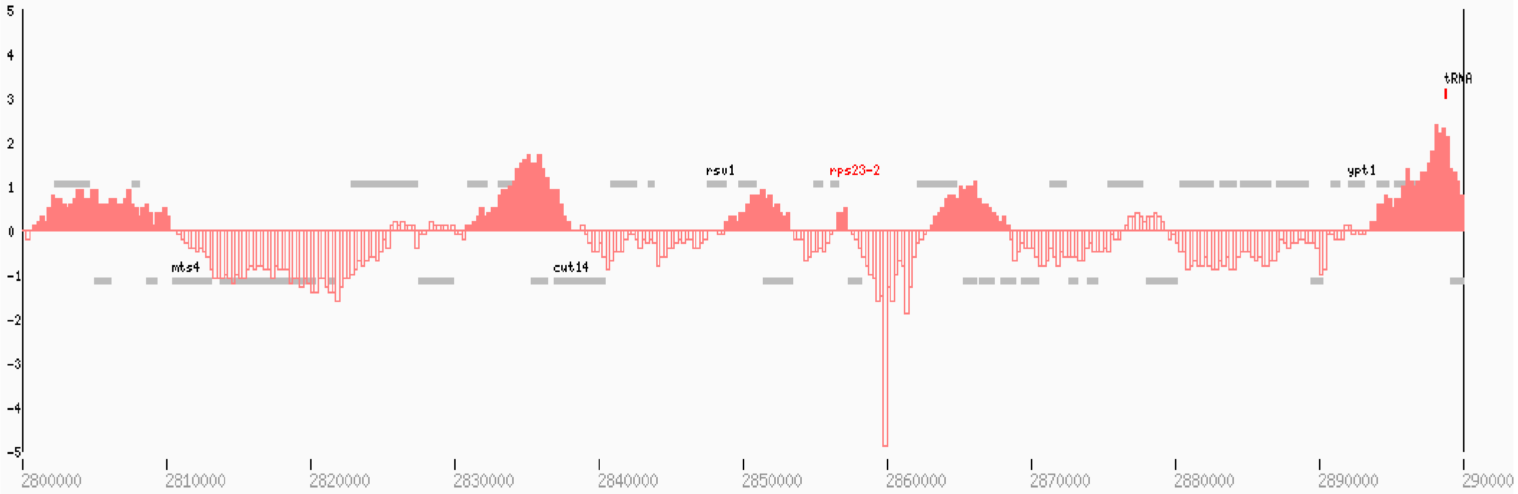


Chromosome 2 (2900000-3000000)

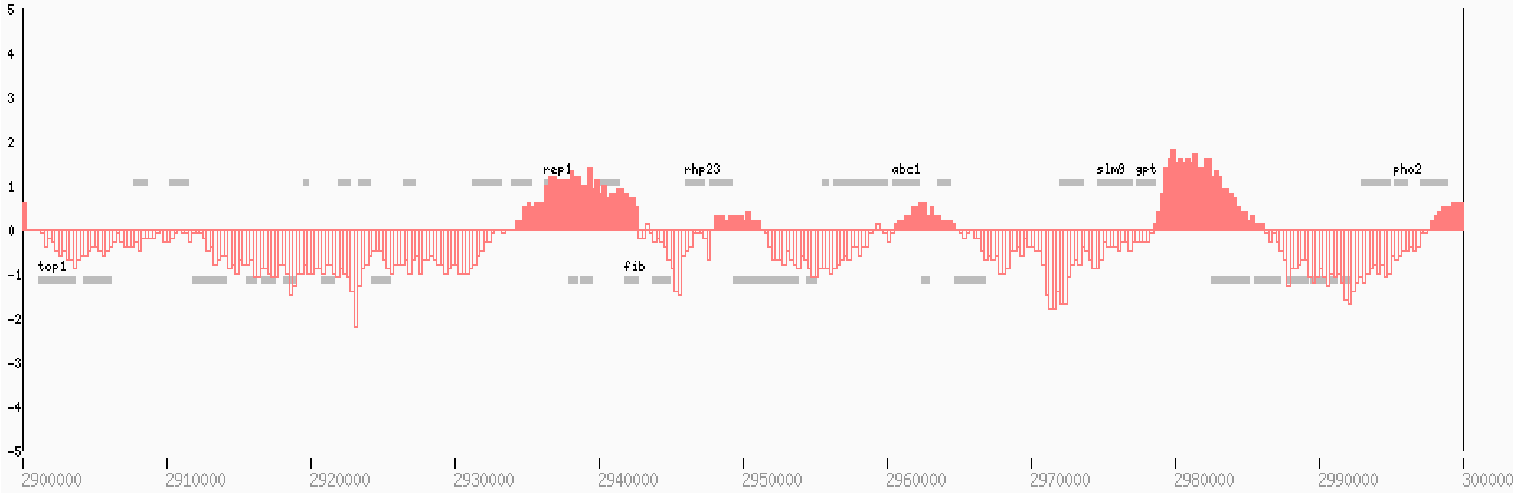


Chromosome 2 (3000000-3100000)

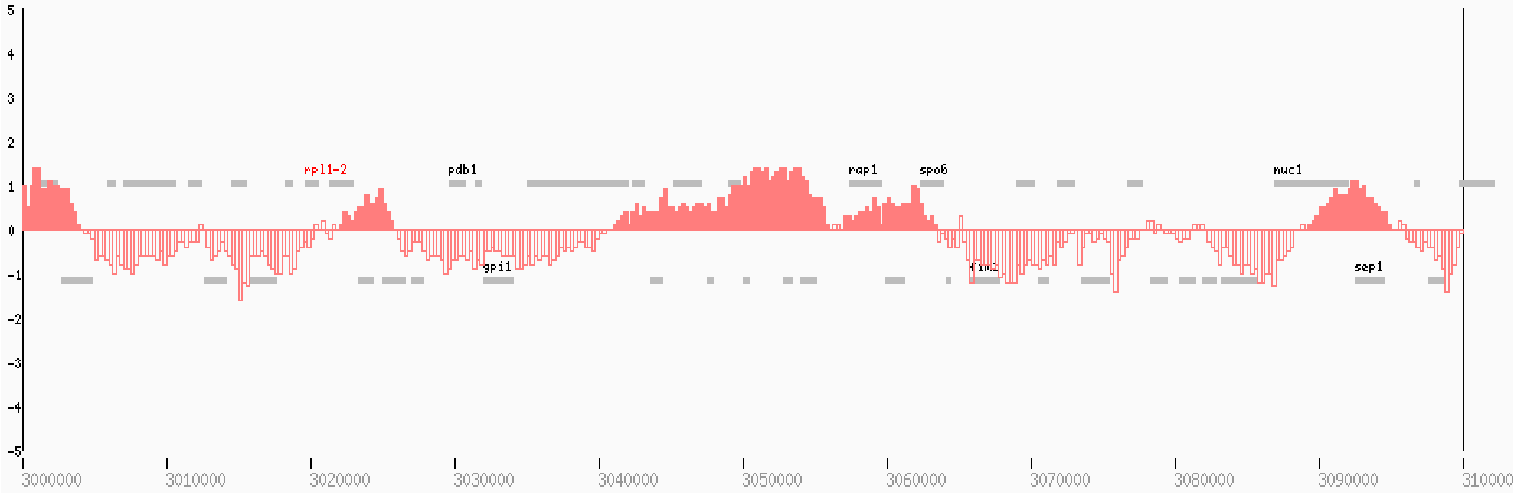


Chromosome 2 (3100000-3200000)

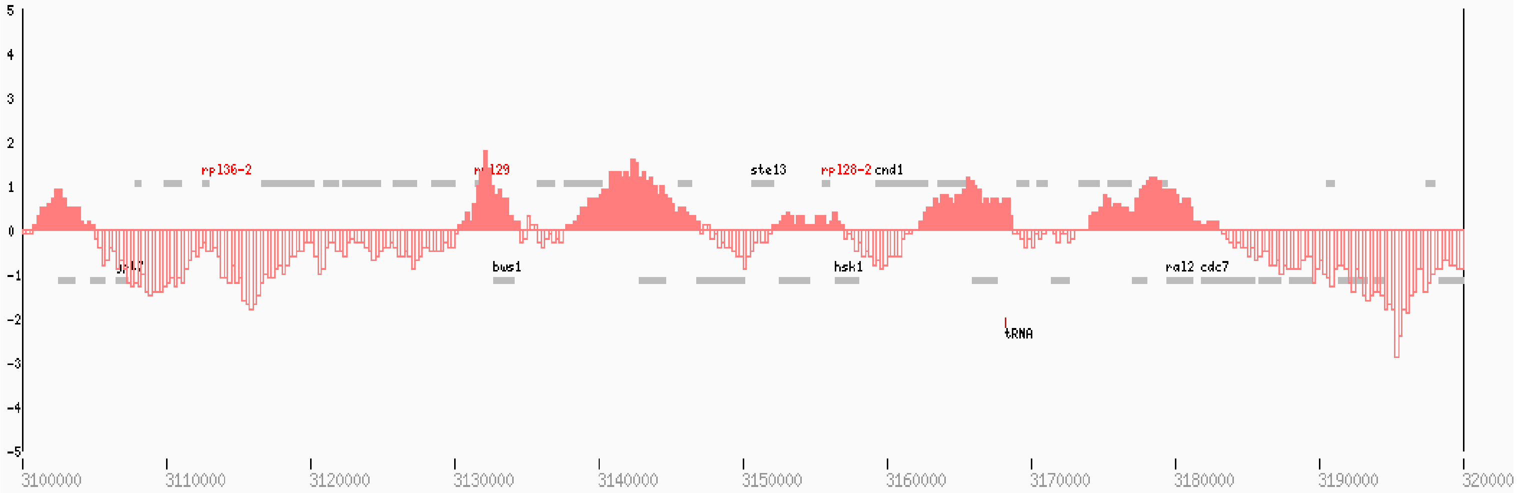

Chromosome 2 (3200000-3300000)

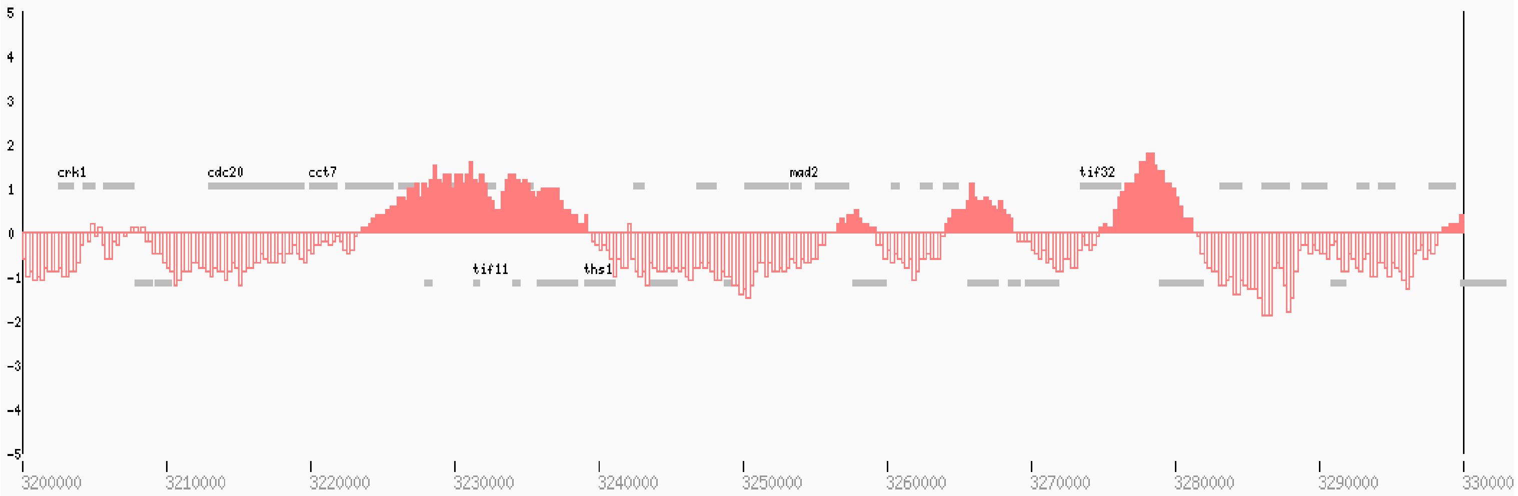


Chromosome 2 (3300000-3400000)

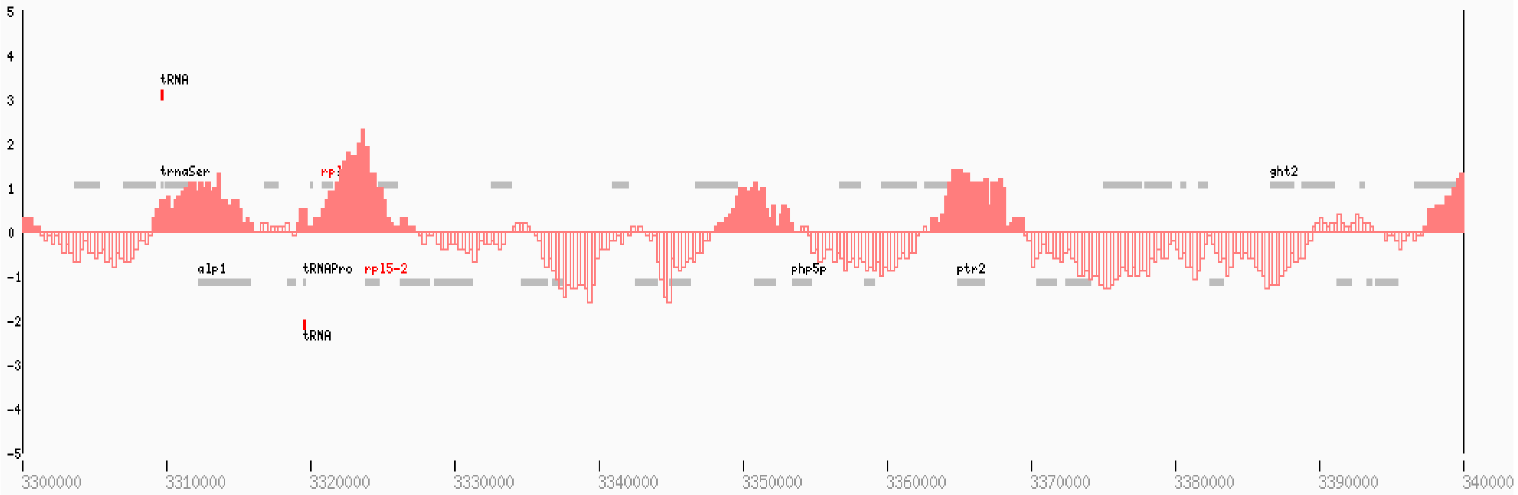


Chromosome 2 (3400000-3500000)

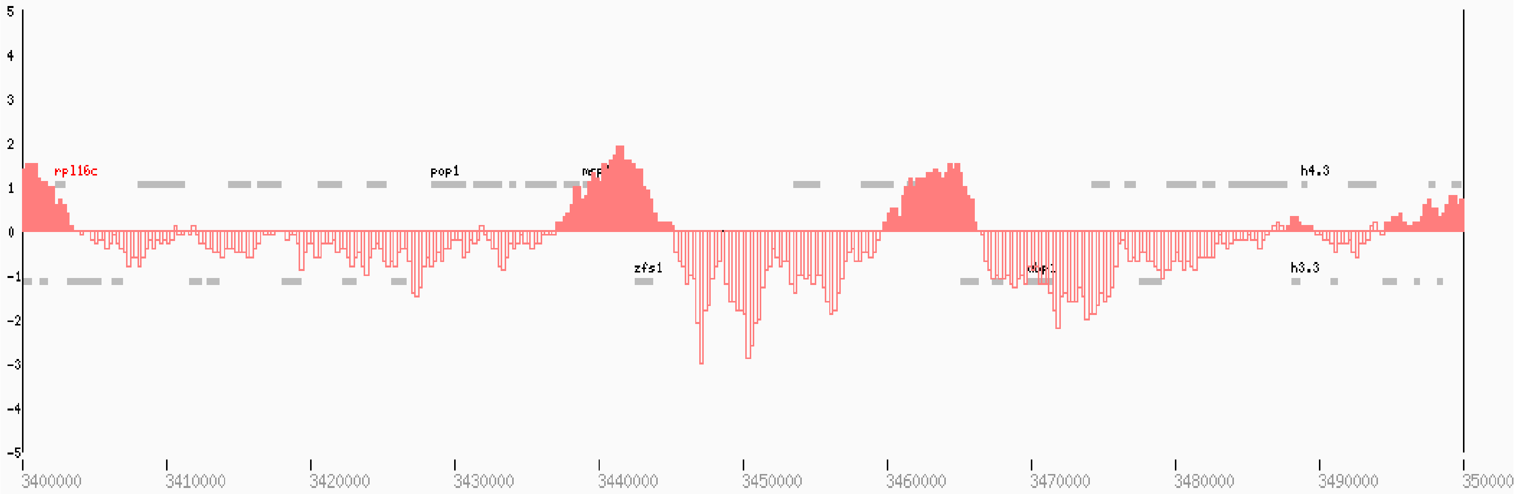


Chromosome 2 (3500000-3600000)

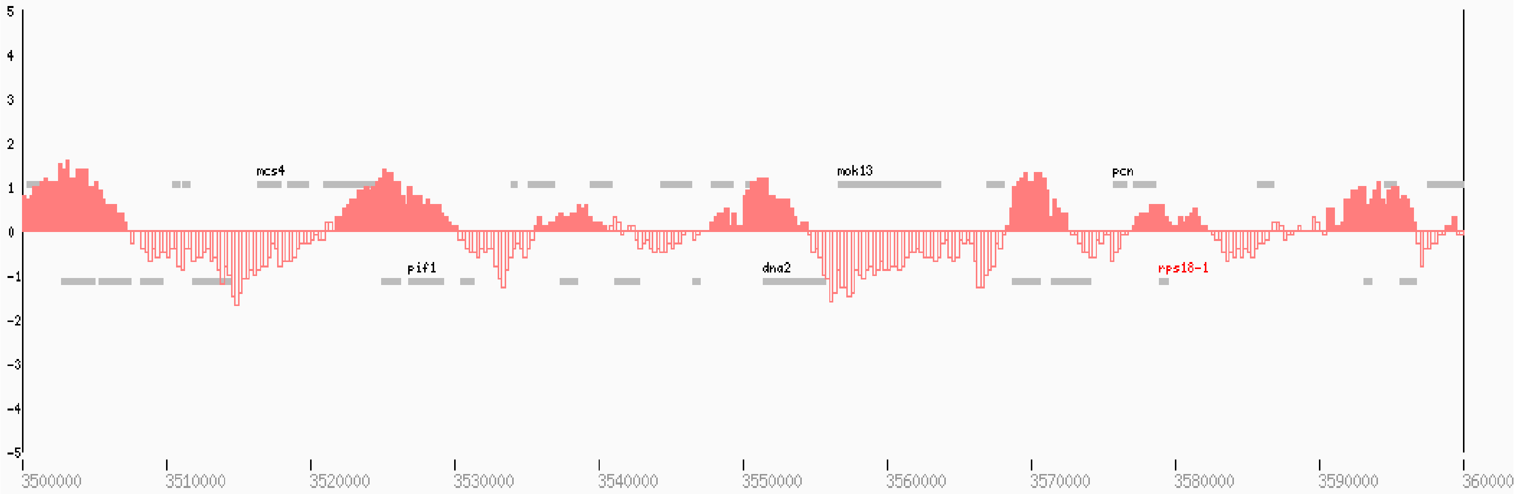

Chromosome 2 (3600000-3700000)

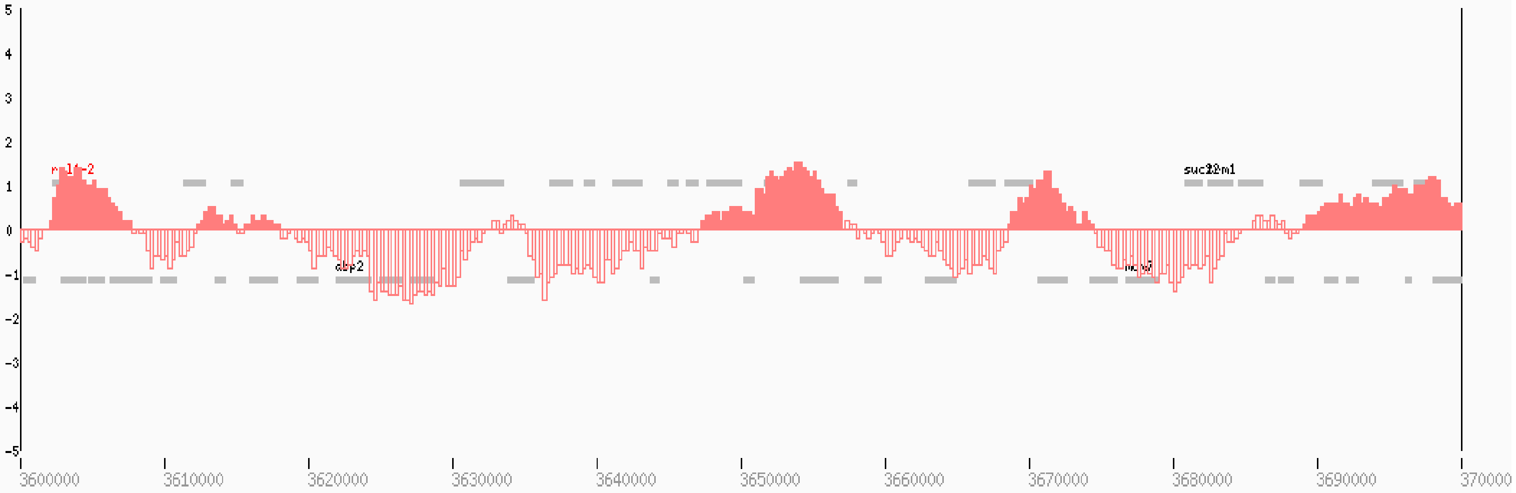


Chromosome 2 (3700000-3800000)

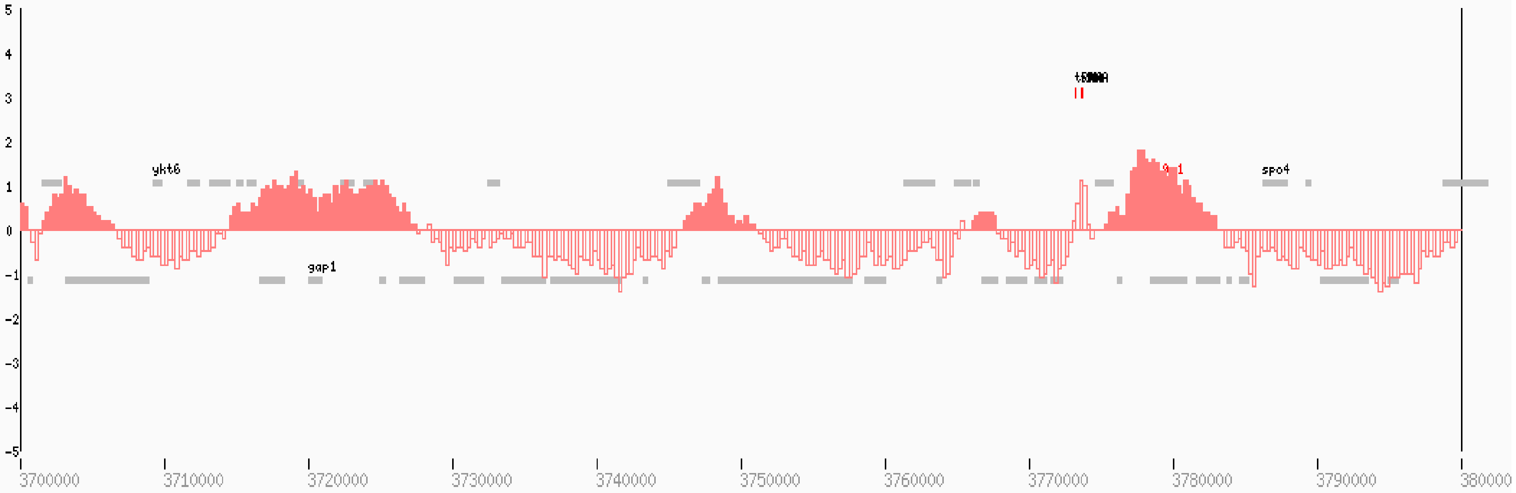


Chromosome 2 (3800000-3900000)

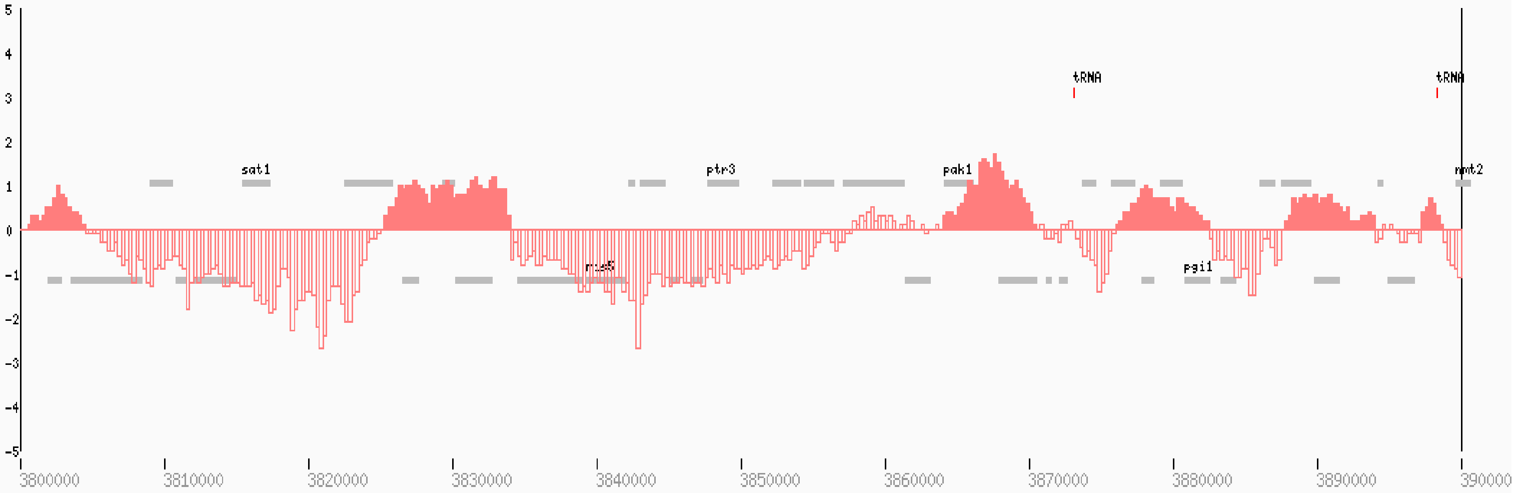


Chromosome 2 (3900000-4000000)

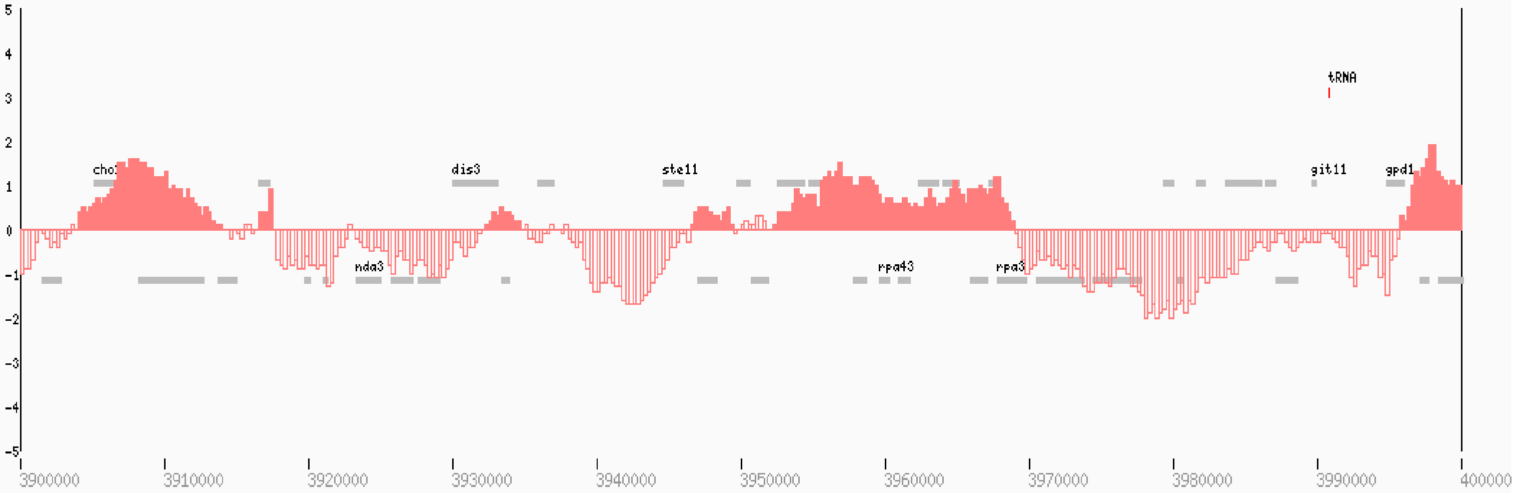

Chromosome 2 (4000000-4100000)

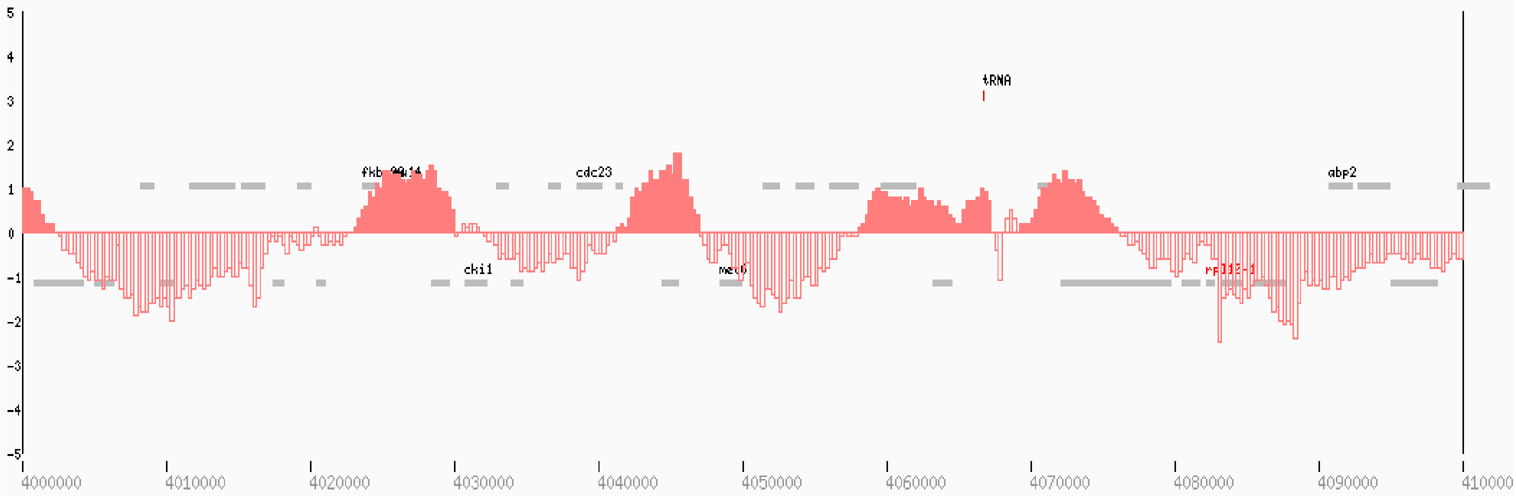


Chromosome 2 (4100000-4200000)

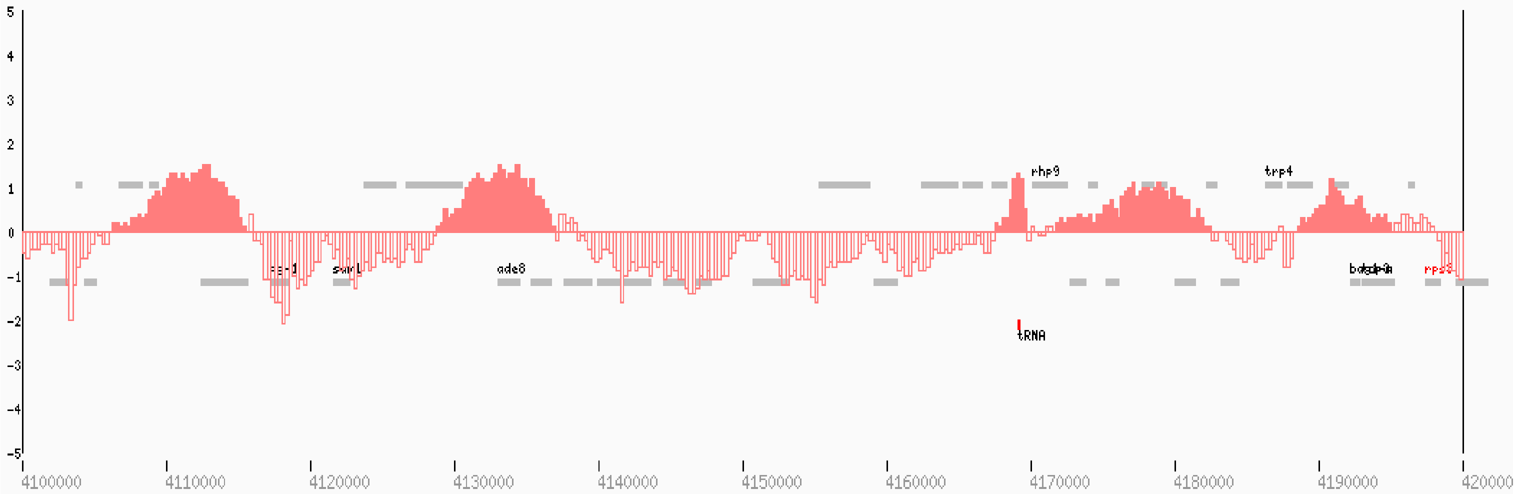


Chromosome 2 (4200000-4300000)

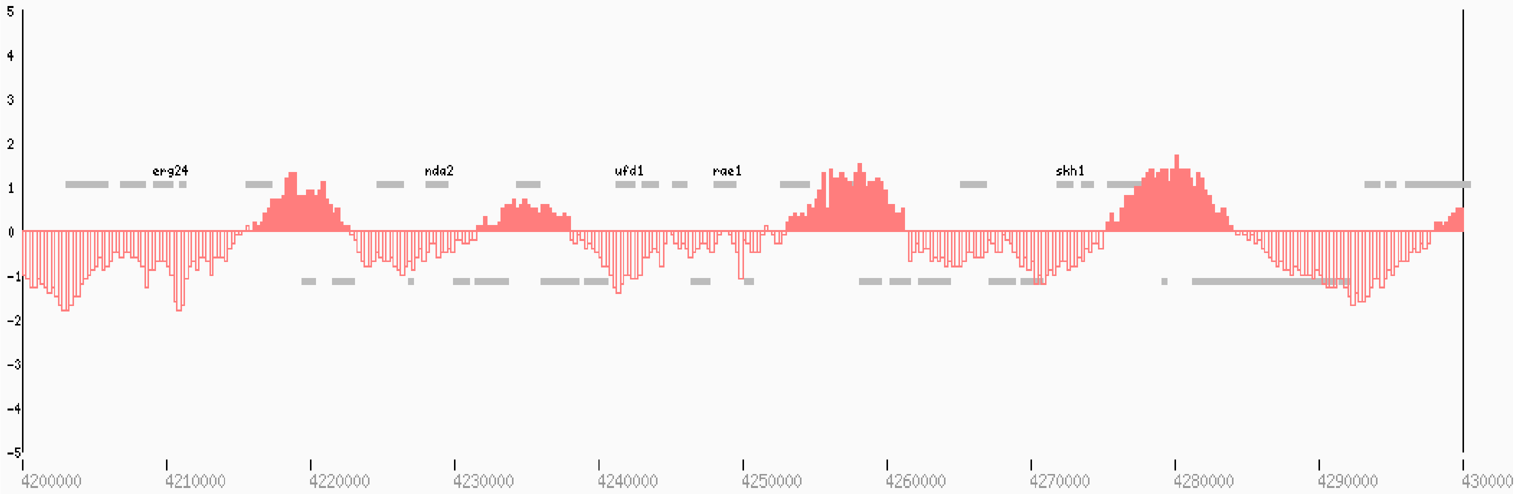


Chromosome 2 (4300000-4400000)

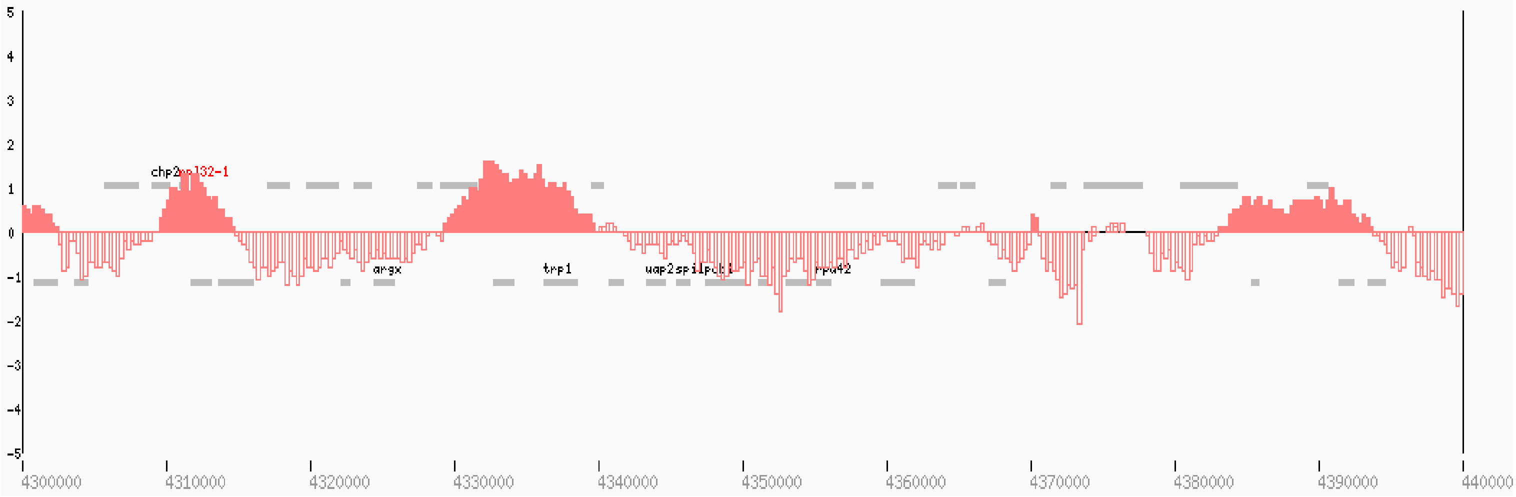

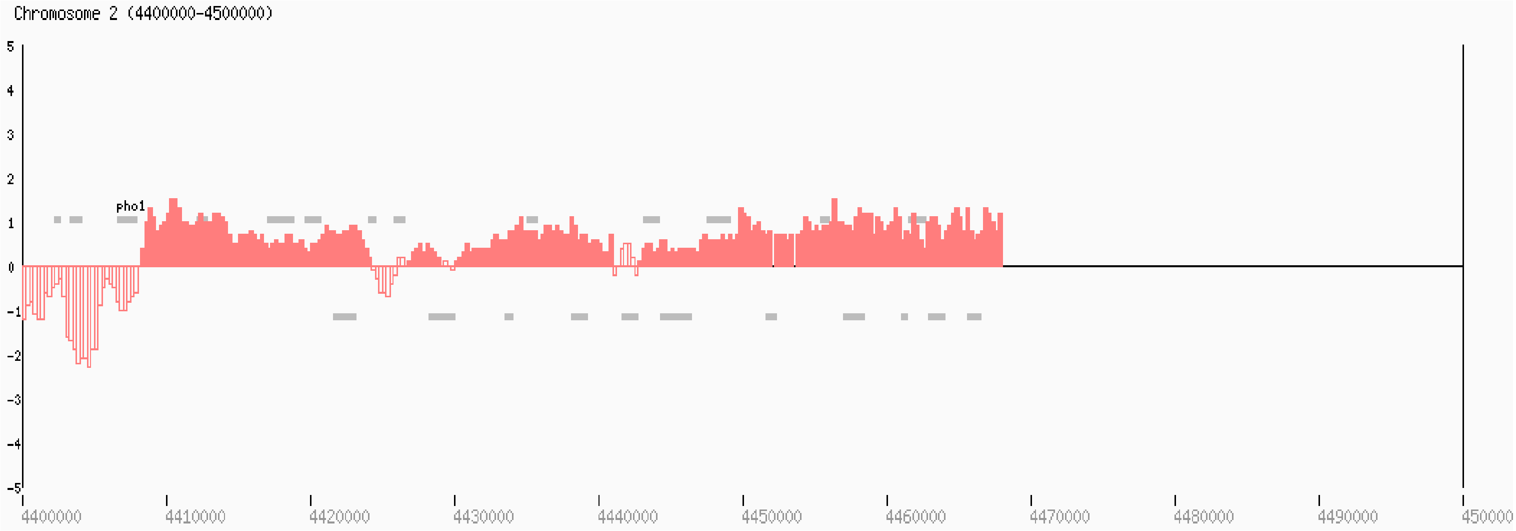

Chromosome 2 (4400000-4500000)
5
4
3
2
1
0
-1
-2
-3
-4
-5
pho1
4400000
4410000
4420000
4430000
4440000
4450000
4460000
4470000
4480000
4490000
450000

**(b)**

| Peak No | Midpoint/bp | Avg height | Width/bp |
|---|---|---|---|
| 1 | 7251 | 0,74 | 13000 |
| 2 | 28251 | 0,76 | 6000 |
| 3 | 57751 | 0,62 | 3500 |
| 4 | 79751 | 1,11 | 9000 |
| 5 | 137876 | 0,57 | 5750 |
| 6 | 154376 | 0,85 | 6250 |
| 7 | 177501 | 1,19 | 4000 |
| 8 | 189751 | 0,63 | 5000 |
| 9 | 204001 | 0,84 | 5000 |
| 10 | 213501 | 0,30 | 6000 |
| 11 | 237126 | 0,42 | 6250 |
| 12 | 246126 | 0,78 | 1750 |
| 13 | 256751 | 0,95 | 5500 |
| 14 | 270501 | 0,46 | 5500 |
| 15 | 286876 | 0,51 | 4750 |
| 16 | 317876 | 0,80 | 7250 |
| 17 | 343751 | 0,93 | 6500 |
| 18 | 356376 | 0,66 | 6750 |
| 19 | 390376 | 0,63 | 10250 |
| 20 | 428626 | 0,53 | 4250 |
| 21 | 438626 | 0,78 | 4250 |
| 22 | 450501 | 0,57 | 5500 |
| 23 | 466876 | 0,70 | 11750 |
| 24 | 494376 | 0,30 | 750 |
| 25 | 523251 | 0,96 | 7500 |
| 26 | 541876 | 0,28 | 3250 |
| 27 | 564001 | 0,75 | 3500 |
| 28 | 572751 | 0,39 | 4000 |
| 29 | 588001 | 0,71 | 5000 |
| 30 | 636626 | 0,48 | 3250 |
| 31 | 639501 | 0,43 | 1000 |
| 32 | 652126 | 0,49 | 2750 |
| 33 | 657751 | 0,50 | 3500 |
| 34 | 676751 | 0,97 | 7000 |
| 35 | 719876 | 0,68 | 6250 |
| 36 | 729501 | 1,06 | 6000 |
| 37 | 784876 | 0,98 | 8250 |
| 38 | 793501 | 0,32 | 3500 |
| 39 | 830751 | 0,81 | 11000 |
| 40 | 850001 | 0,93 | 7500 |
| 41 | 865126 | 0,28 | 1750 |
| 42 | 875376 | 0,61 | 9750 |
| 43 | 888751 | 0,29 | 4000 |

| Peak No | Midpoint/bp | Avg height | Width/bp |
|---|---|---|---|
| 115 | 2320501 | 0,21 | 2000 |
| 116 | 2363626 | 0,41 | 9250 |
| 117 | 2375251 | 0,91 | 4500 |
| 118 | 2391251 | 0,41 | 4000 |
| 119 | 2404626 | 0,89 | 8250 |
| 120 | 2418501 | 0,61 | 9000 |
| 121 | 2440751 | 0,55 | 5000 |
| 122 | 2447126 | 0,36 | 2250 |
| 123 | 2457376 | 0,98 | 12250 |
| 124 | 2474501 | 0,54 | 4500 |
| 125 | 2514126 | 0,88 | 10250 |
| 126 | 2536126 | 0,63 | 3250 |
| 127 | 2548001 | 0,74 | 5500 |
| 128 | 2565501 | 0,96 | 8000 |
| 129 | 2572501 | 0,64 | 1500 |
| 130 | 2578126 | 0,65 | 5750 |
| 131 | 2594251 | 0,89 | 8500 |
| 132 | 2604126 | 0,72 | 8250 |
| 133 | 2613001 | 0,43 | 5500 |
| 134 | 2639126 | 0,33 | 2750 |
| 135 | 2657126 | 0,39 | 3250 |
| 136 | 2674876 | 0,80 | 11750 |
| 137 | 2714876 | 0,65 | 11250 |
| 138 | 2741501 | 0,54 | 7500 |
| 139 | 2748751 | 0,50 | 1500 |
| 140 | 2783126 | 0,63 | 5250 |
| 141 | 2795001 | 1,00 | 9000 |
| 142 | 2805376 | 0,59 | 9250 |
| 143 | 2834251 | 0,91 | 7000 |
| 144 | 2850876 | 0,55 | 4250 |
| 145 | 2856751 | 0,40 | 500 |
| 146 | 2865626 | 0,64 | 5250 |
| 147 | 2896751 | 1,20 | 6500 |
| 148 | 2938376 | 0,89 | 8250 |
| 149 | 2949501 | 0,27 | 3000 |
| 150 | 2962501 | 0,35 | 4000 |
| 151 | 2982251 | 1,05 | 7500 |
| 152 | 3000751 | 0,78 | 6000 |
| 153 | 3023751 | 0,53 | 3500 |
| 154 | 3048376 | 0,78 | 14750 |
| 155 | 3060126 | 0,49 | 6250 |
| 156 | 3092001 | 0,69 | 5500 |
| 157 | 3102751 | 0,51 | 4000 |

| | | | | | | | |
|---|---|---|---|---|---|---|---|
| 44 | 921751 | 1,00 | 7500 | 158 | 3132251 | 0,77 | 4000 |
| 45 | 935626 | 0,55 | 5750 | 159 | 3142251 | 0,83 | 9000 |
| 46 | 942501 | 0,74 | 7000 | 160 | 3154376 | 0,23 | 4750 |
| 47 | 958126 | 0,51 | 2750 | 161 | 3165376 | 0,73 | 6250 |
| 48 | 978251 | 0,26 | 2500 | 162 | 3178376 | 0,63 | 8750 |
| 49 | 989376 | 0,28 | 2250 | 163 | 3231251 | 0,89 | 15500 |
| 50 | 1007126 | 0,86 | 11250 | 164 | 3257751 | 0,29 | 2500 |
| 51 | 1041251 | 0,59 | 13500 | 165 | 3266251 | 0,62 | 4500 |
| 52 | 1062751 | 0,65 | 4000 | 166 | 3277876 | 0,98 | 6250 |
| 53 | 1079751 | 0,76 | 6000 | 167 | 3299751 | 0,23 | 2500 |
| 54 | 1089126 | 0,44 | 8750 | 168 | 3312376 | 0,79 | 6750 |
| 55 | 1104376 | 0,48 | 5250 | 169 | 3323001 | 0,87 | 8000 |
| 56 | 1140376 | 0,44 | 4750 | 170 | 3350626 | 0,62 | 5250 |
| 57 | 1154876 | 0,95 | 8250 | 171 | 3366126 | 0,88 | 6250 |
| 58 | 1169626 | 0,51 | 4250 | 172 | 3400251 | 0,93 | 6000 |
| 59 | 1193501 | 0,52 | 5500 | 173 | 3441001 | 1,00 | 8000 |
| 60 | 1199251 | 0,35 | 4000 | 174 | 3462876 | 1,05 | 6250 |
| 61 | 1213251 | 0,70 | 6000 | 175 | 3488501 | 0,20 | 1500 |
| 62 | 1229376 | 0,30 | 1250 | 176 | 3500751 | 0,76 | 12500 |
| 63 | 1236876 | 0,61 | 5250 | 177 | 3525876 | 0,78 | 8250 |
| 64 | 1262501 | 0,81 | 7000 | 178 | 3537876 | 0,31 | 4750 |
| 65 | 1283126 | | 250 | 179 | 3551001 | 0,61 | 6500 |
| 66 | 1290876 | 0,81 | 6250 | 180 | 3570251 | 0,93 | 4000 |
| 67 | 1349501 | 0,65 | 4500 | 181 | 3579501 | 0,35 | 5000 |
| 68 | 1378501 | 0,48 | 7000 | 182 | 3593501 | 0,71 | 6000 |
| 69 | 1407876 | 0,80 | 3750 | 183 | 3599001 | 0,10 | 500 |
| 70 | 1433376 | 0,94 | 4250 | 184 | 3604751 | 0,91 | 5500 |
| 71 | 1494251 | 0,61 | 8000 | 185 | 3613501 | 0,31 | 2500 |
| 72 | 1508251 | 0,76 | 7500 | 186 | 3616626 | 0,20 | 2250 |
| 73 | 1515876 | 0,64 | 2750 | 187 | 3652001 | 0,84 | 9500 |
| 74 | 1527751 | 0,38 | 2500 | 188 | 3671376 | 0,67 | 5750 |
| 75 | 1535376 | 0,37 | 2750 | 189 | 3694626 | 0,71 | 11250 |
| 76 | 1558126 | 1,24 | 13250 | 190 | 3703876 | 0,61 | 4750 |
| 77 | 1571876 | 0,71 | 12750 | 191 | 3720876 | 0,80 | 12750 |
| 78 | 1600001 | 1,17 | 23000 | 192 | 3748376 | 0,49 | 4750 |
| 79 | 1633626 | 0,73 | 7250 | 193 | 3766751 | 0,38 | 1500 |
| 80 | 1654626 | 0,74 | 8750 | 194 | 3773376 | 0,85 | 750 |
| 81 | 1668501 | 0,79 | 5000 | 195 | 3779001 | 0,99 | 7500 |
| 82 | 1678126 | 0,61 | 5750 | 196 | 3802376 | 0,49 | 3750 |
| 83 | 1692251 | 0,40 | 1000 | 197 | 3829501 | 0,91 | 8500 |
| 84 | 1698876 | 0,94 | 7750 | 198 | 3867126 | 0,97 | 6250 |
| 85 | 1733751 | 1,03 | 4000 | 199 | 3879126 | 0,58 | 6250 |
| 86 | 1741876 | 0,52 | 5250 | 200 | 3890751 | 0,53 | 6000 |
| 87 | 1759751 | 0,40 | 2500 | 201 | 3897876 | 0,53 | 1250 |
| 88 | 1787126 | 0,61 | 4750 | 202 | 3908876 | 0,93 | 9750 |
| 89 | 1792376 | 0,64 | 4750 | 203 | 3916876 | 0,40 | 750 |

| | | | | | | | |
|---|---|---|---|---|---|---|---|
| 90 | 1805876 | 0,90 | 7750 | 204 | 3933376 | 0,34 | 2250 |
| 91 | 1842501 | 0,97 | 6000 | 205 | 3947876 | 0,40 | 2750 |
| 92 | 1869876 | 0,23 | 1250 | 206 | 3960501 | 0,83 | 16500 |
| 93 | 1887626 | 0,65 | 6750 | 207 | 3998876 | 0,99 | 6250 |
| 94 | 1933251 | 1,16 | 4500 | 208 | 4026376 | 1,10 | 6750 |
| 95 | 1946126 | 0,63 | 5750 | 209 | 4044001 | 1,06 | 5500 |
| 96 | 1976126 | 0,86 | 5250 | 210 | 4062501 | 0,68 | 9000 |
| 97 | 1987626 | 0,43 | 3250 | 211 | 4072501 | 0,79 | 6500 |
| 98 | 1991251 | 0,40 | 1000 | 212 | 4110751 | 0,87 | 9000 |
| 99 | 2011501 | 0,58 | 8000 | 213 | 4132751 | 0,95 | 8000 |
| 100 | 2019876 | 0,49 | 2250 | 214 | 4168501 | 0,73 | 2000 |
| 101 | 2035376 | 0,50 | 3250 | 215 | 4177001 | 0,61 | 10500 |
| 102 | 2041001 | 0,60 | 1000 | 216 | 4191626 | 0,56 | 6250 |
| 103 | 2078501 | 1,13 | 71500 | 217 | 4219251 | 0,70 | 6500 |
| 104 | 2127001 | 0,86 | 13500 | 218 | 4234626 | 0,43 | 6250 |
| 105 | 2141126 | 0,53 | 2250 | 219 | 4257001 | 0,87 | 8000 |
| 106 | 2149626 | 1,00 | 3750 | 220 | 4279501 | 0,97 | 8500 |
| 107 | 2174251 | 0,87 | 6500 | 221 | 4300126 | 0,40 | 4250 |
| 108 | 2188626 | 0,42 | 9250 | 222 | 4312001 | 0,85 | 5000 |
| 109 | 2210126 | 0,61 | 5250 | 223 | 4334376 | 1,01 | 10250 |
| 110 | 2222876 | 0,51 | 5750 | 224 | 4388251 | 0,57 | 10500 |
| 111 | 2250626 | 0,90 | 13250 | 225 | 4416126 | 0,80 | 15750 |
| 112 | 2268751 | 0,63 | 4000 | 226 | 4427751 | 0,39 | 2000 |
| 113 | 2292876 | 0,64 | 7750 | 227 | 4435376 | 0,63 | 10750 |
| 114 | 2303751 | 0,80 | 13000 | 228 | 4455251 | 0,81 | 25000 |

**Figure S2**

Maximal distance analysis between neighboring cohesin peaks. 10,000 random cohesin patterns, distributed among the convergent sites along fission yeast chromosome 2, were created using a bootstrapping approach. The number and widths of the distributed cohesin peaks were those from the observed binding pattern, as assigned in Figure S1. The maximal distance between neighboring cohesin peaks in each of the random distributions was determined and is shown in the histogram below. The red line indicates the maximal distance between two neighboring cohesin-bound convergent sites in the actual observed cohesin pattern.

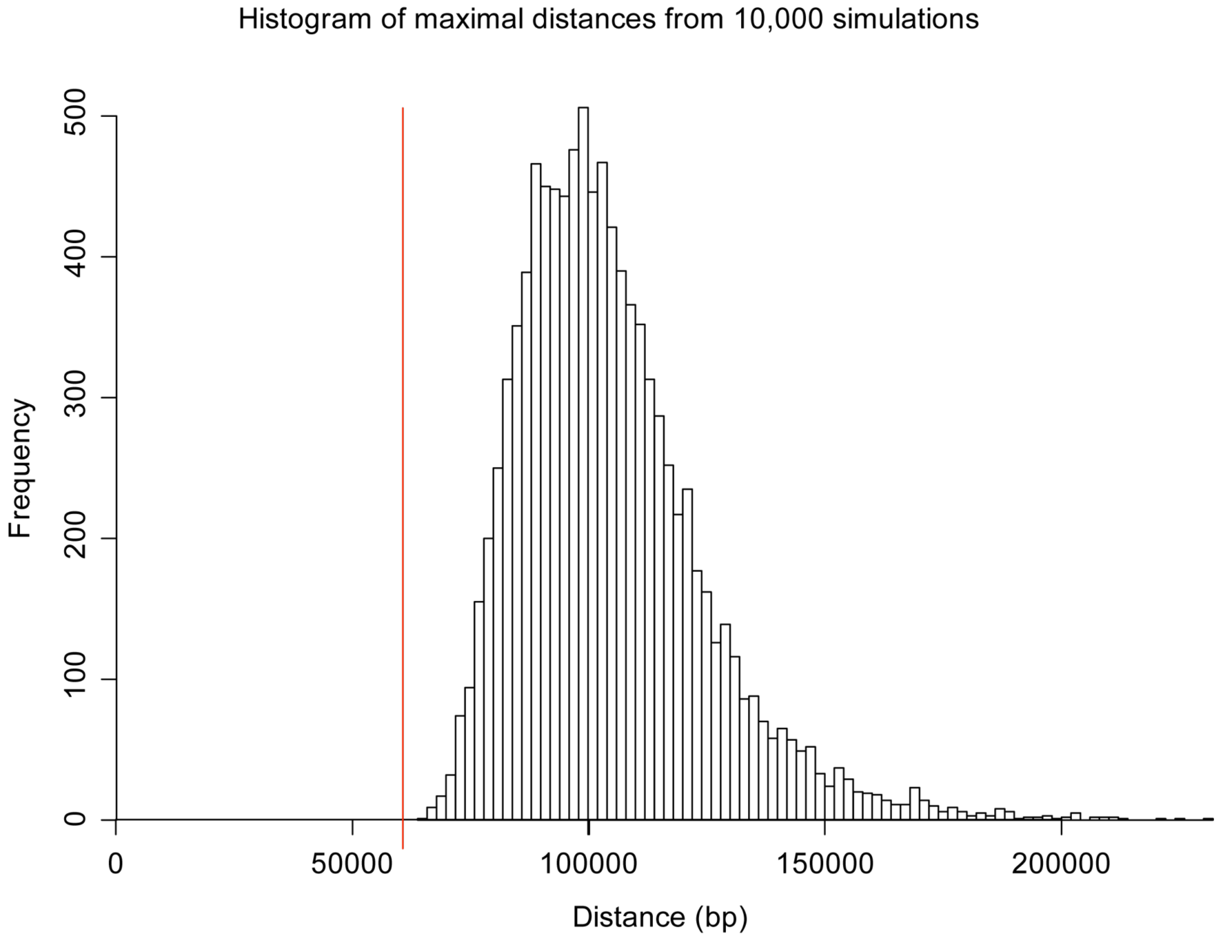

**Figure S3**

Higher expression levels of convergent gene pairs flanking cohesin binding sites. The distribution of relative mRNA levels from genes flanking cohesin-bound and cohesin-free convergent sites along chromosome 2 is shown. Boxes indicate boundaries of the 25th to the 75th percentile. The median is shown as a bold black line, whiskers extend to 1.5 times the interquartile range, outliers are marked as circles. A Wilcoxon signed ranks test suggests that genes flanking cohesin-bound convergent sites are more strongly expressed than genes flanking cohesin-free convergent sites (p=0.011).

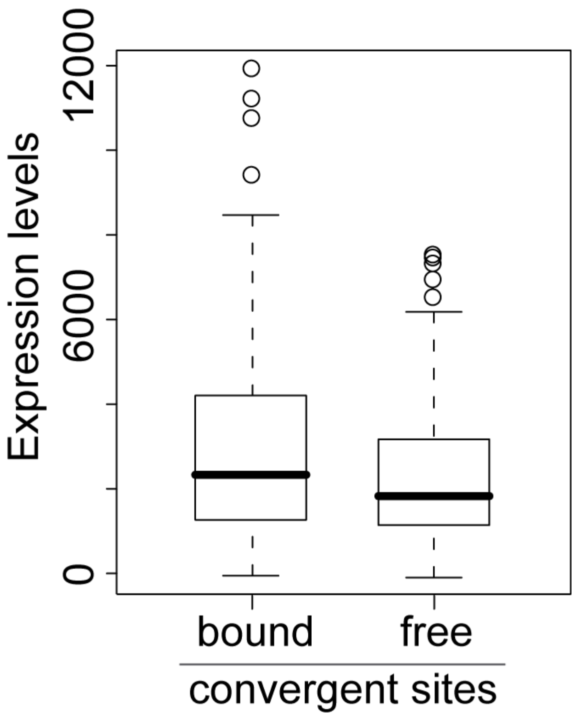

**Figure S4**

The cohesin loader Mis4/Ssl3 overlaps with tRNA and ribosomal protein genes. (**a**) Binding profile of Mis4 and Ssl3 along fission yeast chromosome 2. Chromatin immunoprecipitation was performed against epitope-tagged Mis4-$Pk_9$ and Ssl3-$Pk_9$ from exponentially proliferating cells. As a control, cells without epitope-tagged protein were grown under identical conditions and processed in parallel for chromatin immunoprecipitation with an $\alpha$-Pk antibody. The map shows an overlay of Mis4 (green), Ssl3 (blue) and the untagged control (purple), as described in Figure S1. Peaks were assigned as detailed in Figure S1, but the threshold for local maxima was a signal intensity of 0.5. The untagged control sample yielded trace amounts of immunoprecipitated DNA, which after amplification led to several strong peaks often in intergenic low complexity regions. Several of these peaks overlapped with Mis4/Ssl3 peaks, which were excluded from the analysis. Peaks in low complexity regions were not observed in chromatin immunoprecipitates of cohesin subunits (compare Figure S1). (**b**) Table of the 72 specific Mis4 peaks assigned as described in (a) along chromosome 2. The position, average height and width of each of the peaks is indicated. The actual number of Mis4 bindings sites might be higher, as some peaks in low complexity regions that have been removed from the analysis may be significant, while others may have remained below recognition by our peak picking parameters. (**c**) The distances of the 72 Mis4 binding sites to the nearest tRNA gene (blue) is shown. 16 lie within 5 kb. The distances of the remaining 56 peaks to the nearest ribosomal protein gene (red) are also shown, of which 18 lie again within 5 kb.

(a)

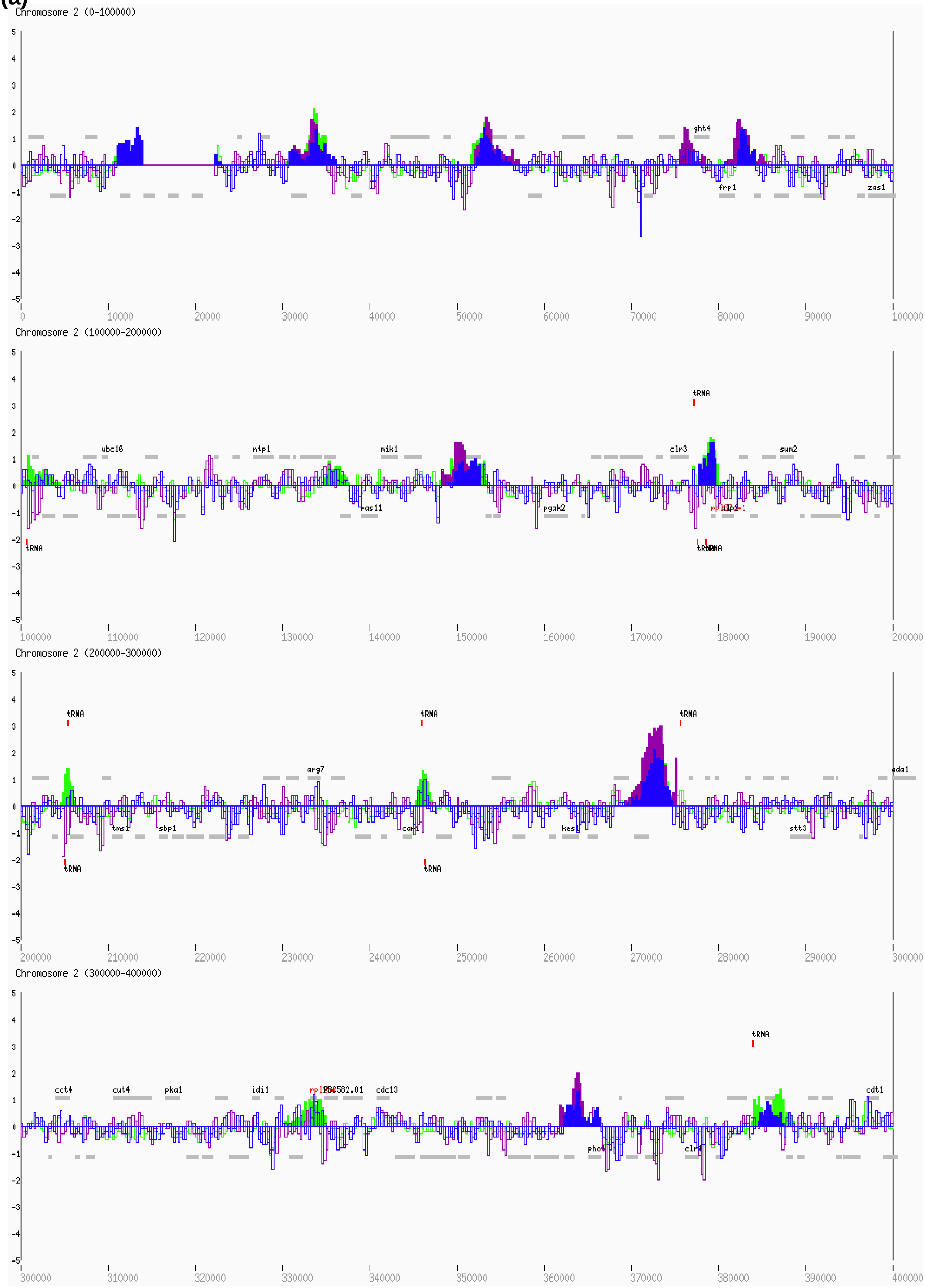
Chromosome 2 (0-100000)
ght4
frp1
zas1
Chromosome 2 (100000-200000)
ubc16
ntp1
mik1
clr3
sum2
ras11
pgak2
tRNA
Chromosome 2 (200000-300000)
tRNA
arg7
tms1
sbp1
cam1
kes1
stt3
ada1
Chromosome 2 (300000-400000)
cct4
cut4
pka1
idi1
cdc13
pho4
cdt1

Chromosome 2 (400000-500000)

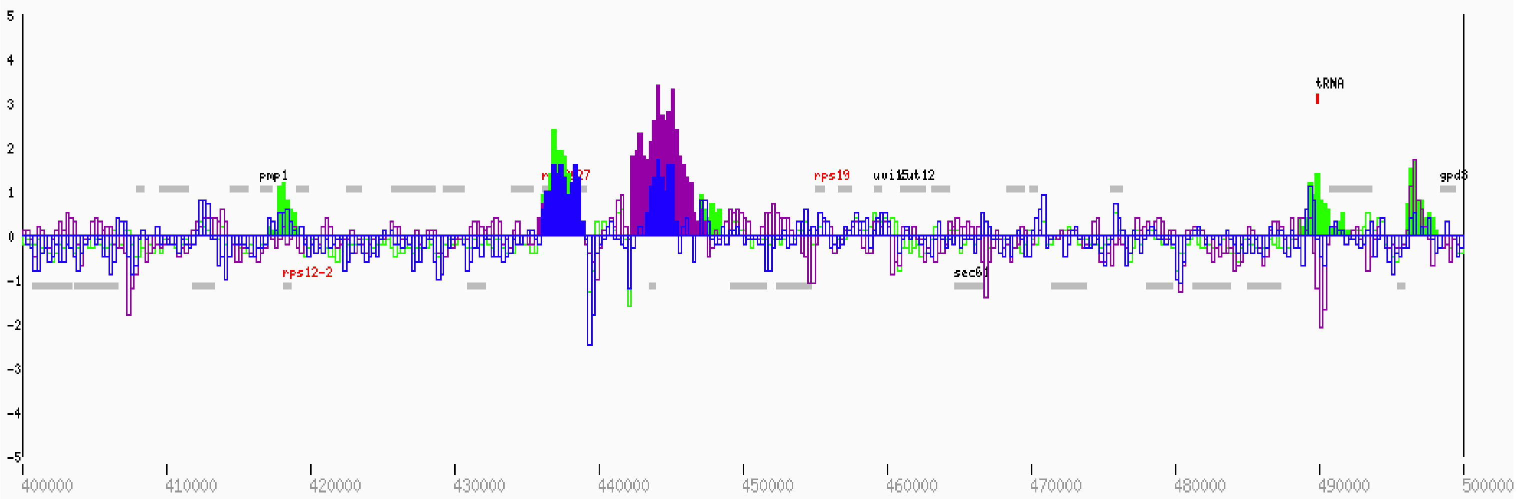


Chromosome 2 (500000-600000)

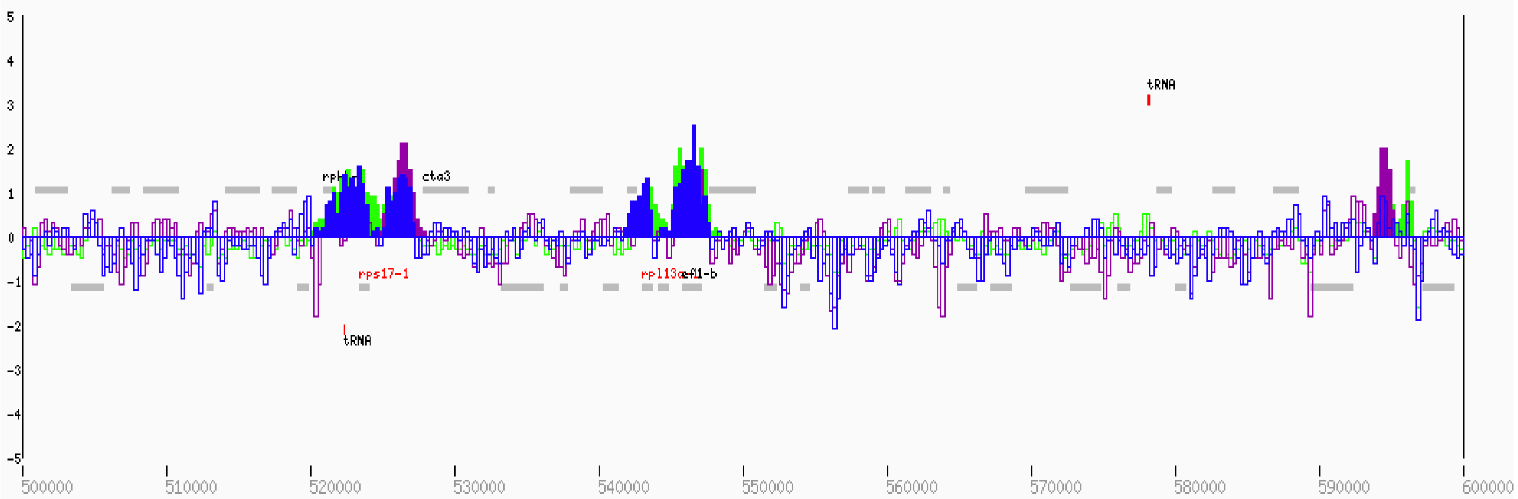


Chromosome 2 (600000-700000)

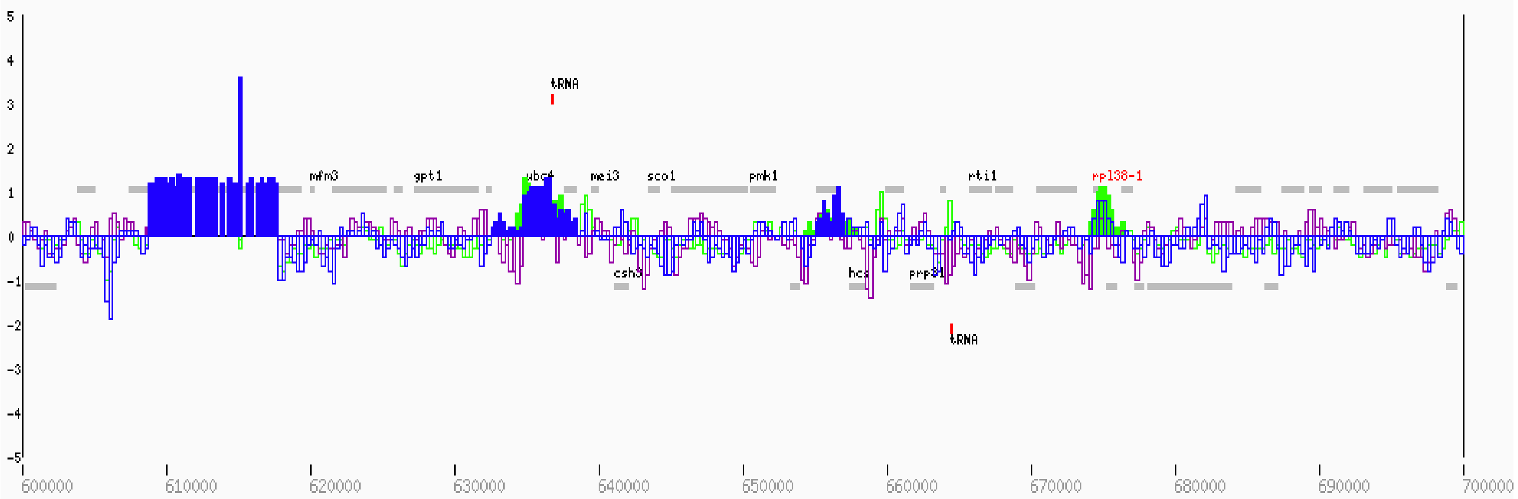


Chromosome 2 (700000-800000)

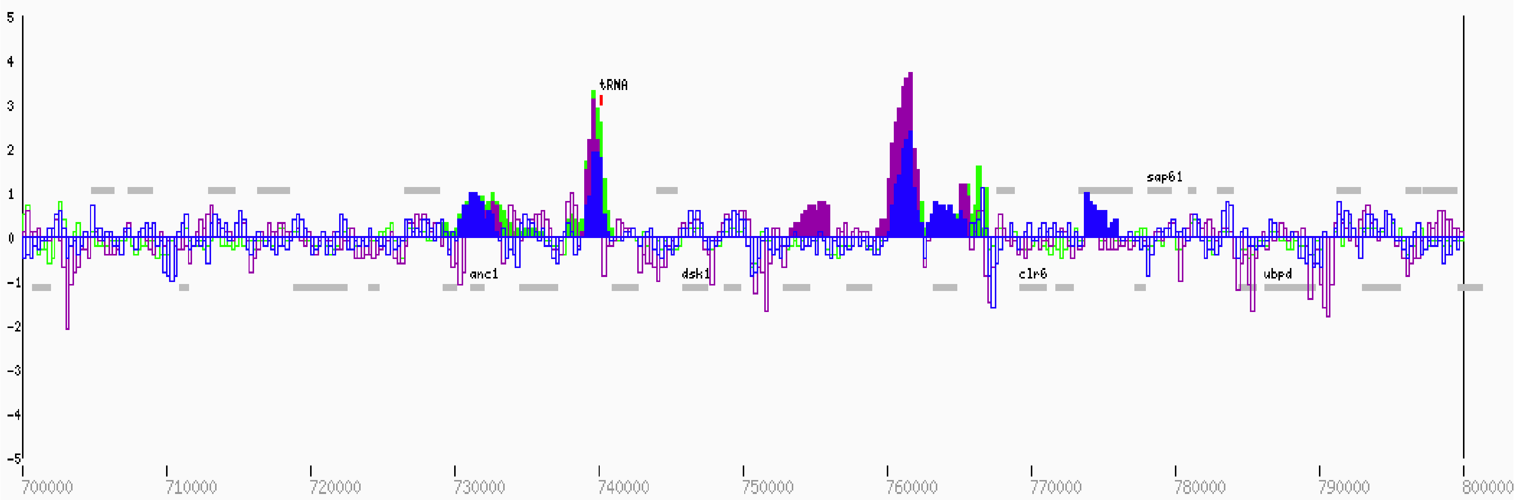

Chromosome 2 (800000-900000)

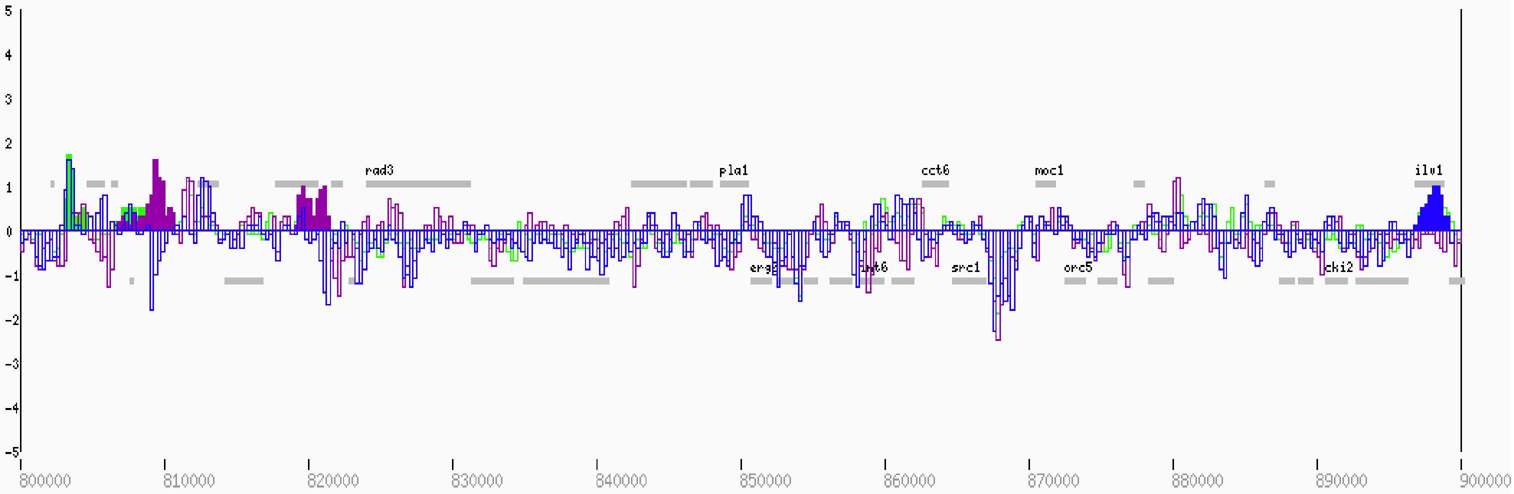


Chromosome 2 (900000-1000000)

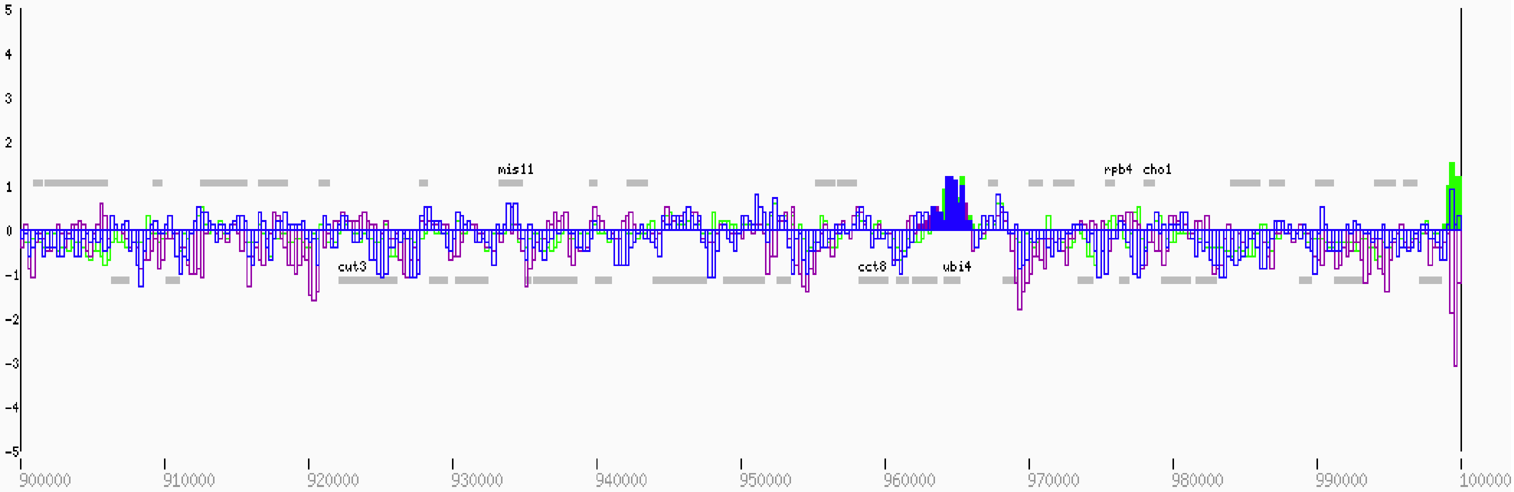


Chromosome 2 (1000000-1100000)

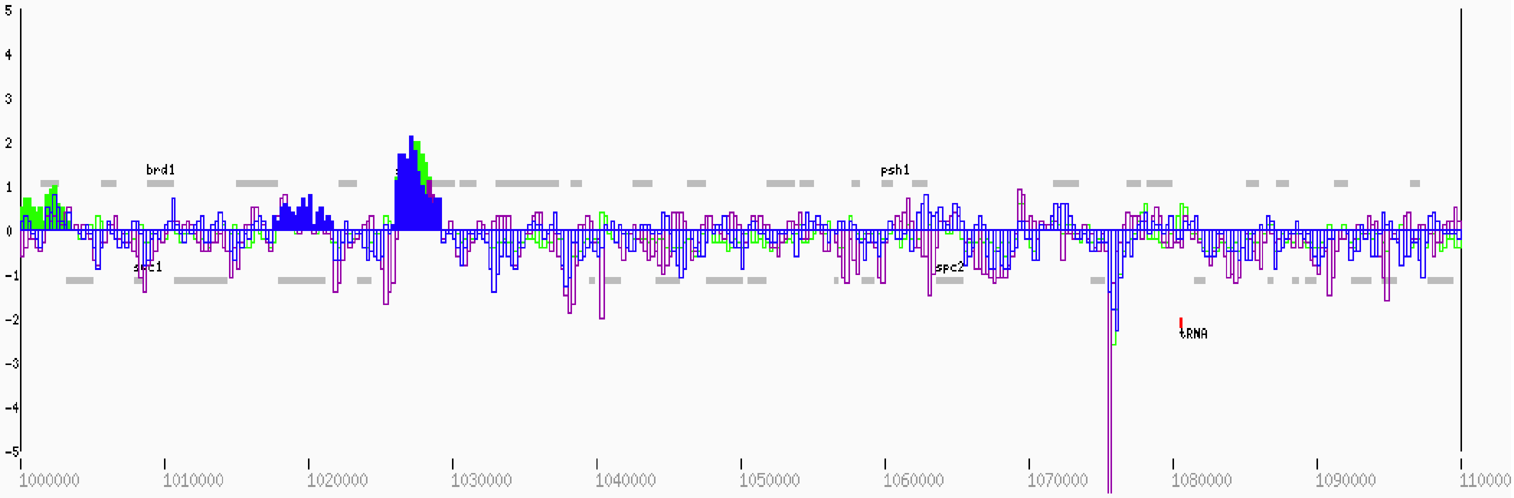


Chromosome 2 (1100000-1200000)

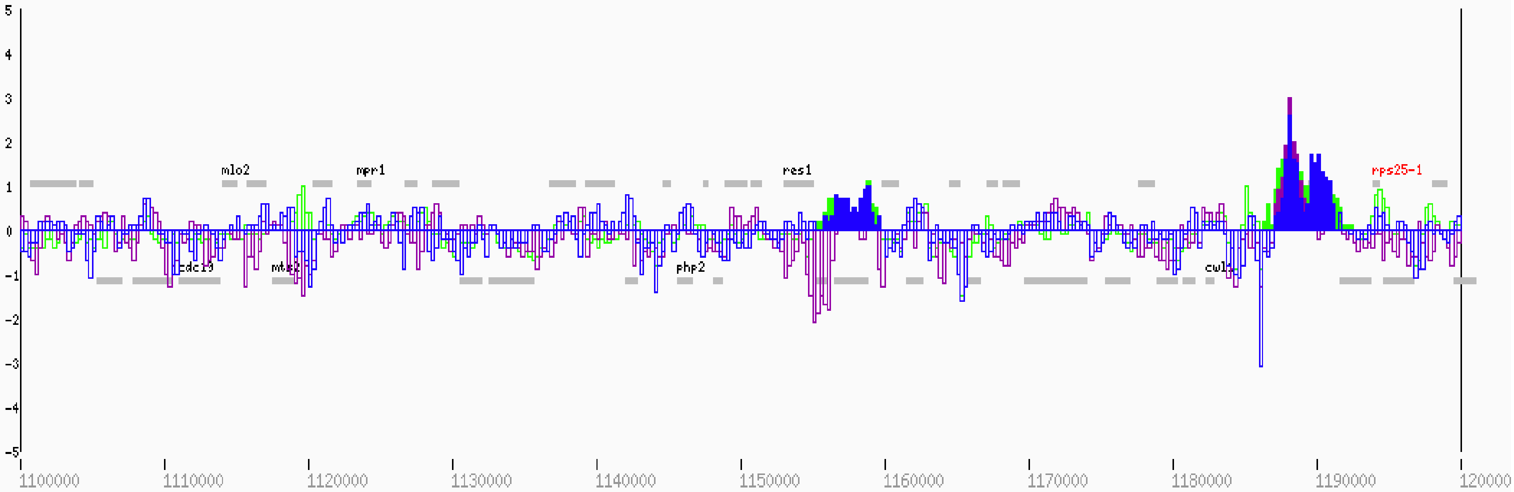

Chromosome 2 (1200000-1300000)

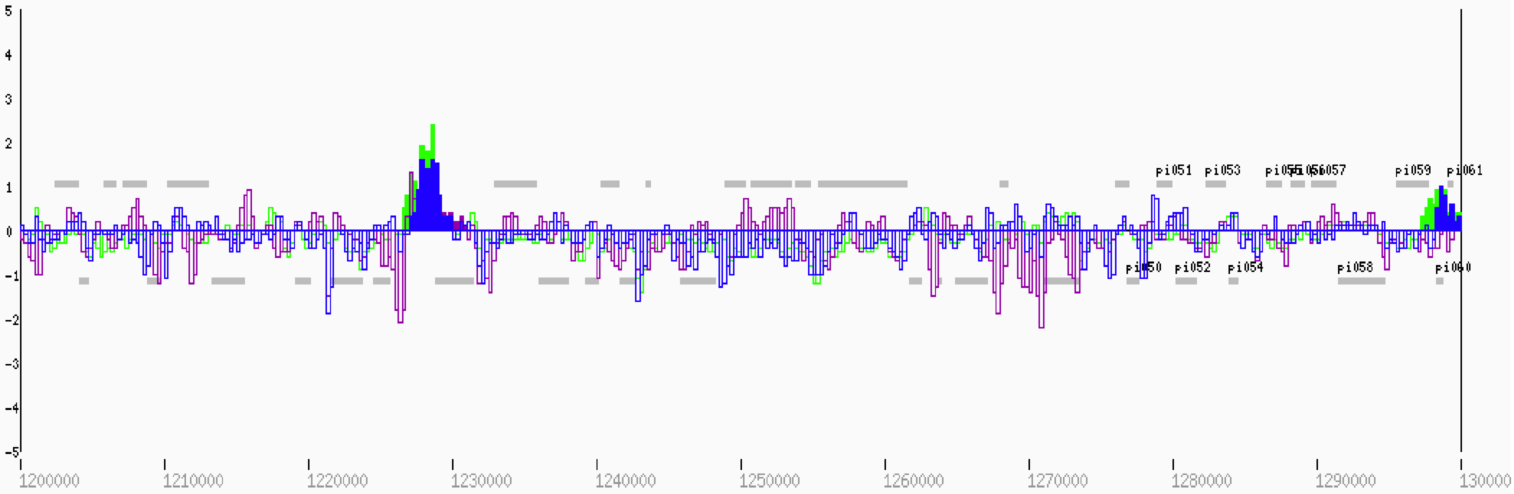


Chromosome 2 (1300000-1400000)

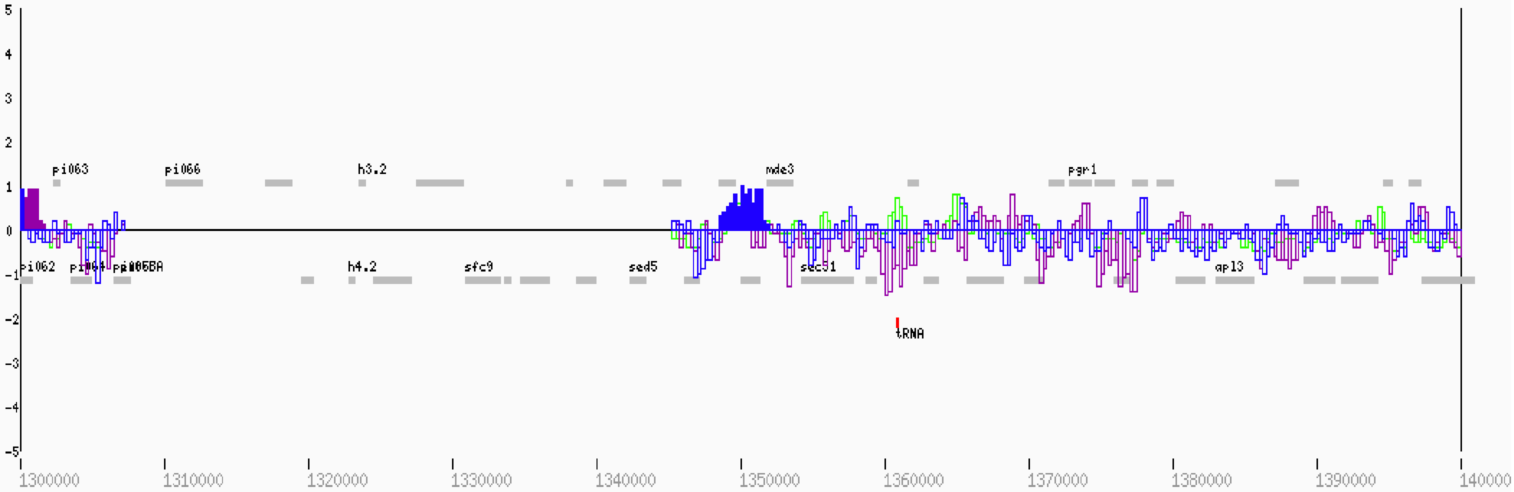


Chromosome 2 (1400000-1500000)

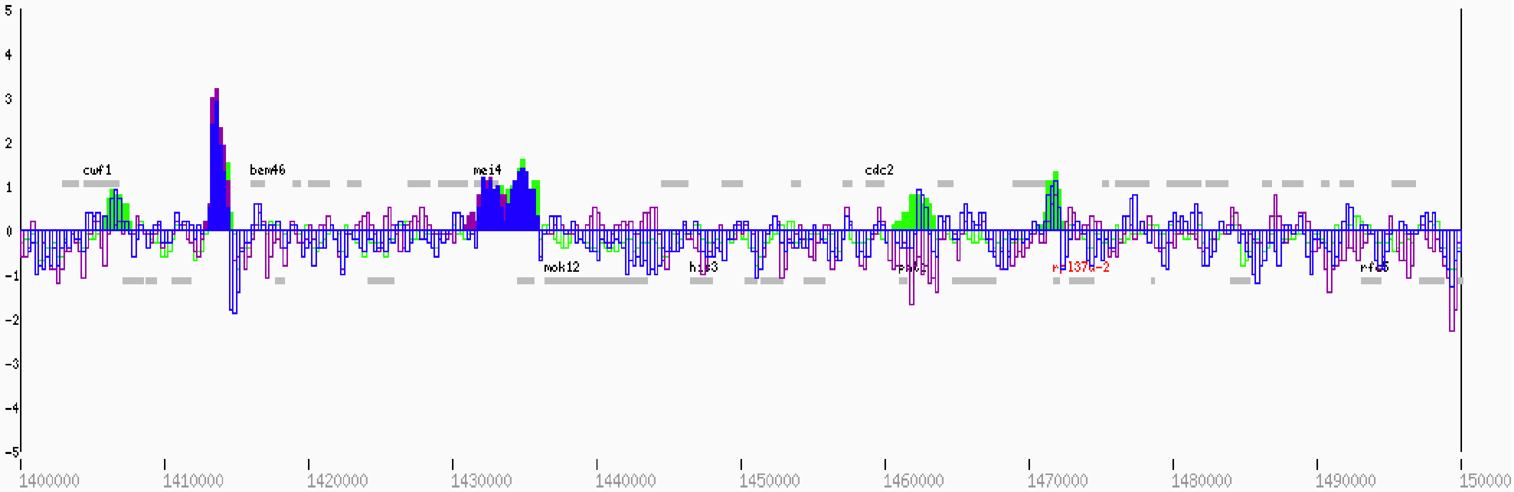


Chromosome 2 (1500000-1600000)

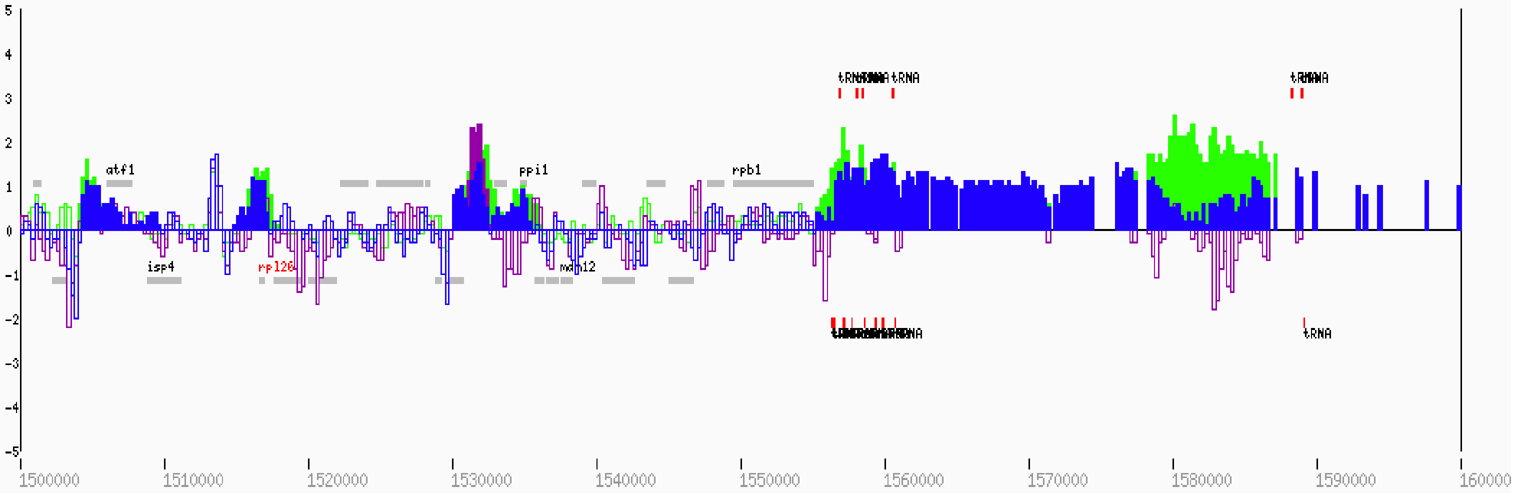

Chromosome 2 (1600000-1700000)

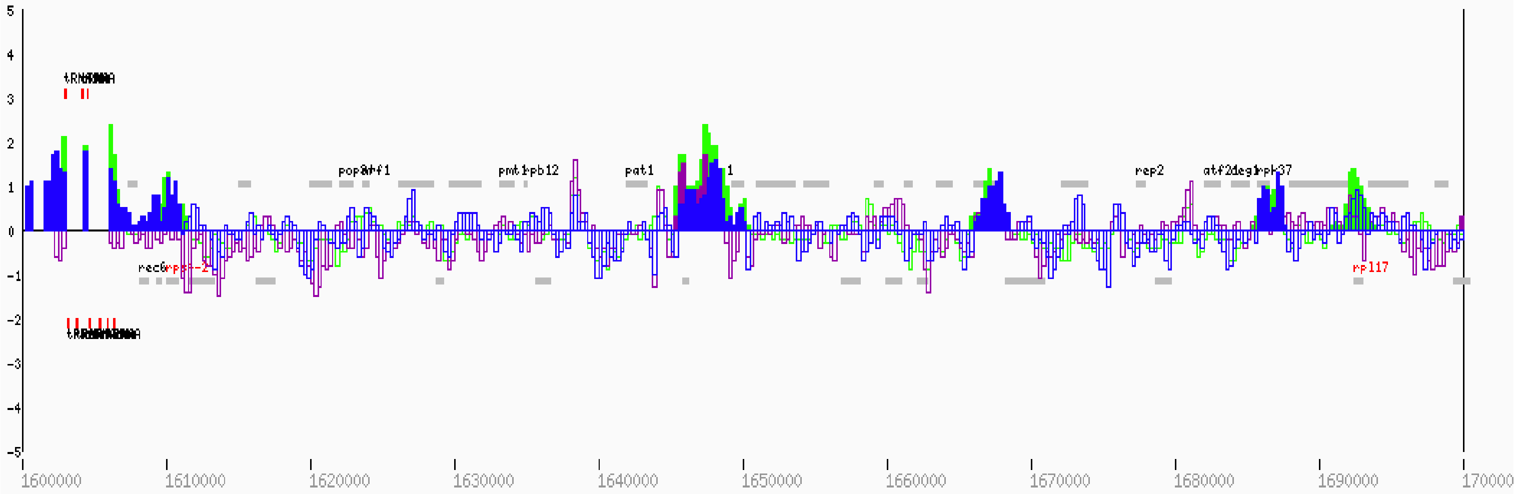


Chromosome 2 (1700000-1800000)

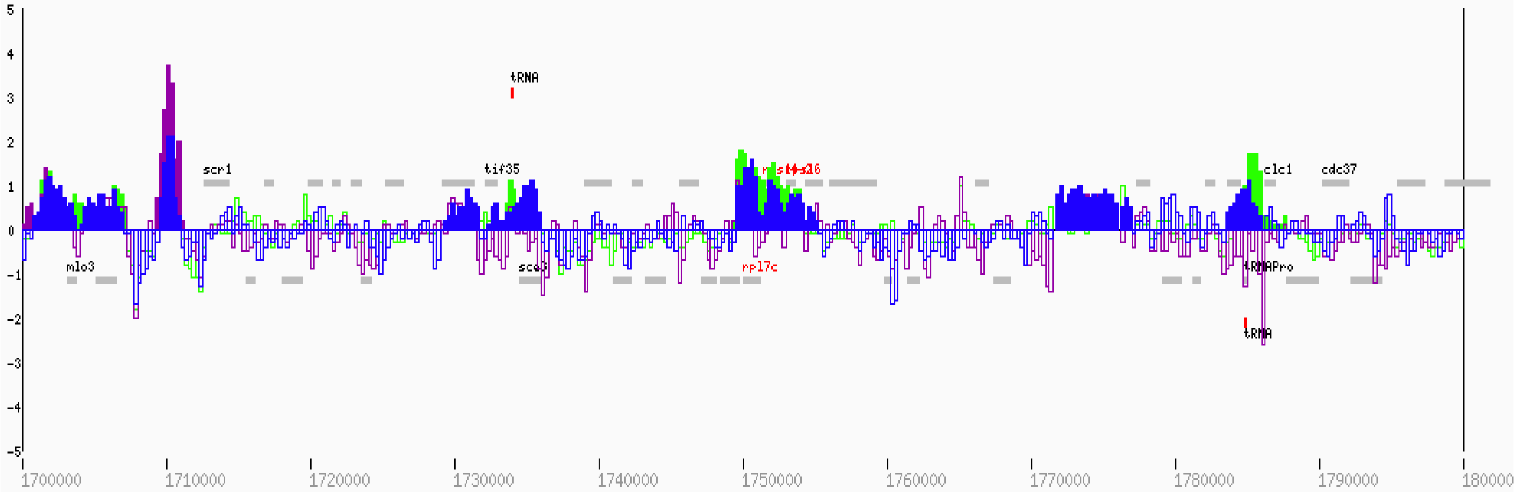


Chromosome 2 (1800000-1900000)

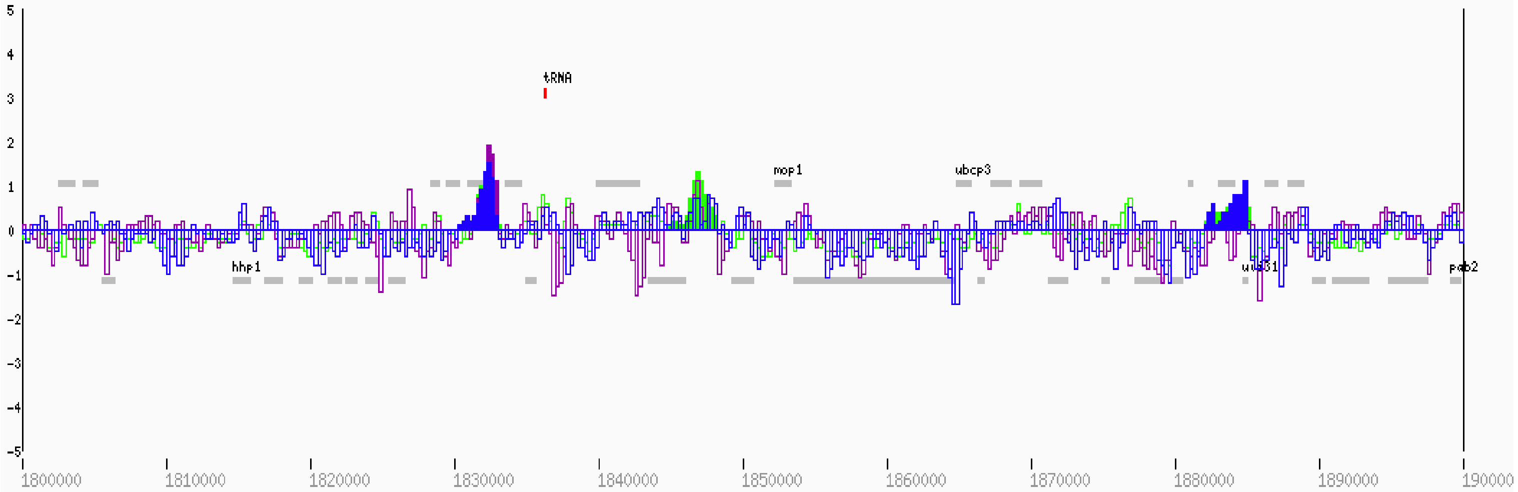


Chromosome 2 (1900000-2000000)

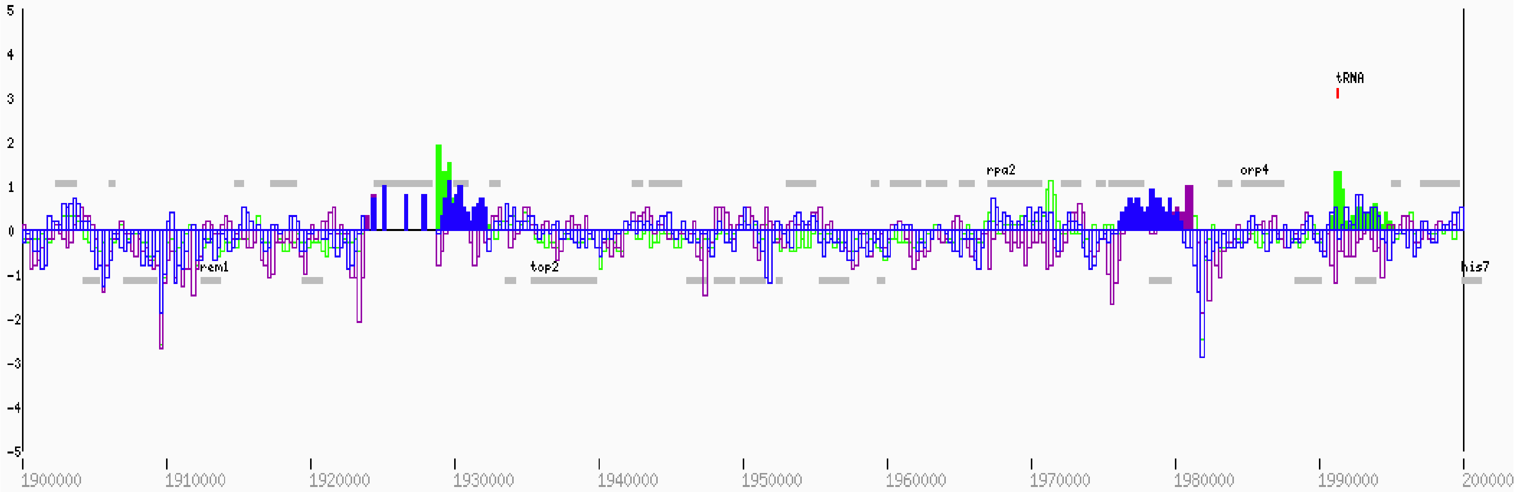

Chromosome 2 (2000000-2100000)

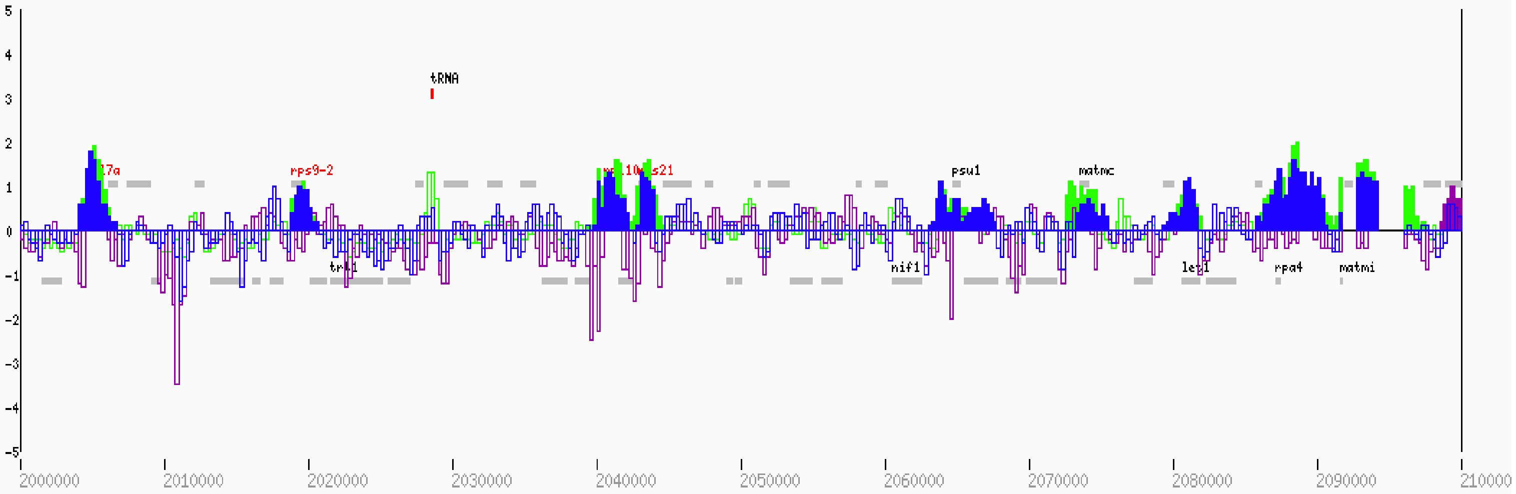


Chromosome 2 (2100000-2200000)

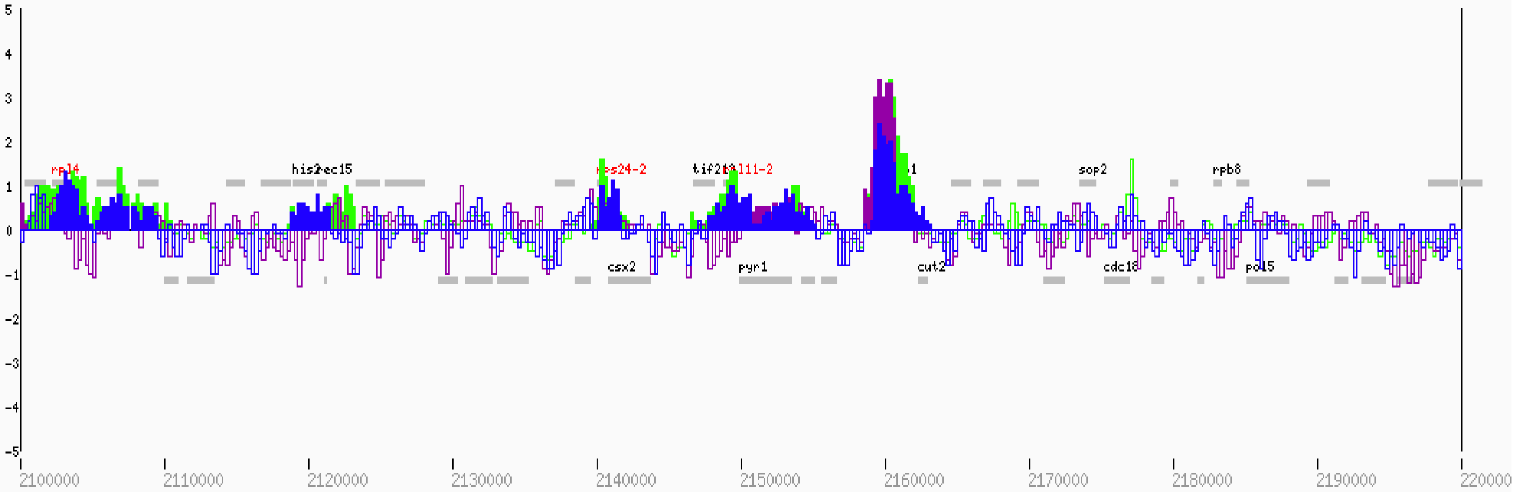


Chromosome 2 (2200000-2300000)

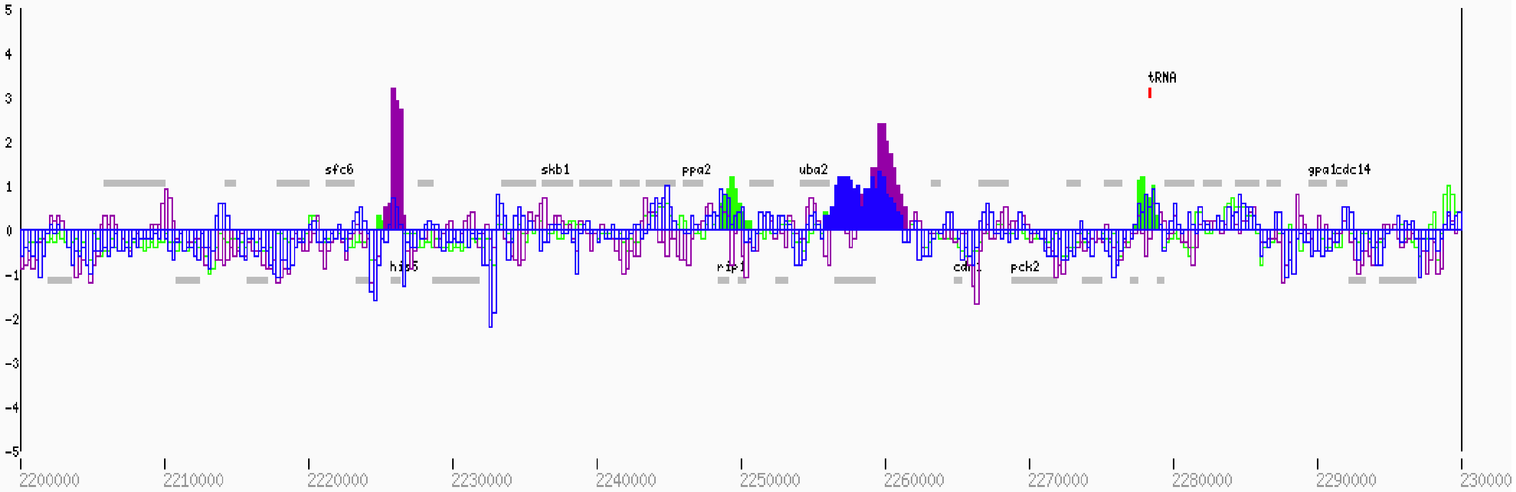


Chromosome 2 (2300000-2400000)

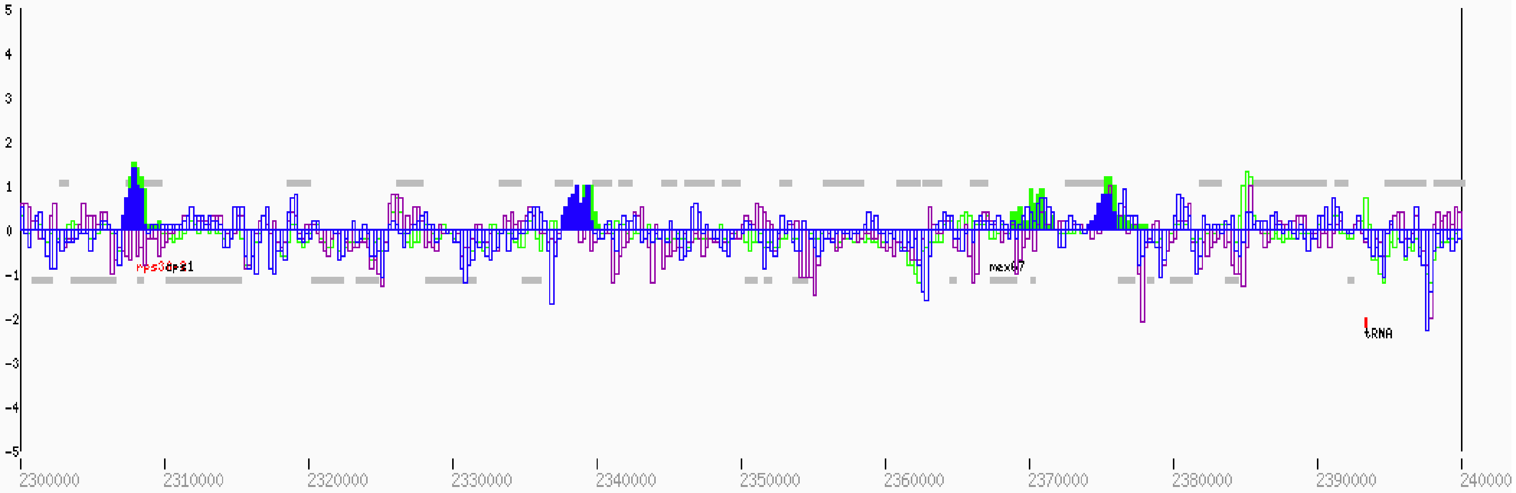

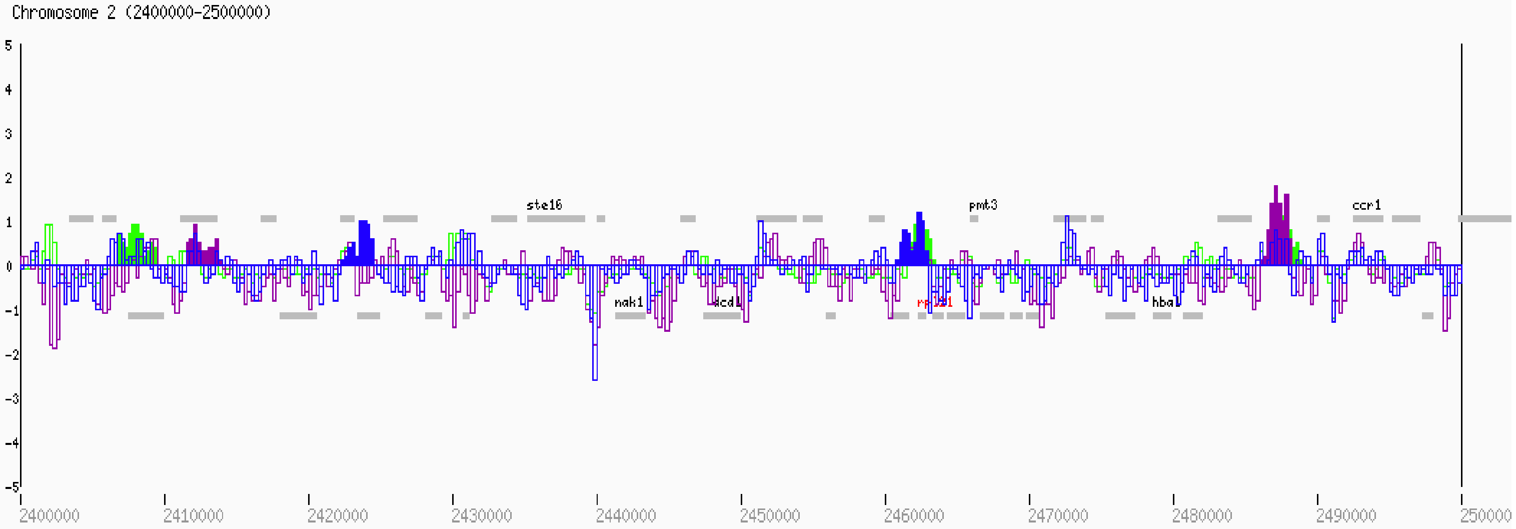
Chromosome 2 (2400000-2500000)
ste16
pmt3
ccr1
mak1
hba1
2400000
2410000
2420000
2430000
2440000
2450000
2460000
2470000
2480000
2490000
2500000

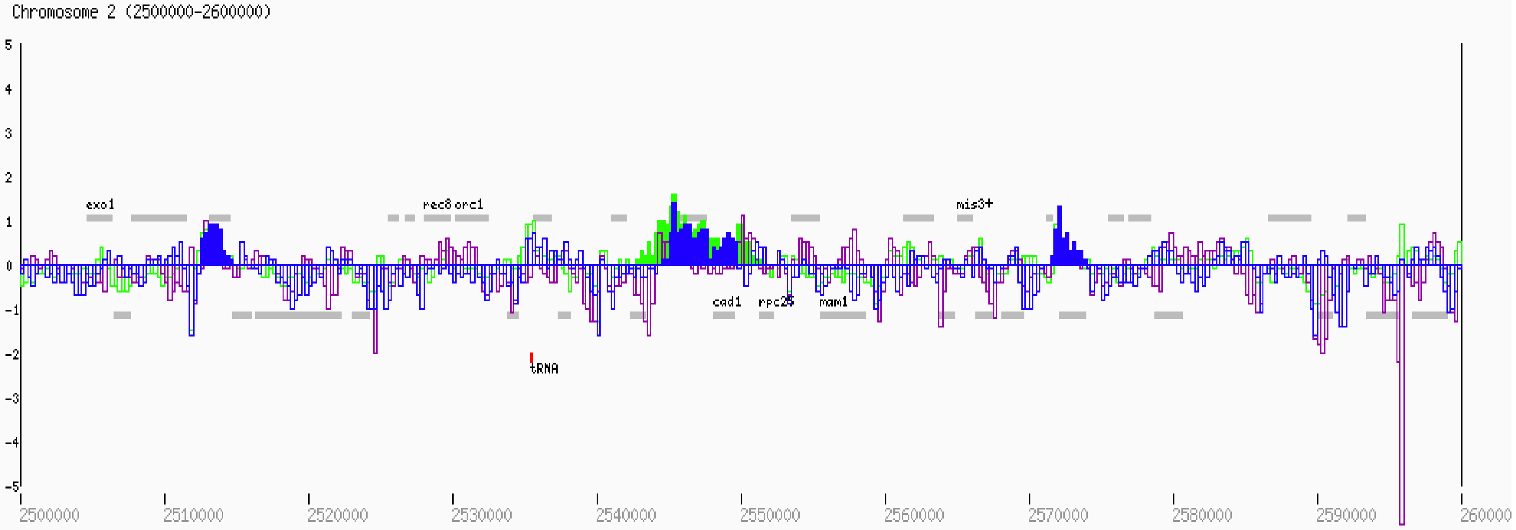
Chromosome 2 (2500000-2600000)
exo1
rec8
orc1
mis3+
cad1
rpc25
mam1
tRNA
2500000
2510000
2520000
2530000
2540000
2550000
2560000
2570000
2580000
2590000
2600000

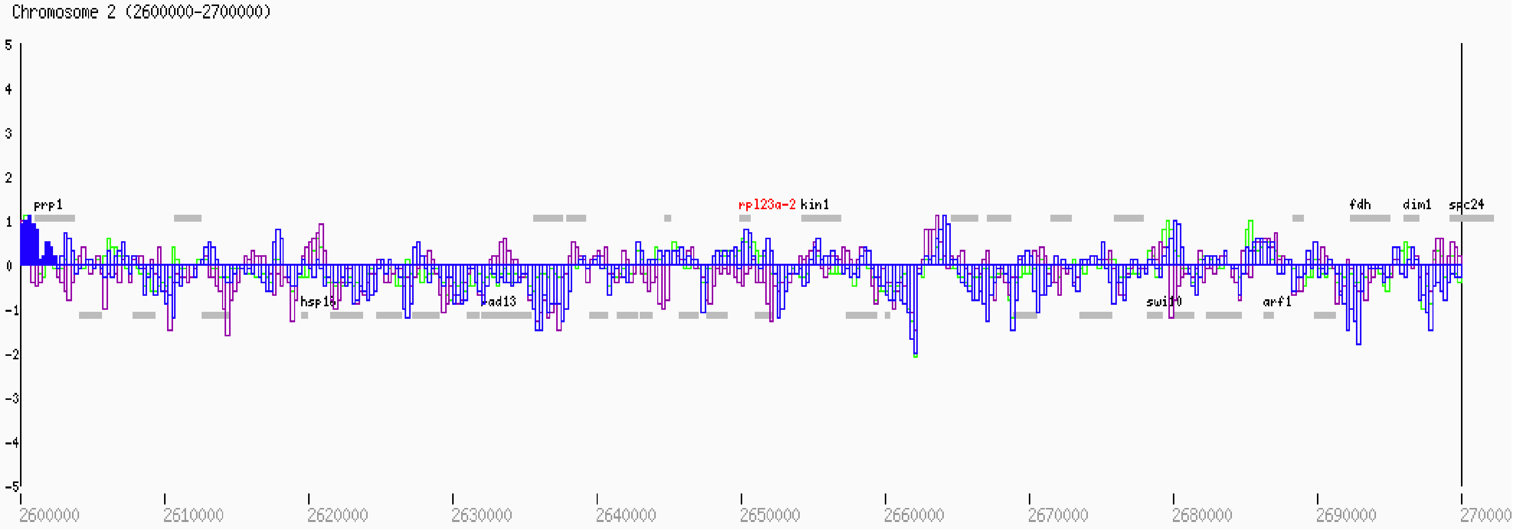
Chromosome 2 (2600000-2700000)
prp1
rpl23a-2
kin1
fdh
dim1
spc24
hsp16
rad13
arf1
2600000
2610000
2620000
2630000
2640000
2650000
2660000
2670000
2680000
2690000
2700000

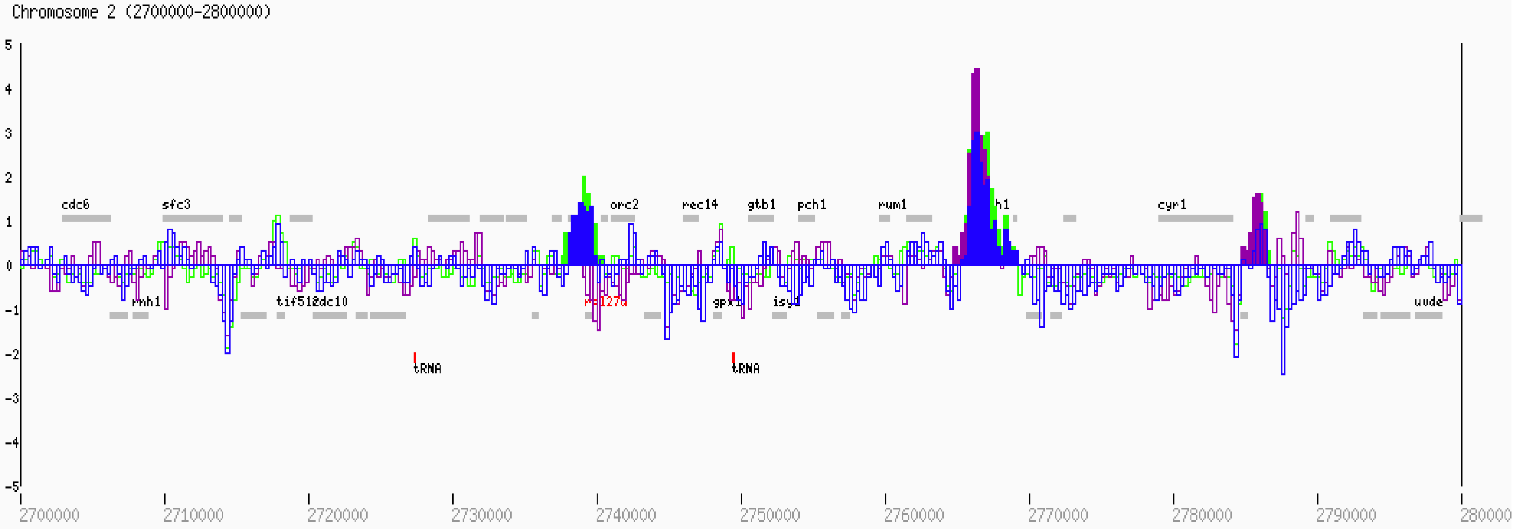
Chromosome 2 (2700000-2800000)
cdc6
sfc3
orc2
rec14
gtb1
pch1
rum1
cyr1
rnh1
tif512
cdc10
gpx1
uvde
tRNA
tRNA
2700000
2710000
2720000
2730000
2740000
2750000
2760000
2770000
2780000
2790000
2800000

Chromosome 2 (2800000-2900000)

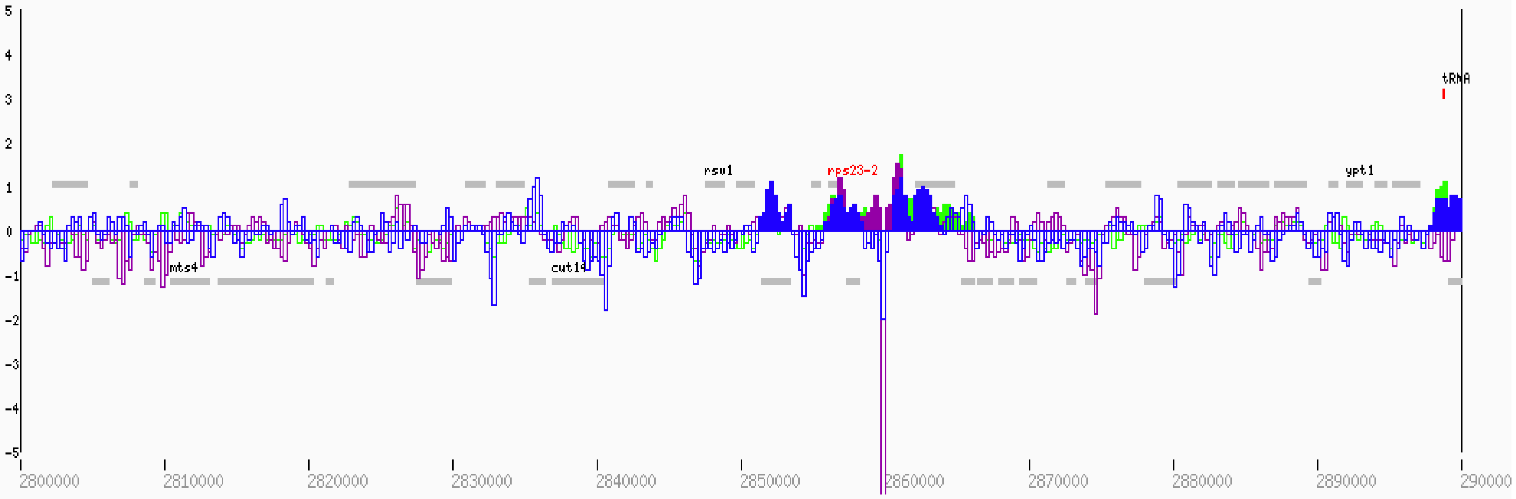


Chromosome 2 (2900000-3000000)

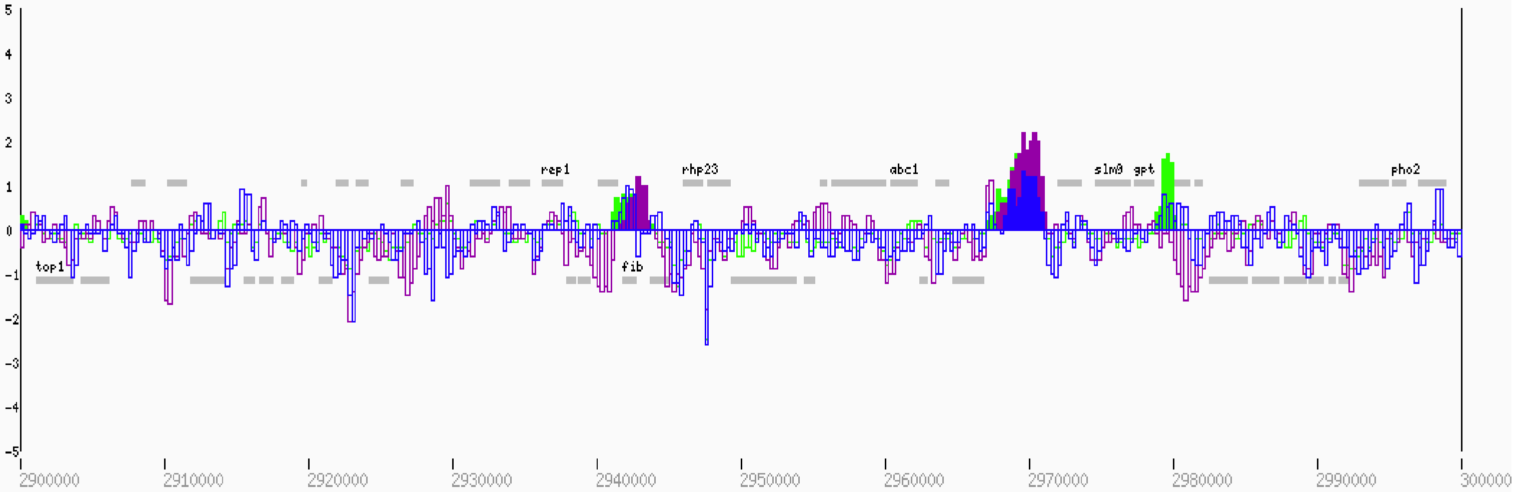


Chromosome 2 (3000000-3100000)

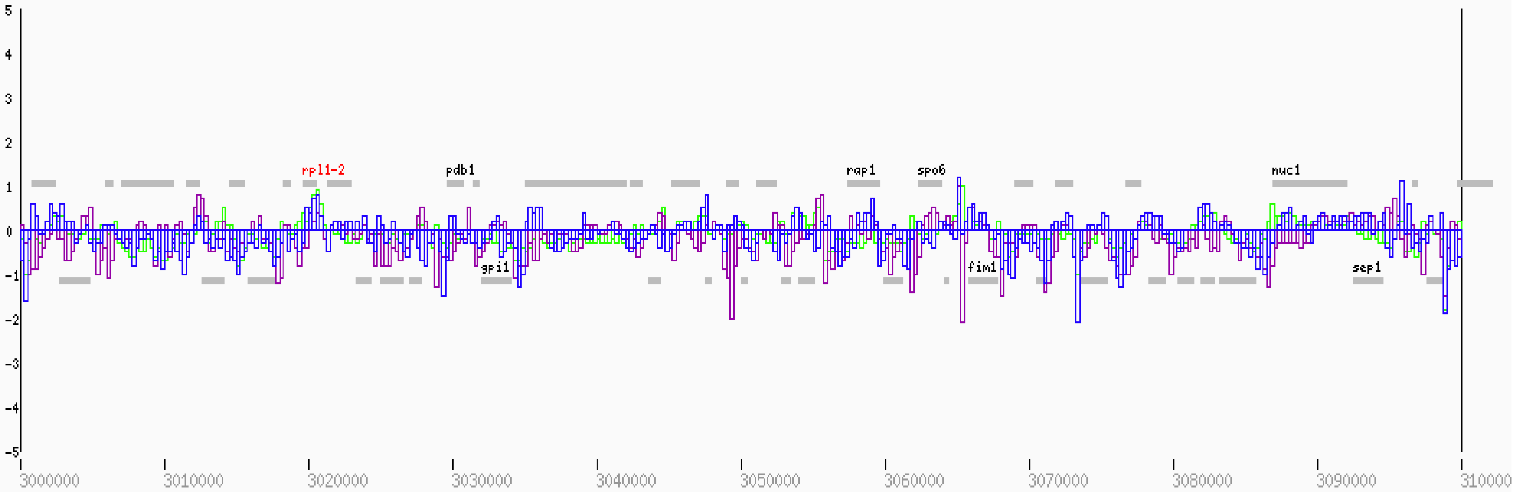


Chromosome 2 (3100000-3200000)

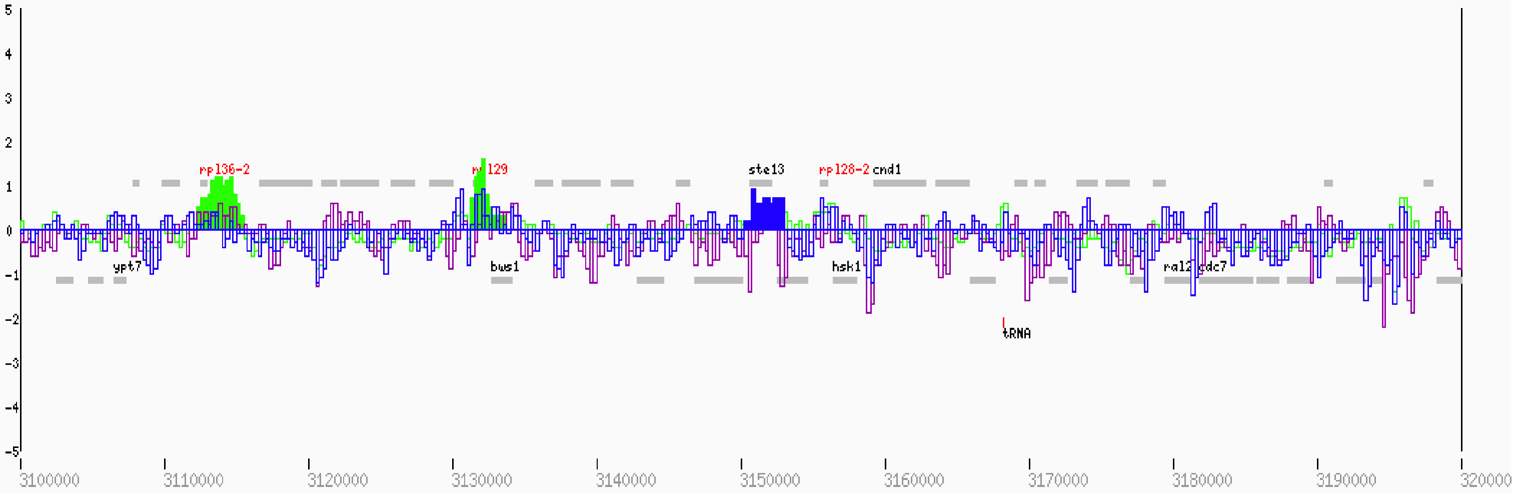

Chromosome 2 (3200000-3300000)

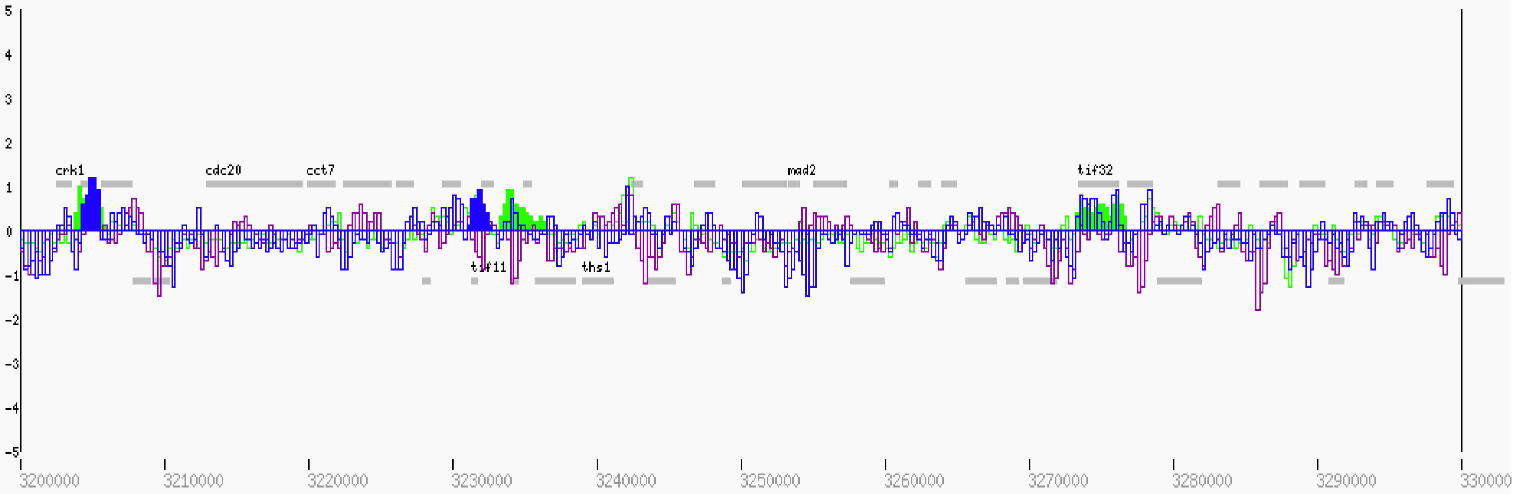


Chromosome 2 (3300000-3400000)

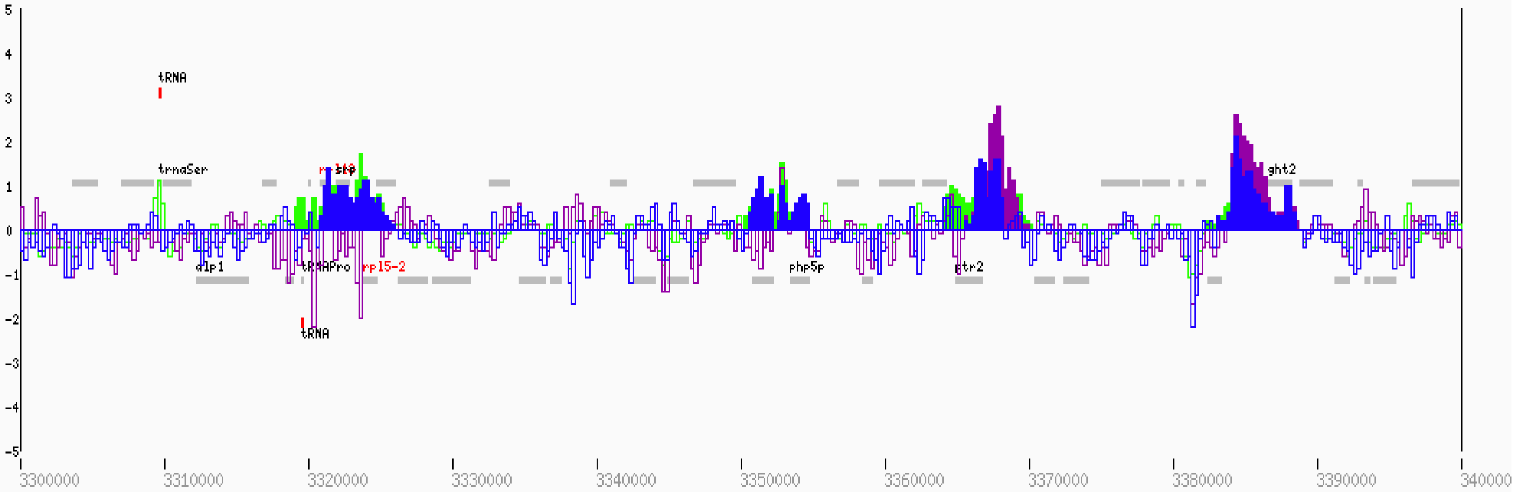


Chromosome 2 (3400000-3500000)

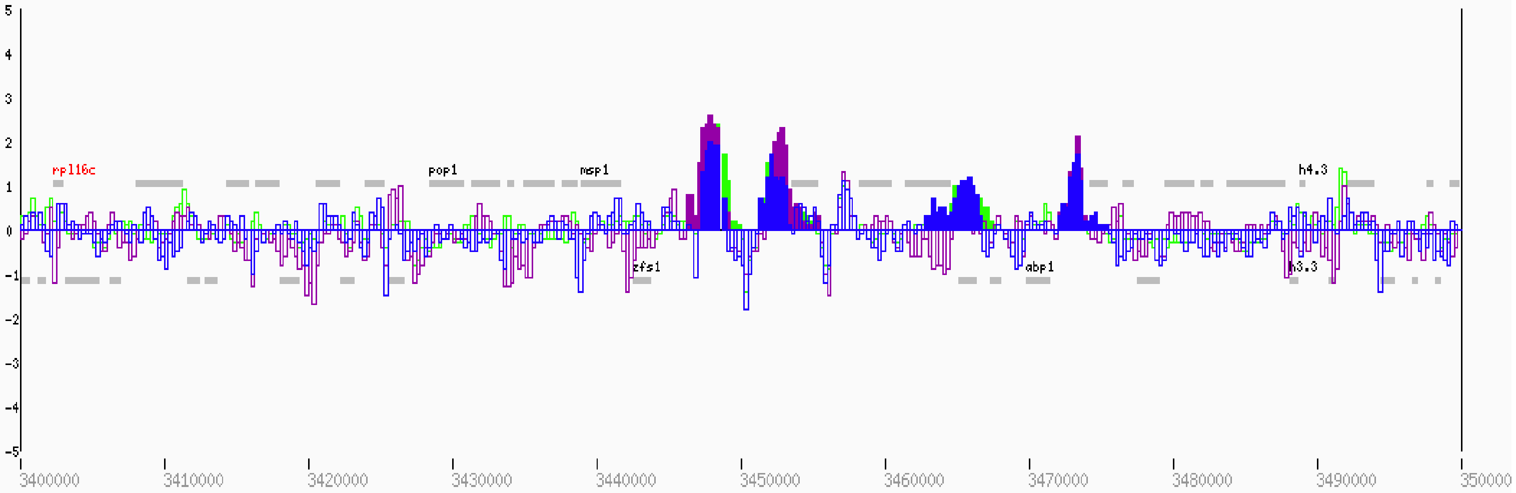


Chromosome 2 (3500000-3600000)

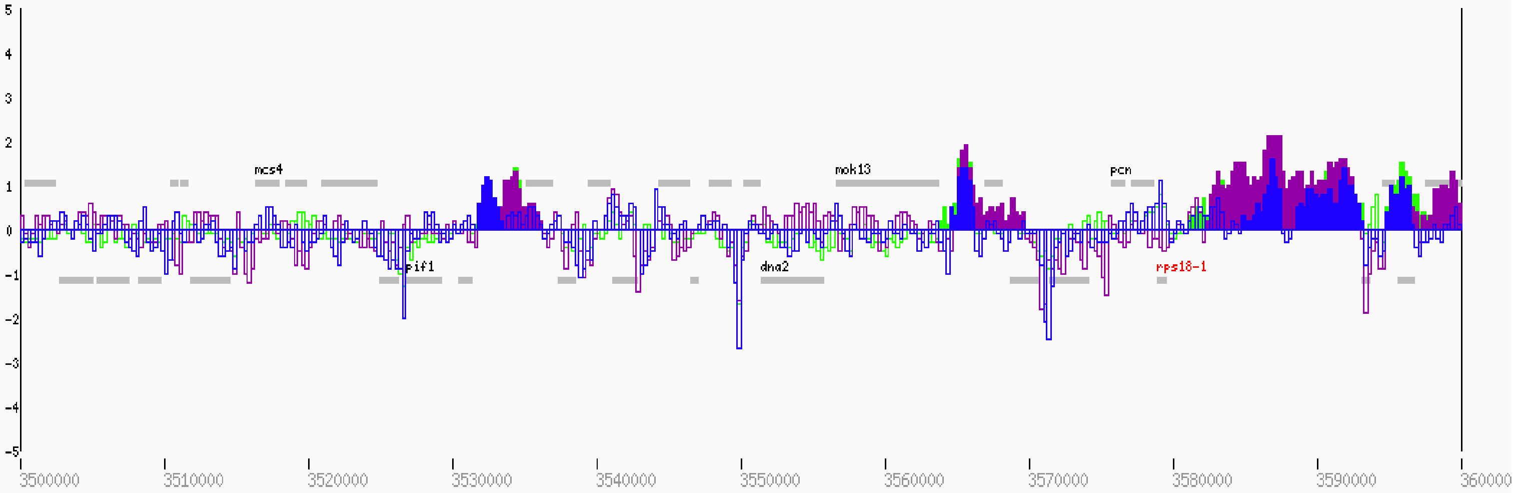

Chromosome 2 (3600000-3700000)

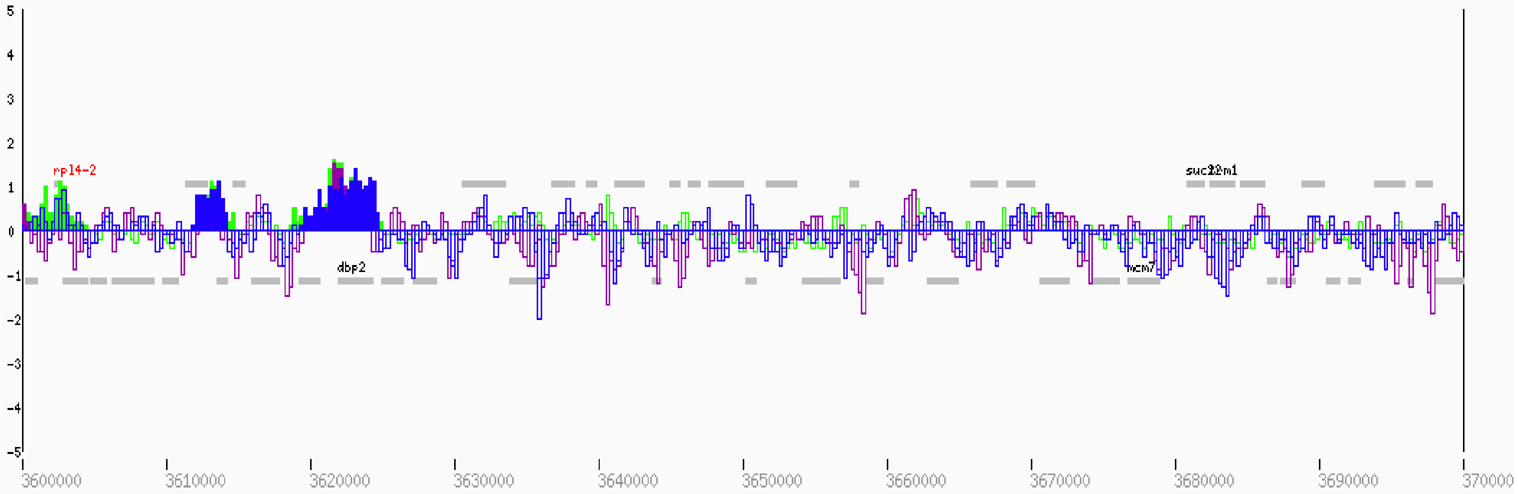


Chromosome 2 (3700000-3800000)

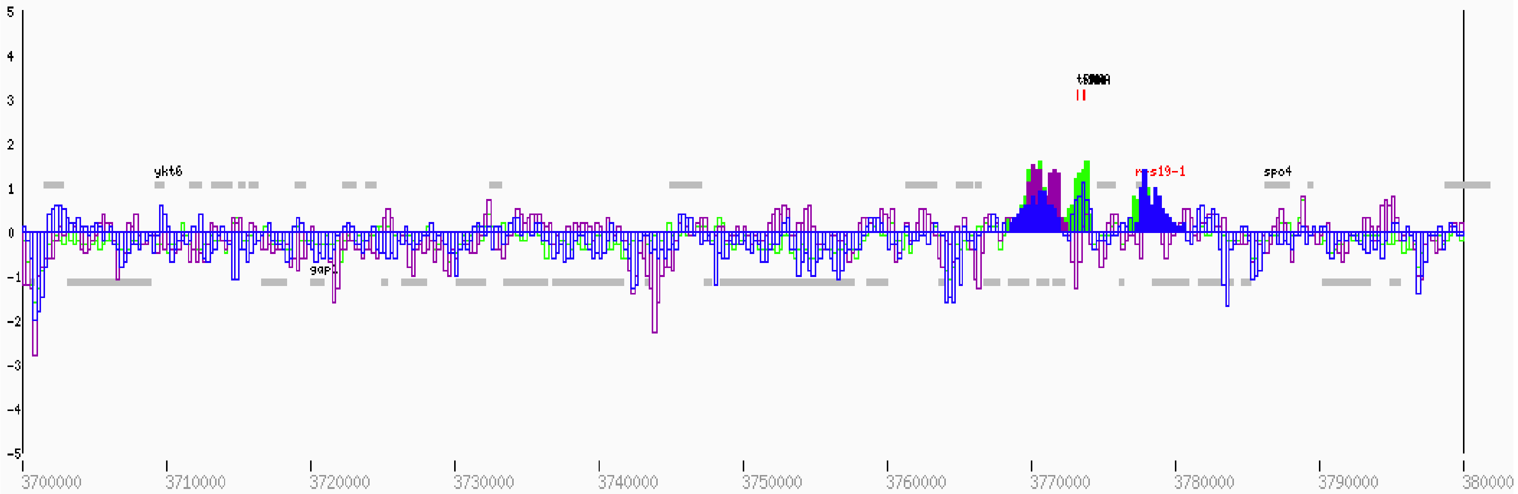


Chromosome 2 (3800000-3900000)

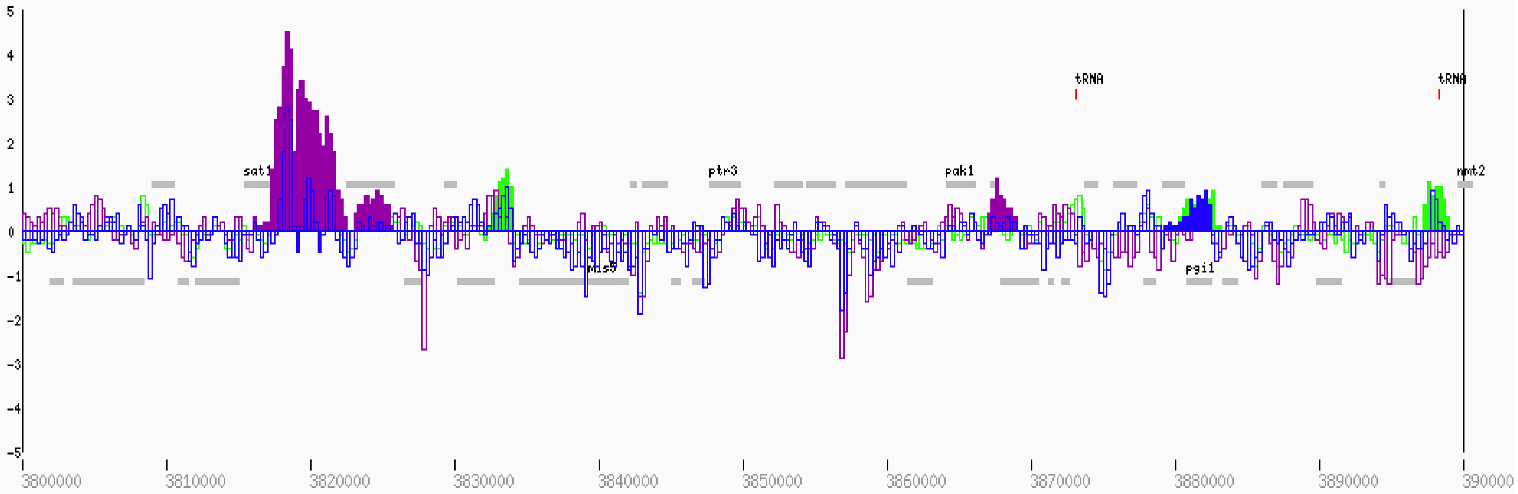


Chromosome 2 (3900000-4000000)

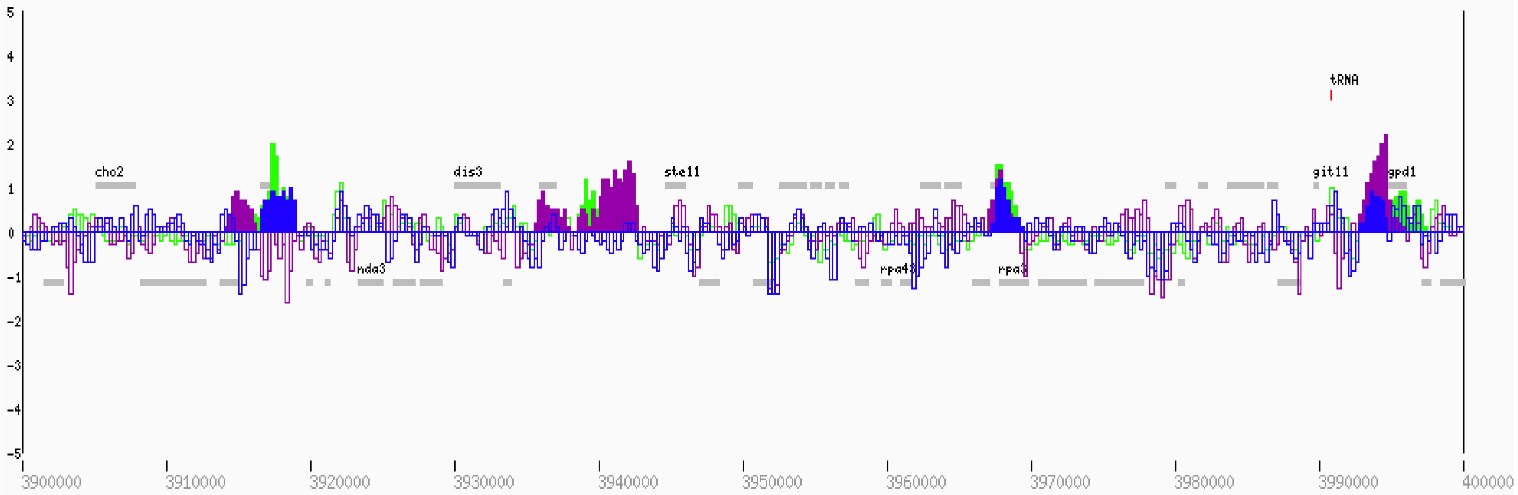

Chromosome 2 (4000000-4100000)

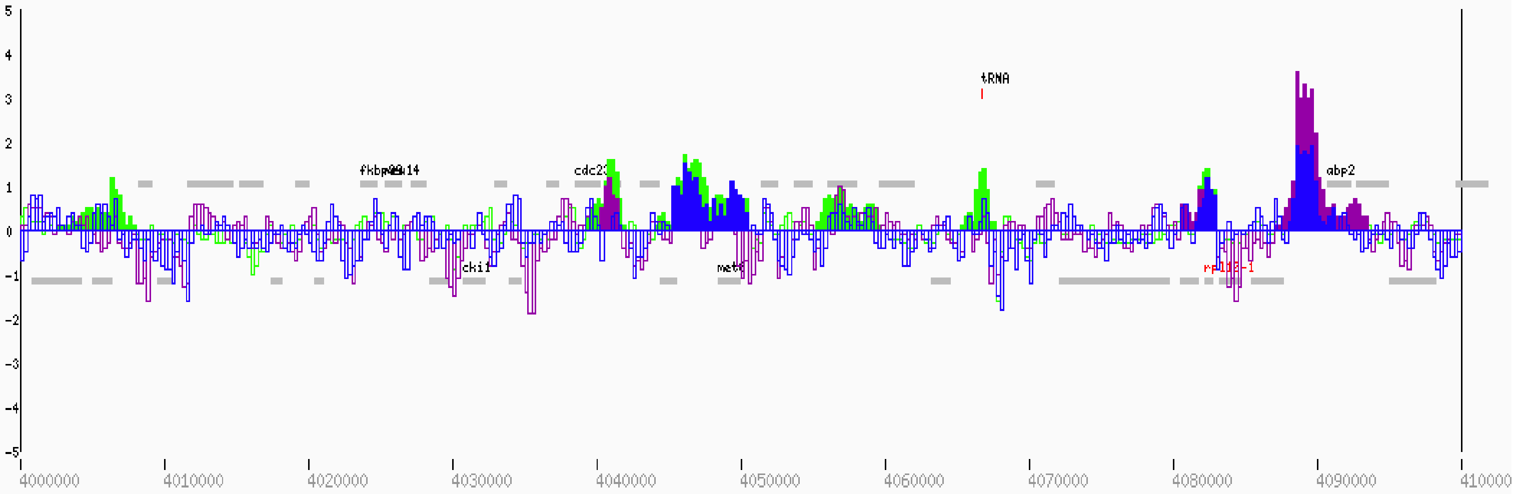


Chromosome 2 (4100000-4200000)

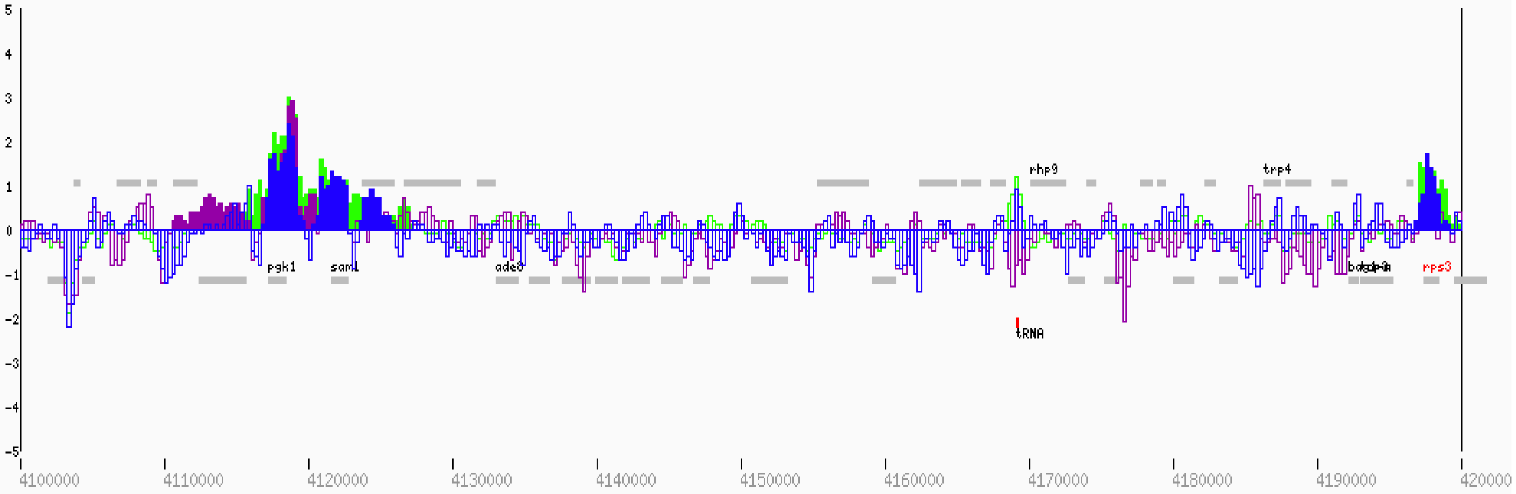


Chromosome 2 (4200000-4300000)

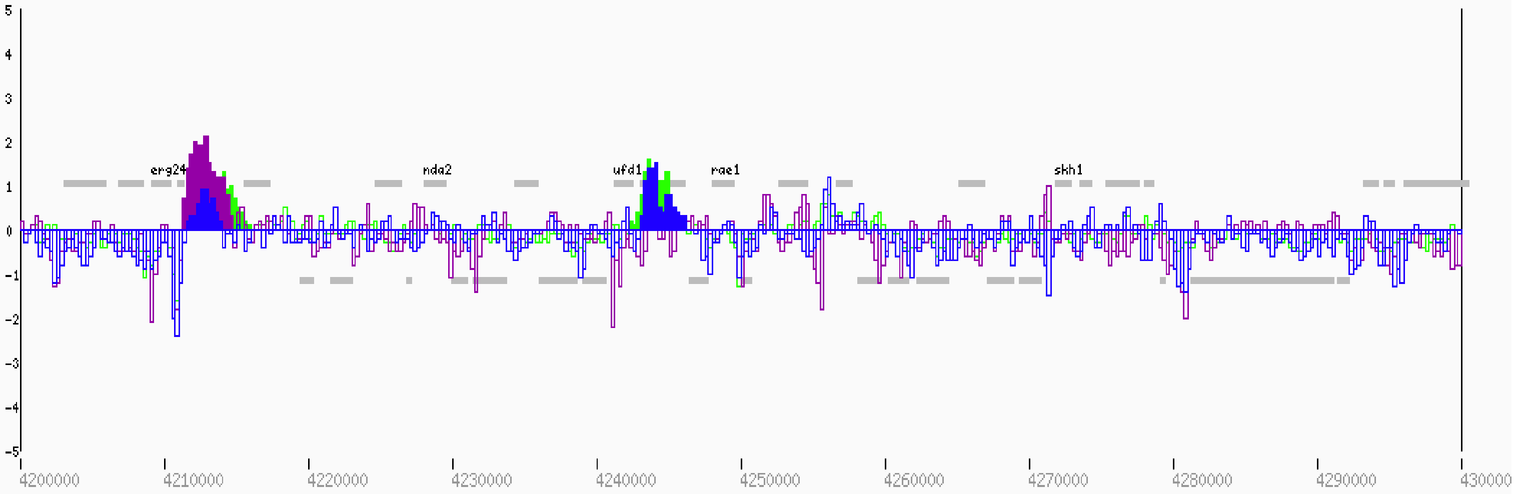


Chromosome 2 (4300000-4400000)

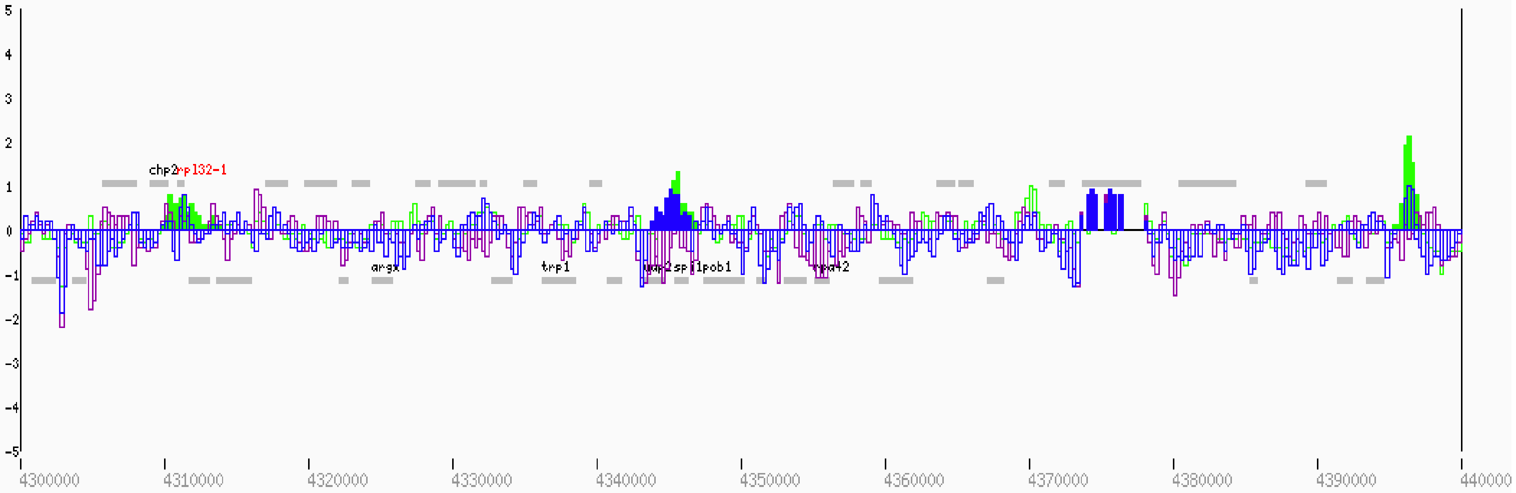

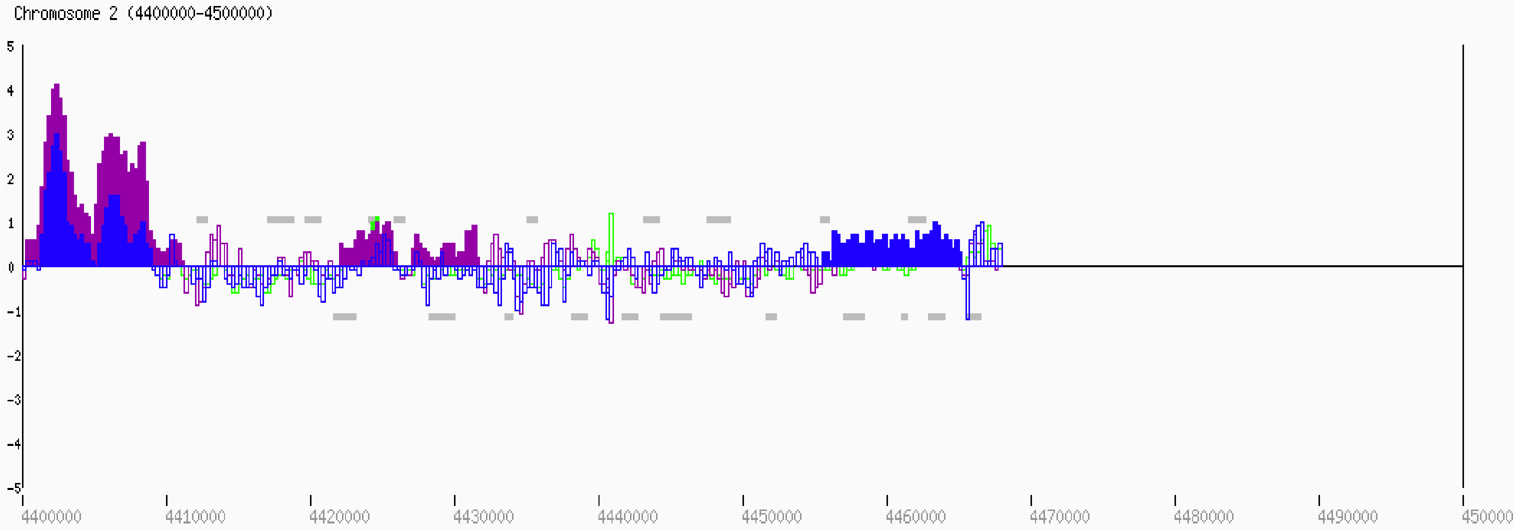

Chromosome 2 (4400000-4500000)
5
4
3
2
1
0
-1
-2
-3
-4
-5
4400000
4410000
4420000
4430000
4440000
4450000
4460000
4470000
4480000
4490000
450000

**(b)**

| Peak No | Midpoint/bp | Avg height | Width/bp |
|---|---|---|---|
| 1 | 12251 | 0,53 | 3500 |
| 2 | 101876 | 0,47 | 3750 |
| 3 | 136001 | 0,55 | 3000 |
| 4 | 178751 | 1,11 | 2500 |
| 5 | 205376 | 0,82 | 1750 |
| 6 | 246126 | 0,75 | 2250 |
| 7 | 332876 | 0,63 | 4250 |
| 8 | 385501 | 0,82 | 4000 |
| 9 | 490001 | 0,60 | 3500 |
| 10 | 496876 | 0,85 | 2250 |
| 11 | 636001 | 0,80 | 4500 |
| 12 | 656376 | 0,48 | 3250 |
| 13 | 675126 | 0,63 | 2750 |
| 14 | 803751 | 0,77 | 2000 |
| 15 | 1001126 | 0,71 | 4750 |
| 16 | 1157376 | 0,50 | 4750 |
| 17 | 1298626 | 0,60 | 3250 |
| 18 | 1462001 | 0,53 | 3000 |
| 19 | 1471626 | 0,83 | 1750 |
| 20 | 1505376 | 0,73 | 3250 |
| 21 | 1516376 | 0,88 | 2750 |
| 22 | 1610001 | 0,63 | 3000 |
| 23 | 1667001 | 0,74 | 3000 |
| 24 | 1686376 | 0,66 | 2250 |
| 25 | 1692626 | 0,86 | 2250 |
| 26 | 1734501 | 0,64 | 2500 |
| 27 | 1751626 | 1,16 | 5250 |
| 28 | 1785376 | 0,90 | 2750 |
| 29 | 1847001 | 0,77 | 2500 |
| 30 | 2005001 | 1,18 | 2500 |
| 31 | 2019626 | 0,63 | 2250 |
| 32 | 2042001 | 0,97 | 5000 |
| 33 | 2065001 | 0,51 | 3000 |
| 34 | 2073501 | 0,90 | 2500 |
| 35 | 2080876 | 0,68 | 2250 |
| 36 | 2091251 | 0,96 | 11500 |

| Peak No | Midpoint/bp | Avg height | Width/bp |
|---|---|---|---|
| 37 | 2104126 | 0,68 | 10750 |
| 38 | 2121626 | 0,58 | 3250 |
| 39 | 2140876 | 0,61 | 2750 |
| 40 | 2249501 | 0,64 | 2500 |
| 41 | 2278126 | 0,85 | 1750 |
| 42 | 2307751 | 1,03 | 2000 |
| 43 | 2338751 | 0,57 | 2500 |
| 44 | 2375626 | 0,58 | 2750 |
| 45 | 2408001 | 0,56 | 3000 |
| 46 | 2546876 | 0,73 | 8250 |
| 47 | 2738876 | 0,92 | 3250 |
| 48 | 2857376 | 0,53 | 3750 |
| 49 | 2899126 | 0,63 | 2750 |
| 50 | 2979376 | 1,00 | 1750 |
| 51 | 3113751 | 0,86 | 3500 |
| 52 | 3132376 | 0,73 | 2750 |
| 53 | 3204626 | 0,72 | 2250 |
| 54 | 3234751 | 0,45 | 3500 |
| 55 | 3322126 | 0,73 | 6750 |
| 56 | 3351751 | 0,64 | 3500 |
| 57 | 3465501 | 0,59 | 4000 |
| 58 | 3613251 | 0,55 | 3000 |
| 59 | 3777751 | 0,70 | 2500 |
| 60 | 3833126 | 0,98 | 1750 |
| 61 | 3881376 | 0,54 | 2750 |
| 62 | 3898001 | 0,79 | 2000 |
| 63 | 3916876 | 0,79 | 3750 |
| 64 | 4005251 | 0,51 | 4000 |
| 65 | 4047751 | 0,94 | 5500 |
| 66 | 4057251 | 0,58 | 4500 |
| 67 | 4066501 | 0,64 | 2500 |
| 68 | 4197876 | 1,02 | 2750 |
| 69 | 4243876 | 1,11 | 2750 |
| 70 | 4311126 | 0,55 | 2750 |
| 71 | 4345626 | 0,64 | 2750 |
| 72 | 4396376 | 1,13 | 1750 |

(c)

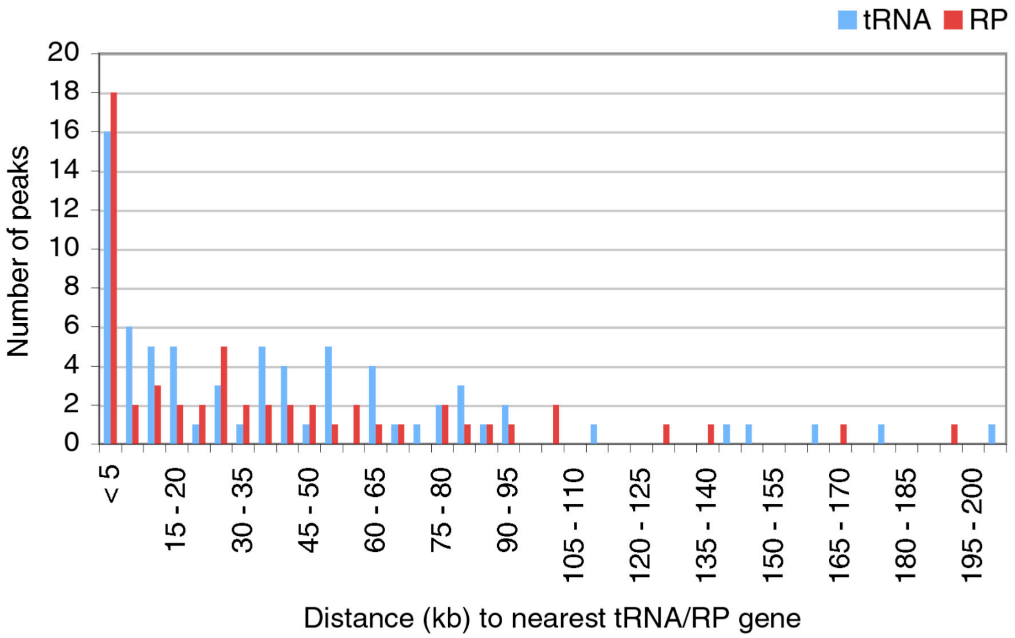
tRNA
RP
Number of peaks
0
2
4
6
8
10
12
14
16
18
20
< 5
15 - 20
30 - 35
45 - 50
60 - 65
75 - 80
90 - 95
105 - 110
120 - 125
135 - 140
150 - 155
165 - 170
180 - 185
195 - 200
Distance (kb) to nearest tRNA/RP gene

**Figure S5**

Similar Mis4 localization in exponentially growing and in G1 arrested cells. The Mis4 localization pattern along chromosome 2 in exponentially growing cells is compared to the pattern observed in cells arrested in G1 using the *cdc10-129* temperature sensitive mutation. (**a**) Exponentially growing *cdc10-129* cells were shifted to the restrictive temperature of 37°C for 3.5 hours. G1 arrest was confirmed by flow cytometry of DNA content. (**b**) Chromatin immunoprecipitation was performed against epitope-tagged Mis4-$Pk_9$ from the G1 arrested cells 3.5 hours after temperature shift. The association pattern along chromosome 2 is shown (brown) in comparison to that observed in exponentially growing cells that are predominantly in the G2 phase of the cell cycle (geen, data reproduced from Figure S4).

**(a)**

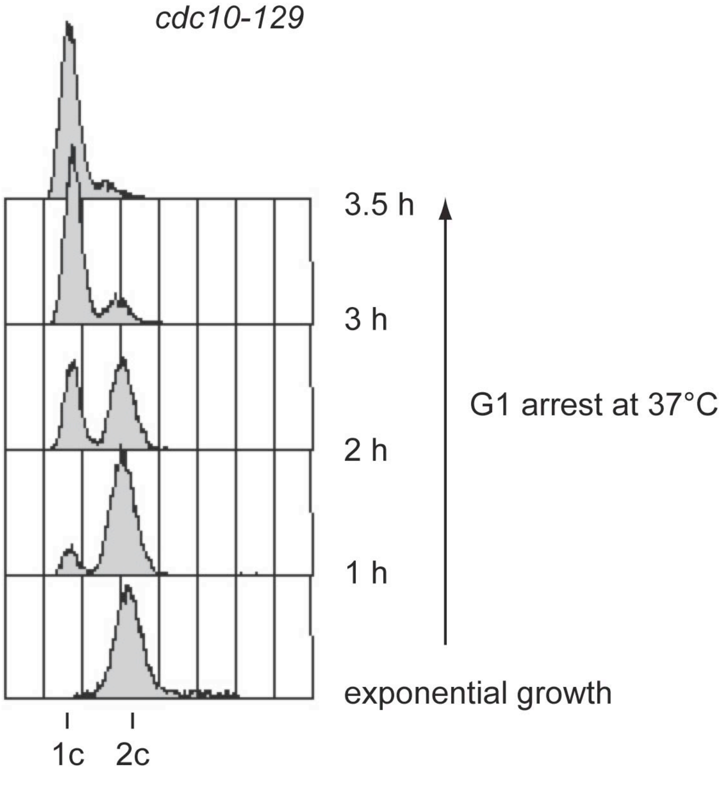

(b)

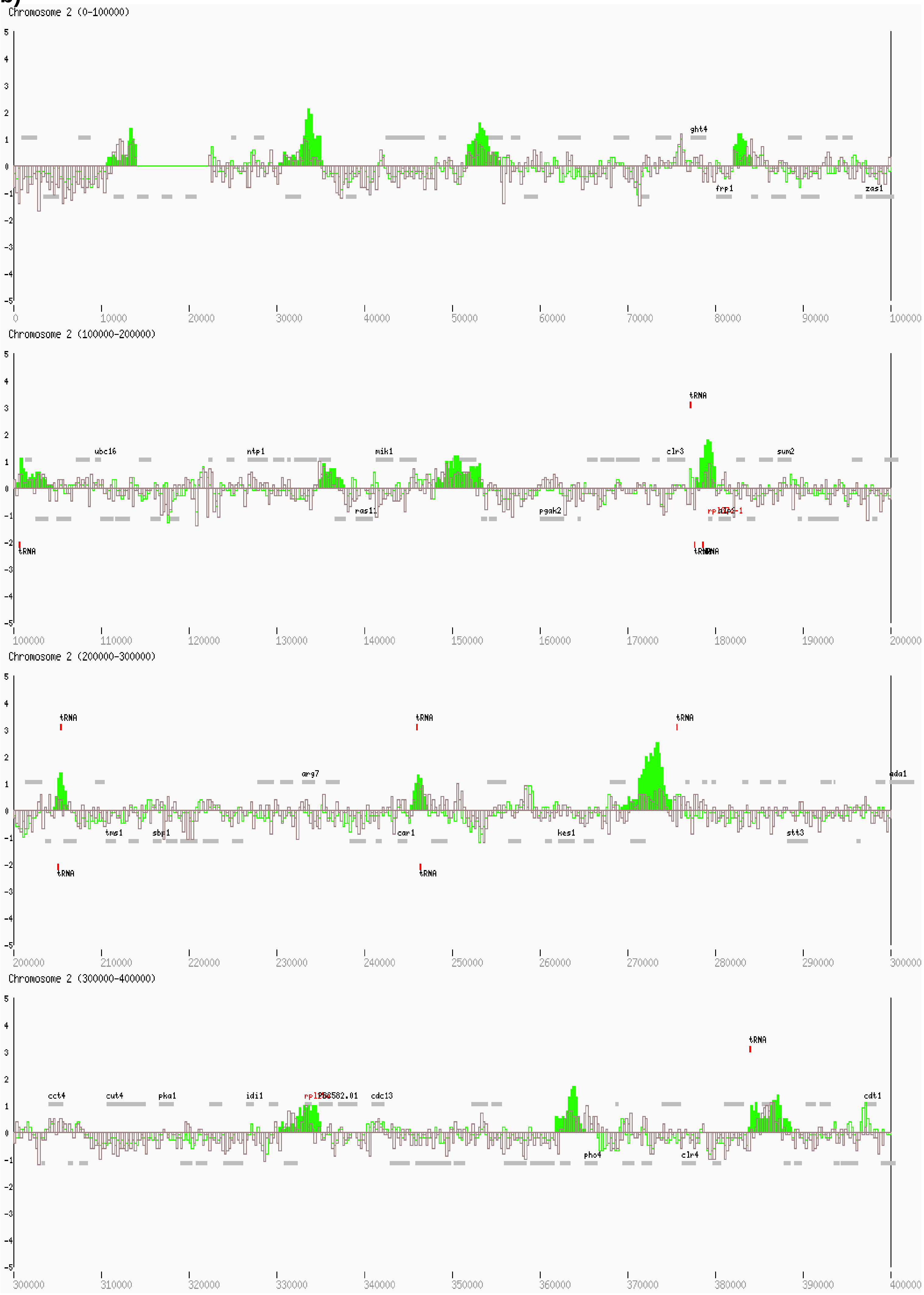
Chromosome 2 (0-100000)
ght4
frp1
zas1
Chromosome 2 (100000-200000)
tRNA
ubc16
ntp1
mik1
clr3
sum2
ras1
pgak2
Chromosome 2 (200000-300000)
arg7
ada1
tms1
car1
kes1
stt3
Chromosome 2 (300000-400000)
cct4
cut4
pka1
idi1
cdc13
cdt1
pho4
clr4

Chromosome 2 (400000-500000)

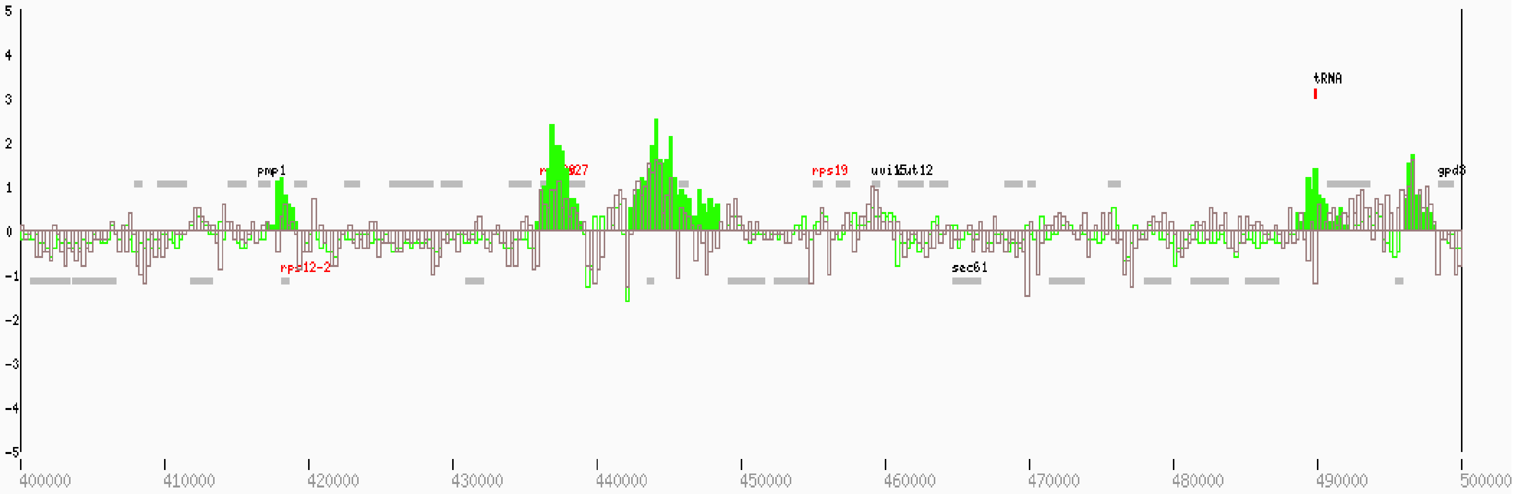


Chromosome 2 (500000-600000)

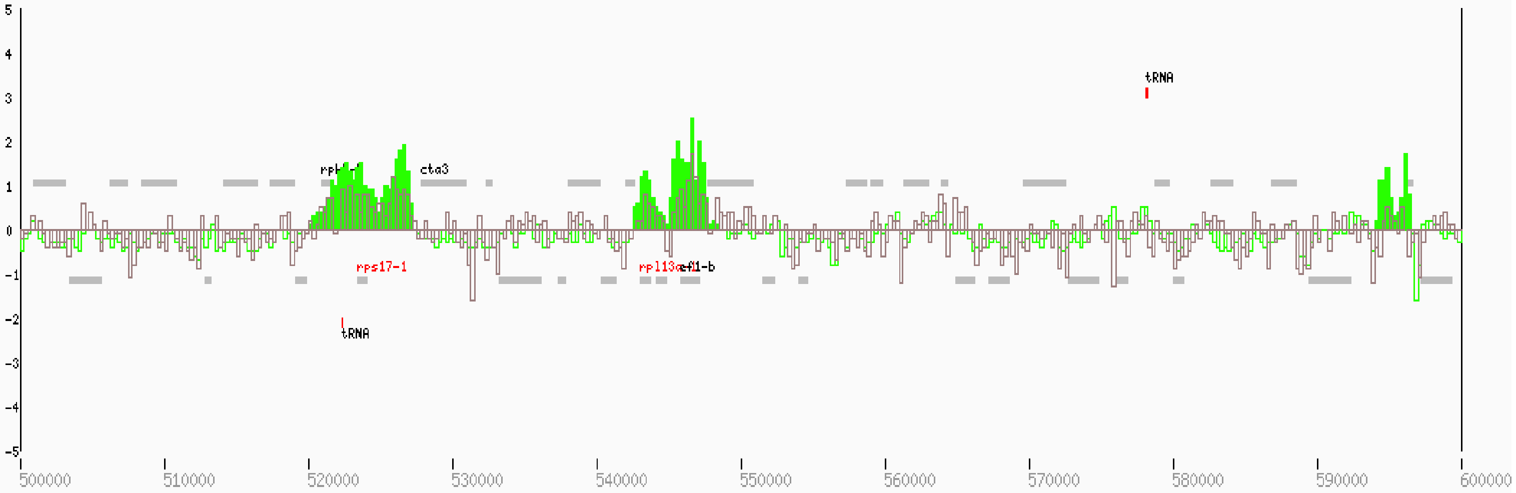


Chromosome 2 (600000-700000)

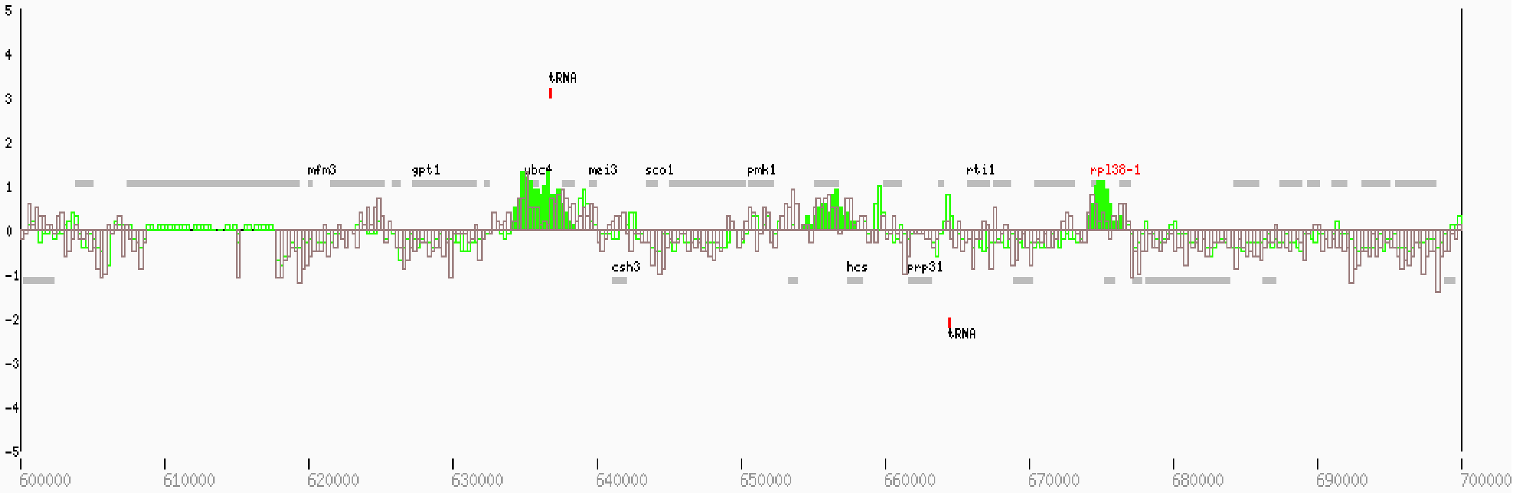


Chromosome 2 (700000-800000)

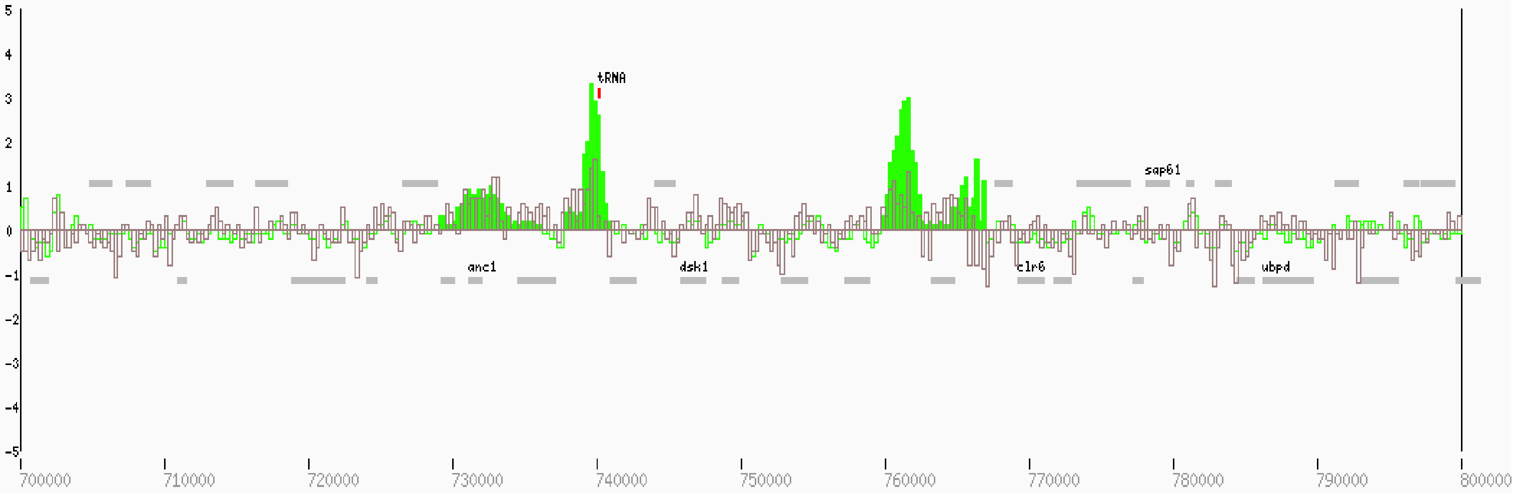

Chromosome 2 (800000-900000)

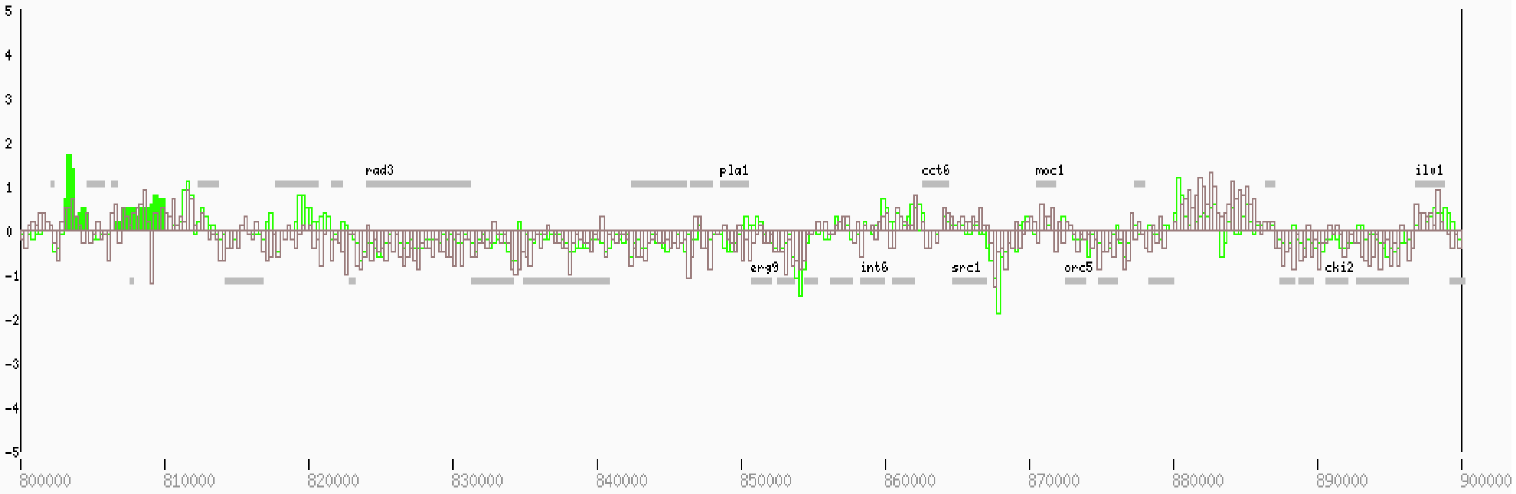


Chromosome 2 (900000-1000000)

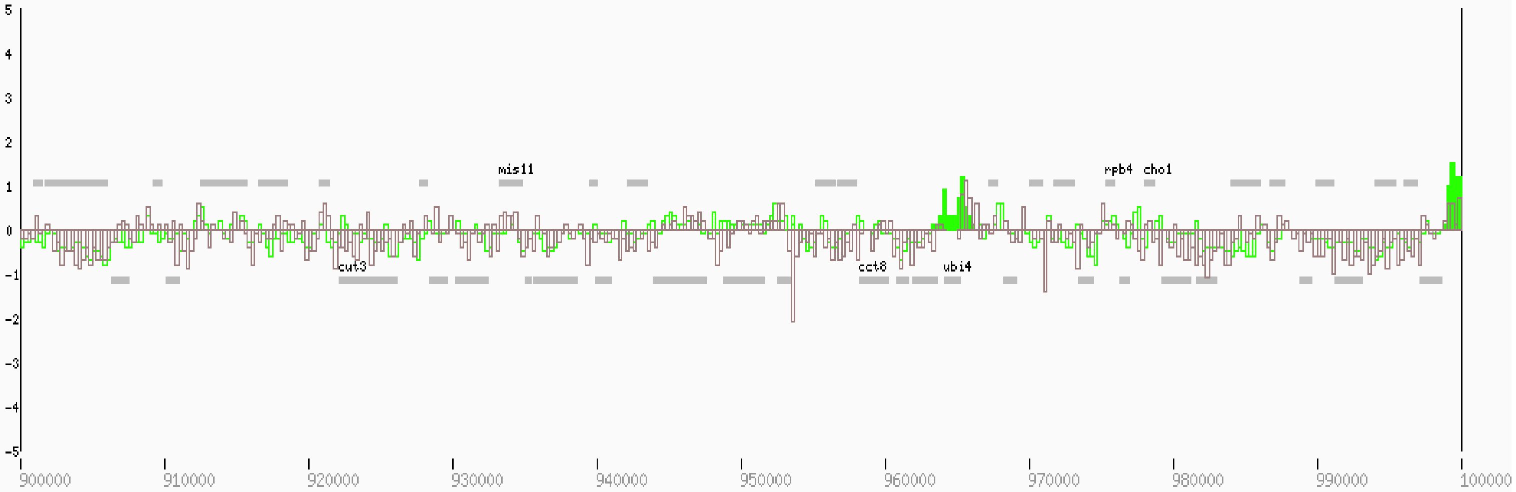


Chromosome 2 (1000000-1100000)

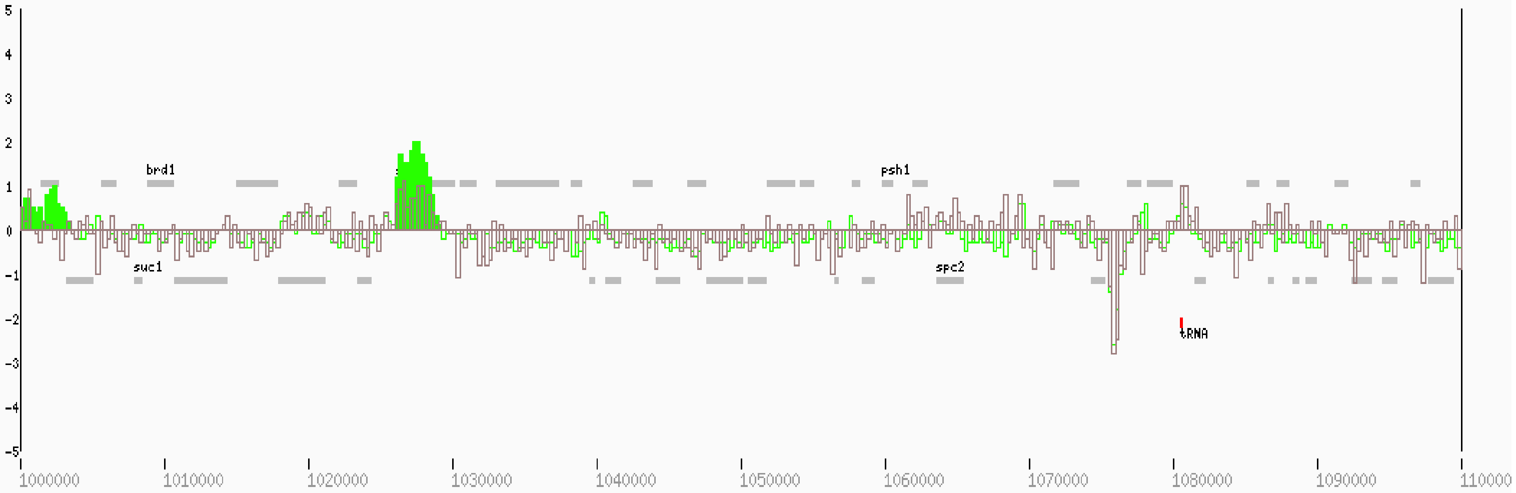


Chromosome 2 (1100000-1200000)

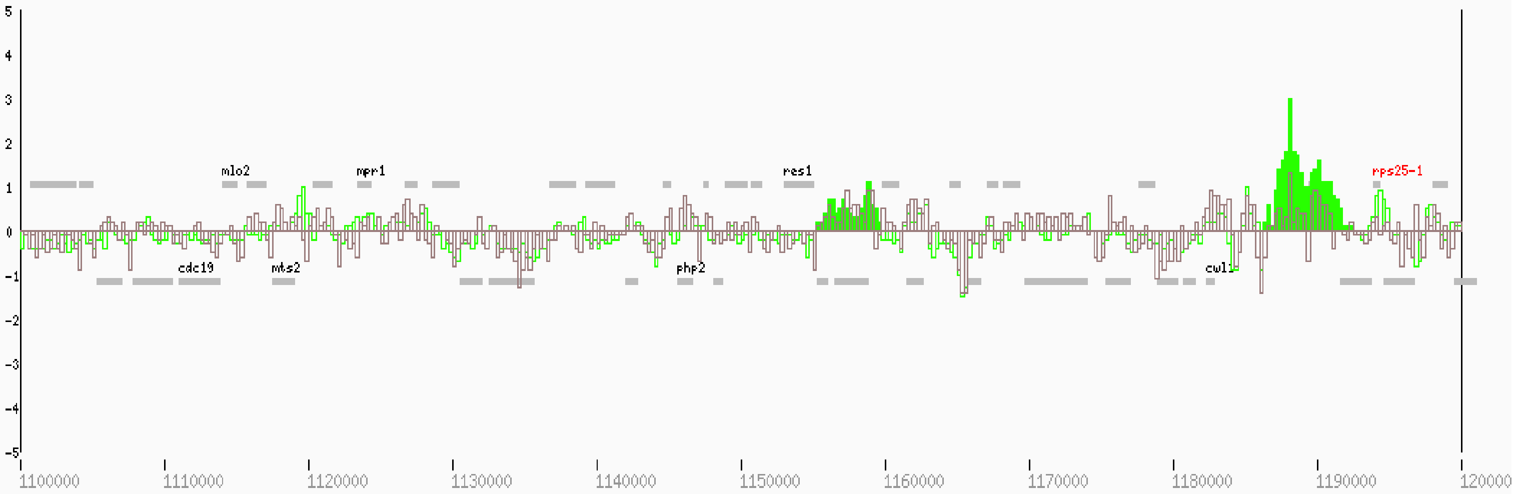

Chromosome 2 (1200000-1300000)

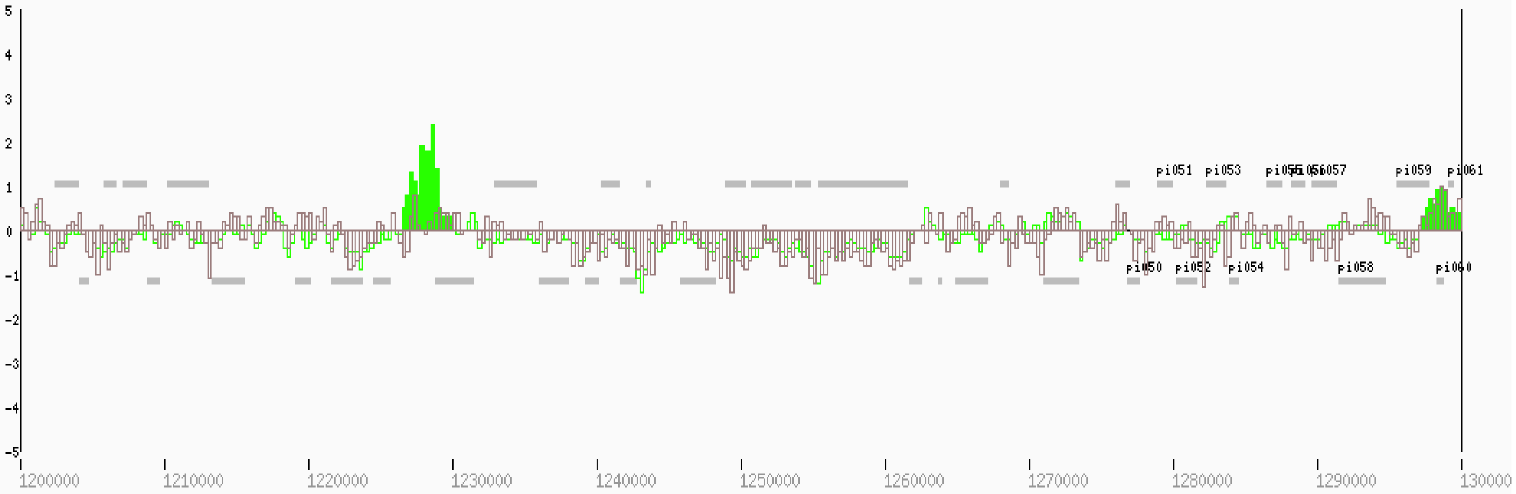


Chromosome 2 (1300000-1400000)

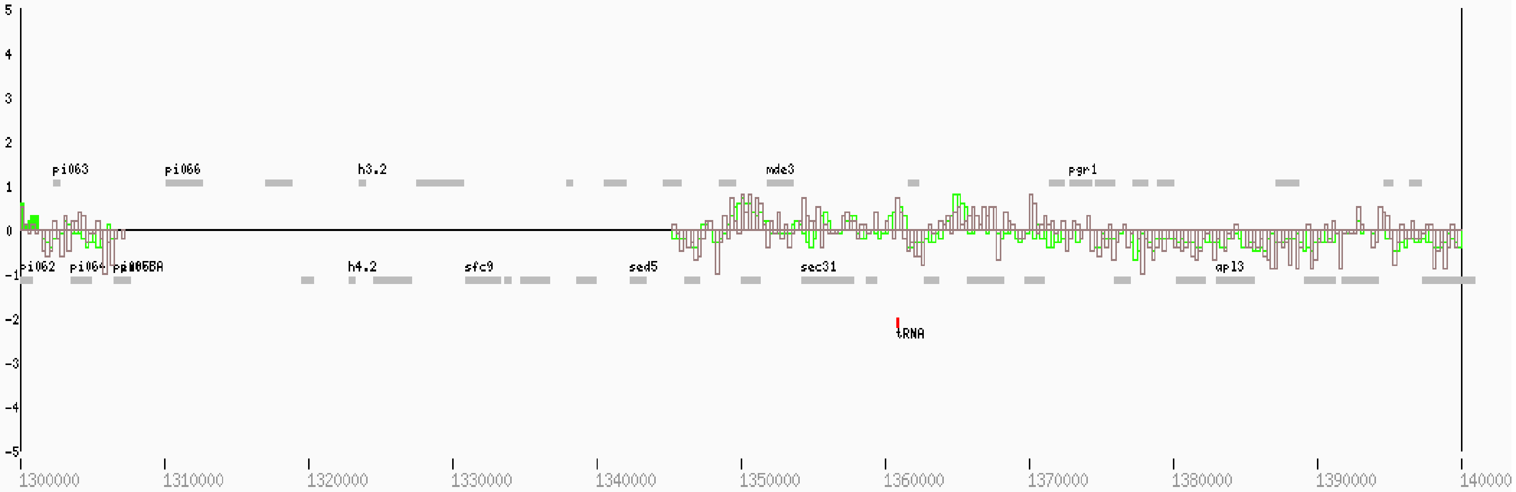


Chromosome 2 (1400000-1500000)

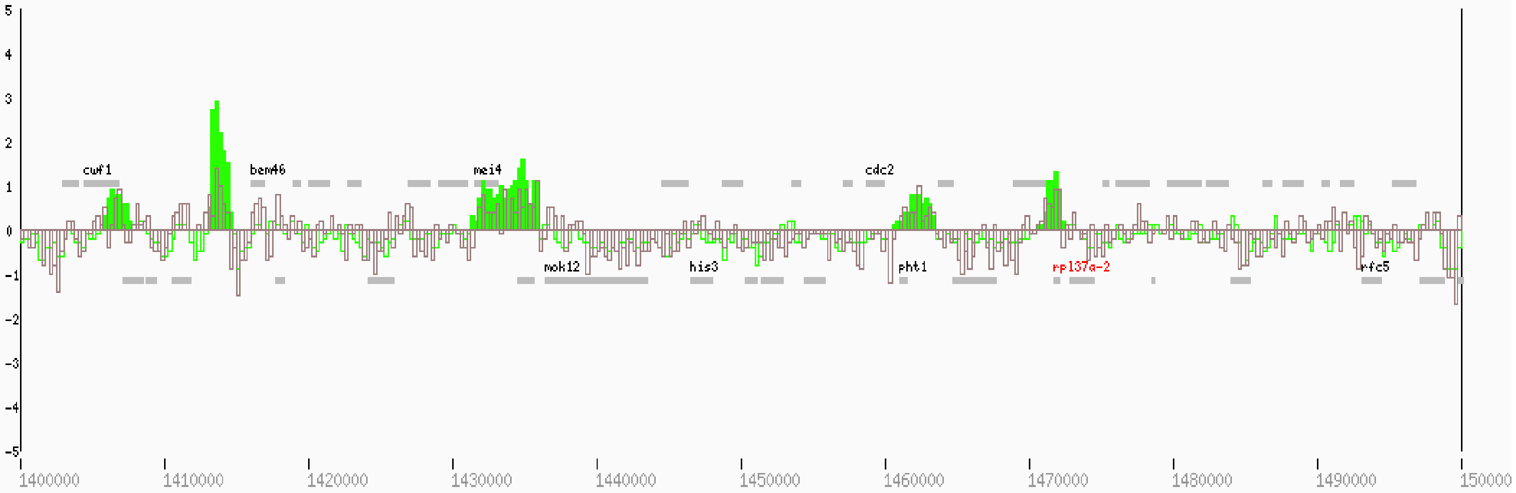


Chromosome 2 (1500000-1600000)

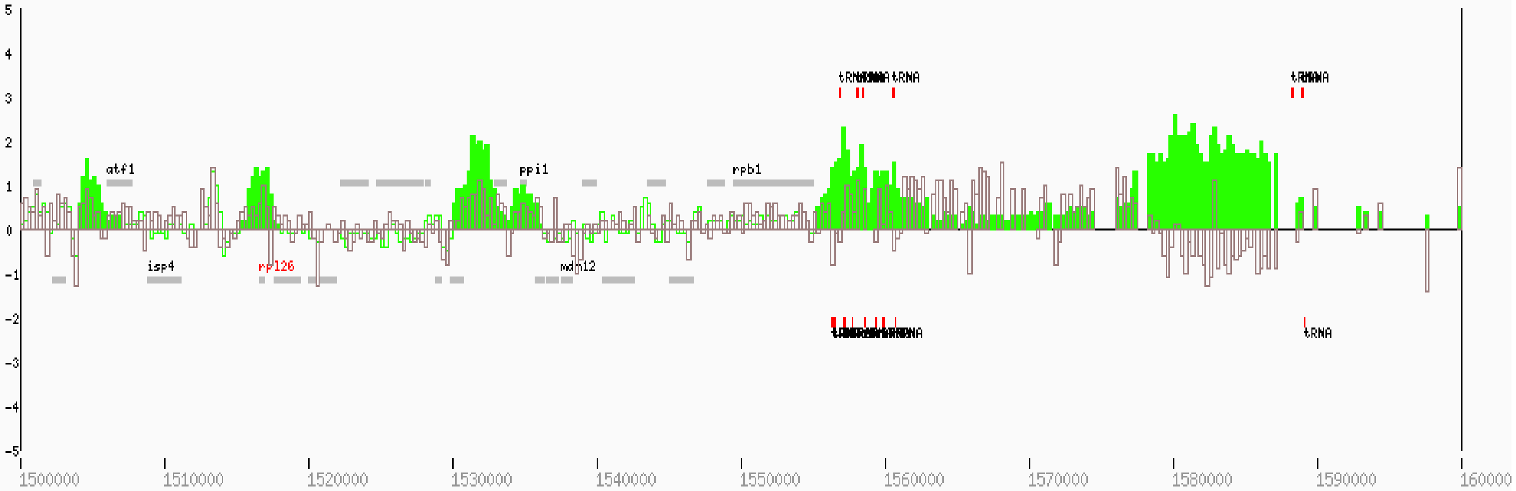

Chromosome 2 (1600000-1700000)

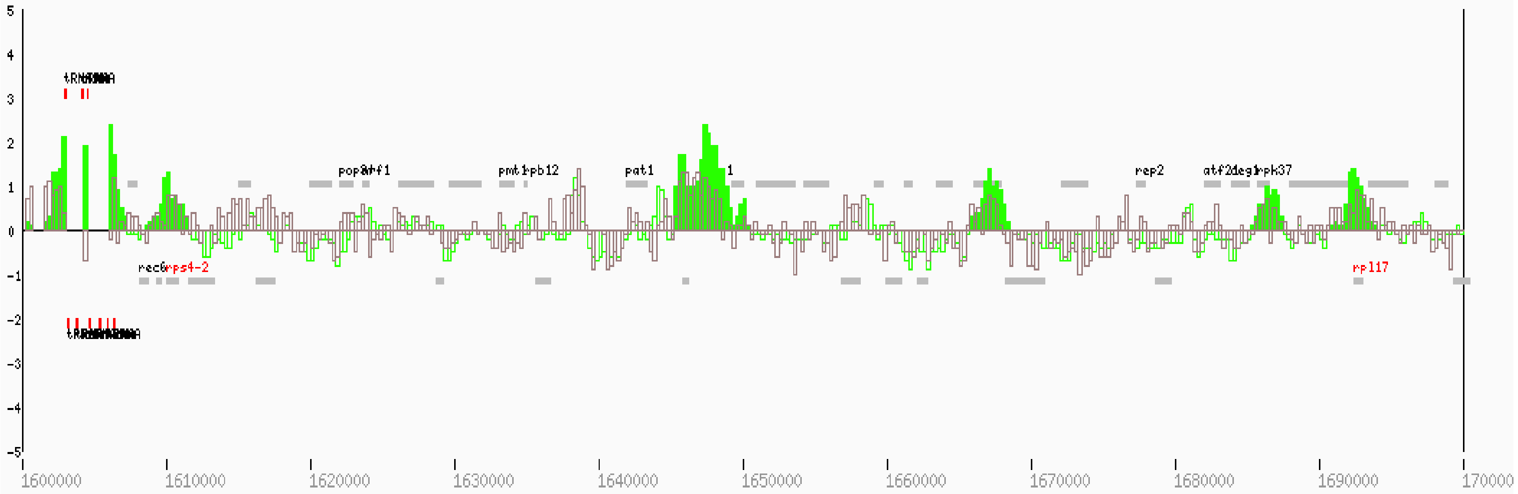


Chromosome 2 (1700000-1800000)

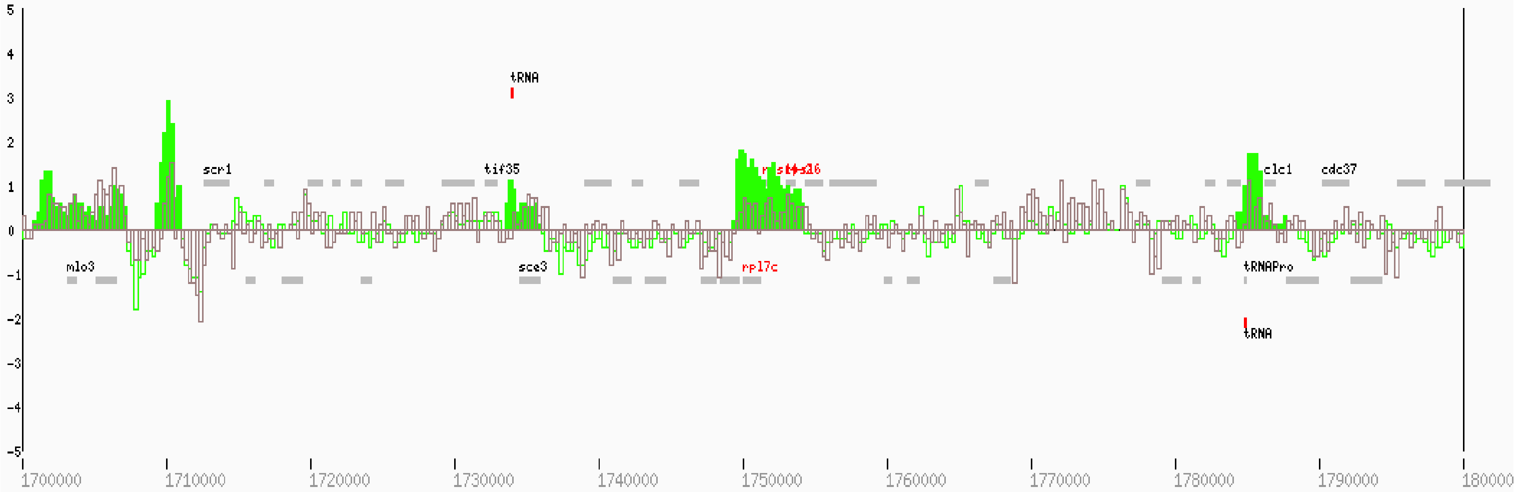


Chromosome 2 (1800000-1900000)

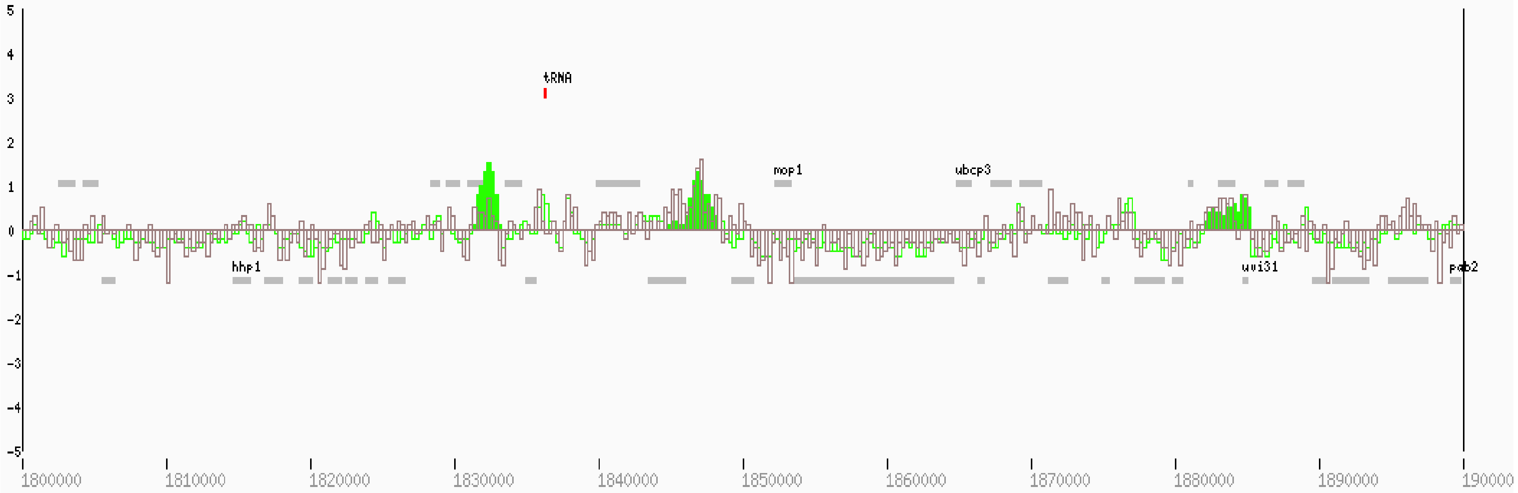


Chromosome 2 (1900000-2000000)

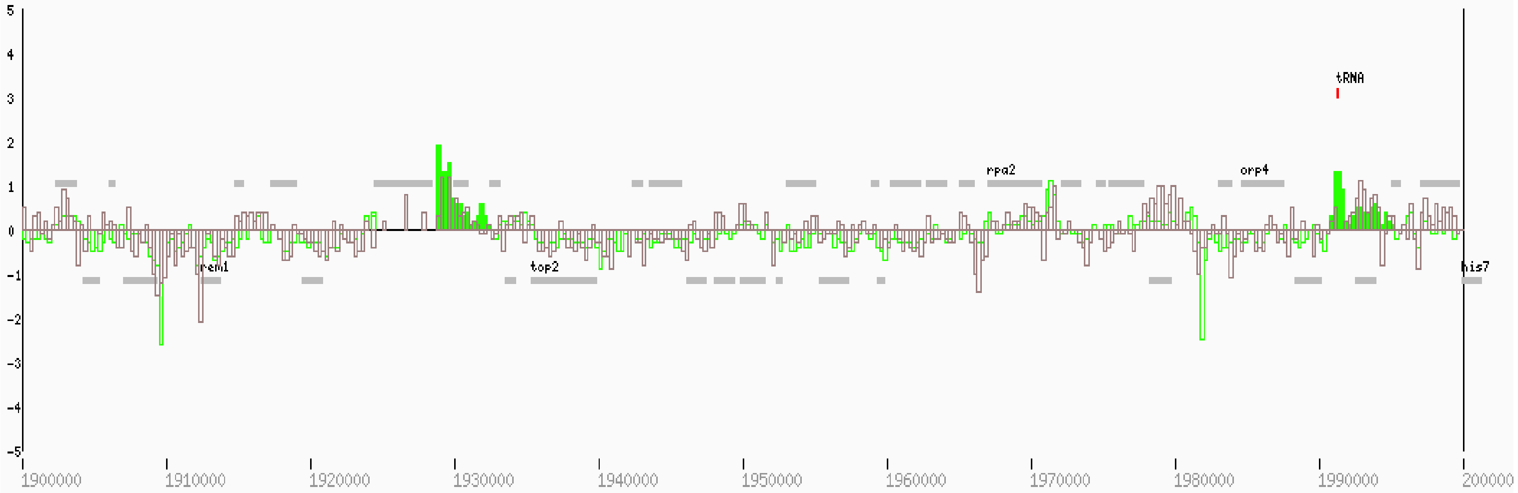

Chromosome 2 (2000000-2100000)

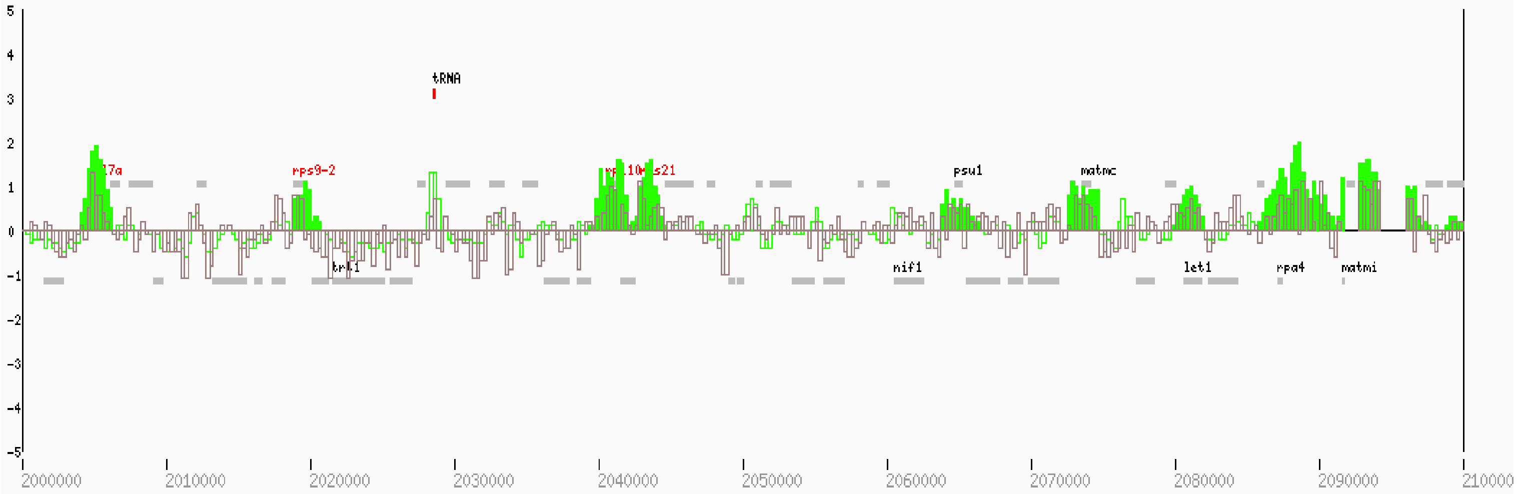


Chromosome 2 (2100000-2200000)

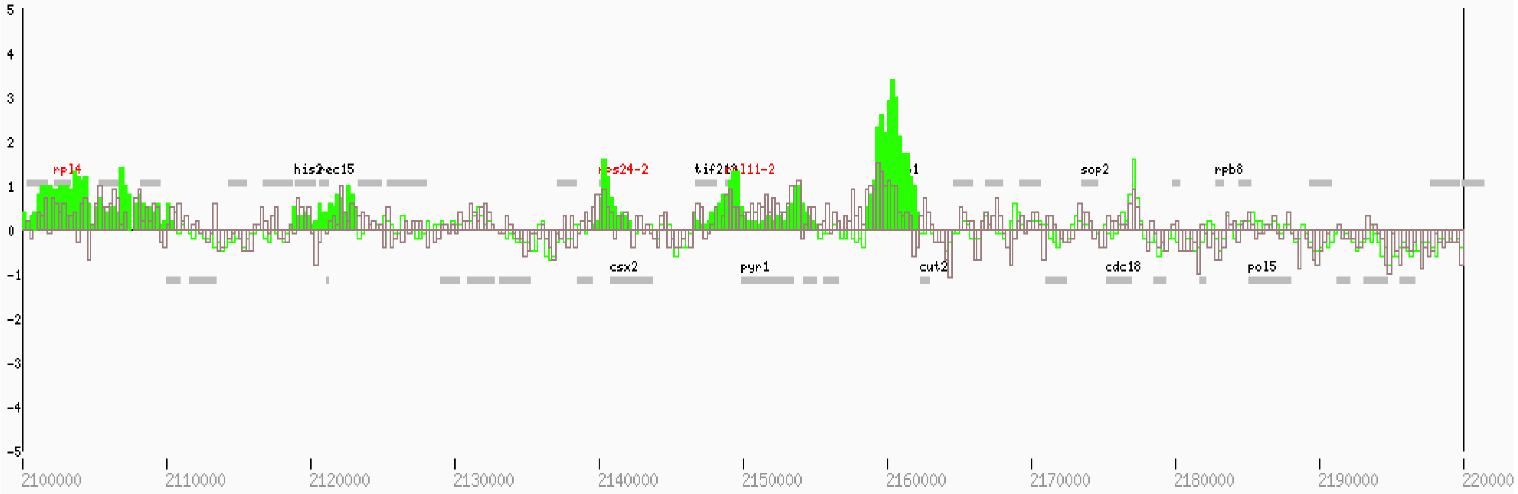


Chromosome 2 (2200000-2300000)

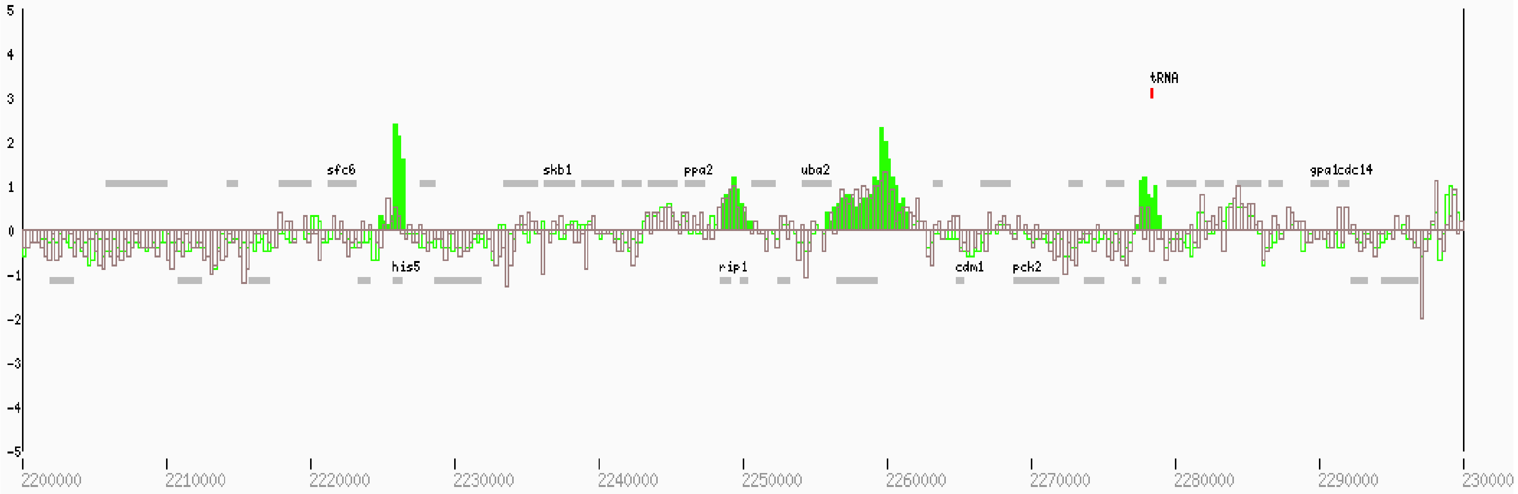


Chromosome 2 (2300000-2400000)

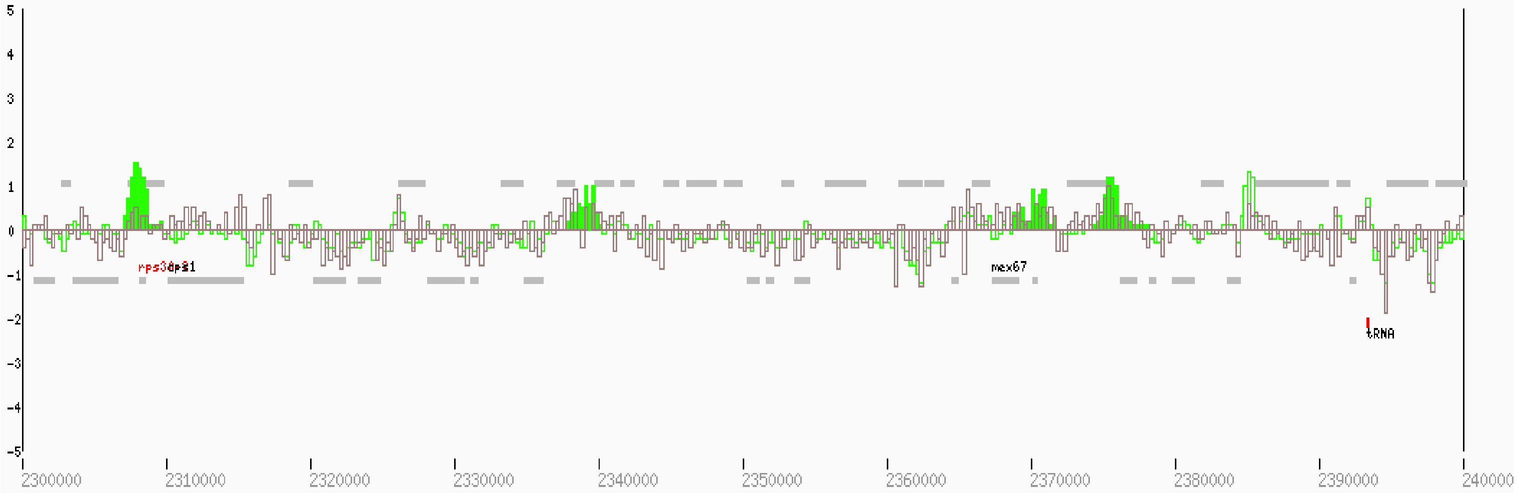

Chromosome 2 (2400000-2500000)

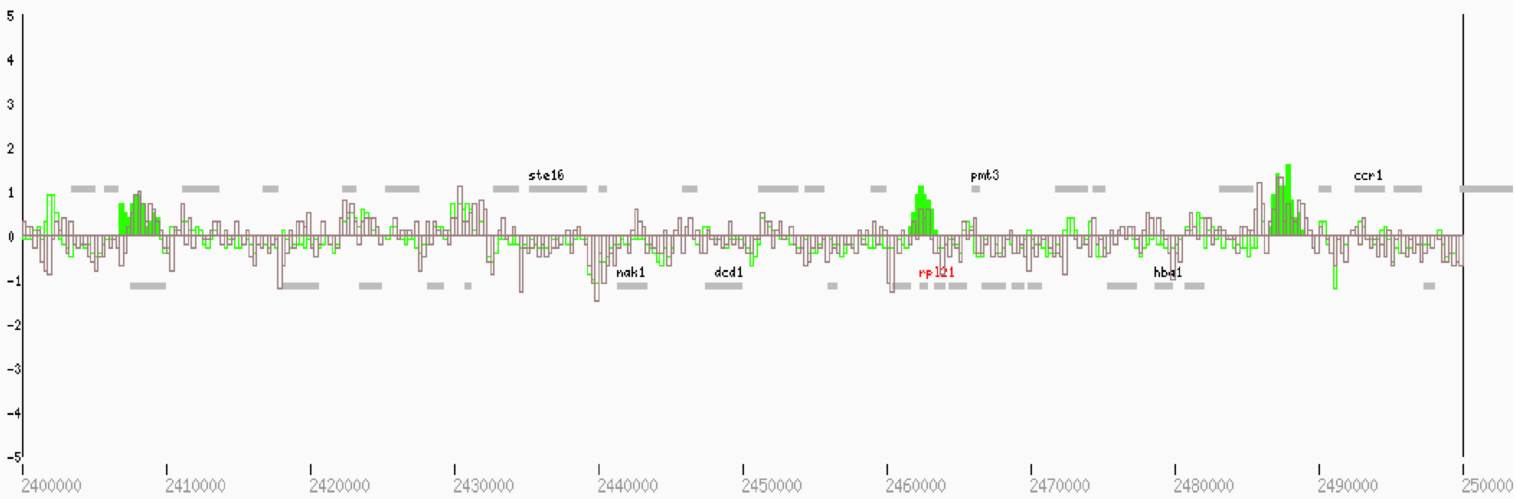


Chromosome 2 (2500000-2600000)

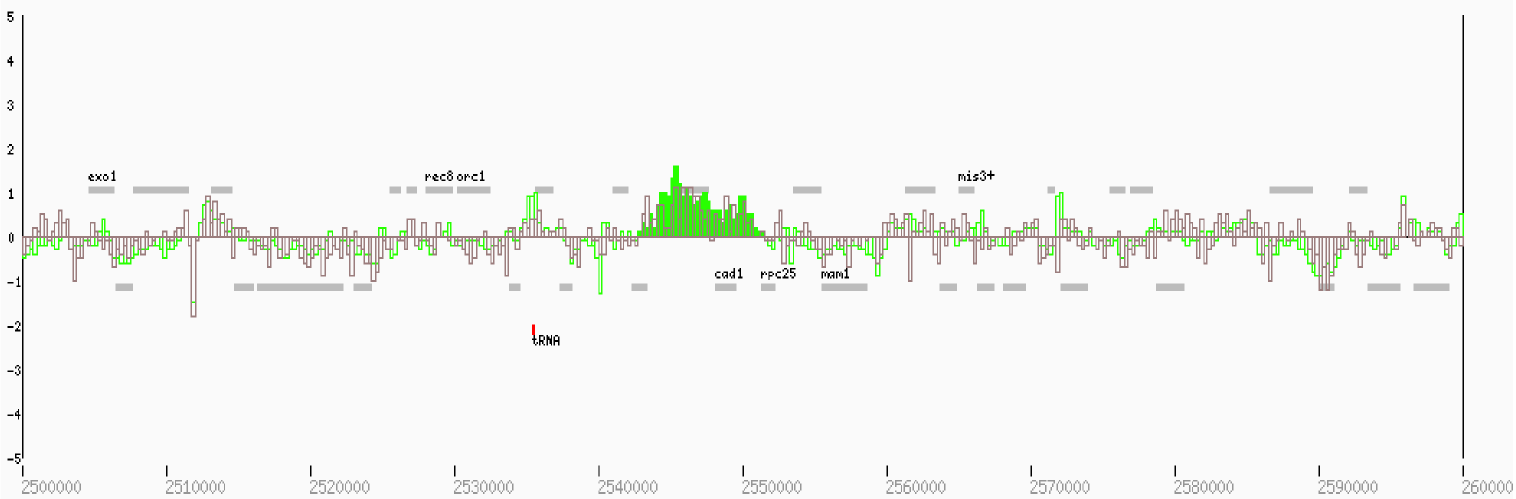


Chromosome 2 (2600000-2700000)

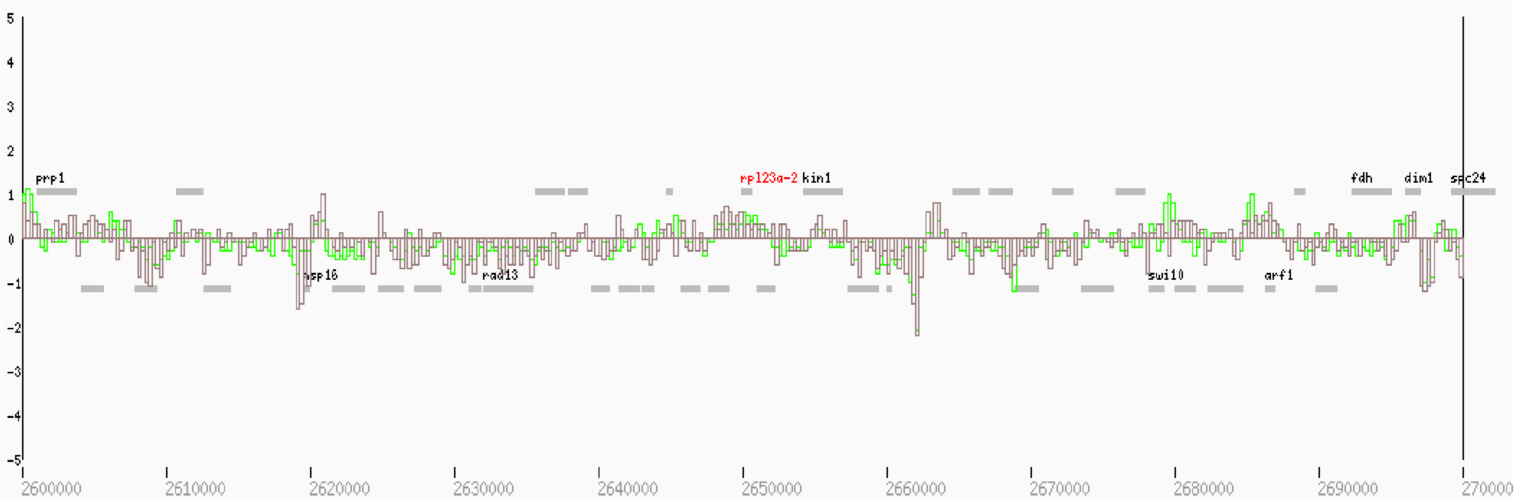


Chromosome 2 (2700000-2800000)

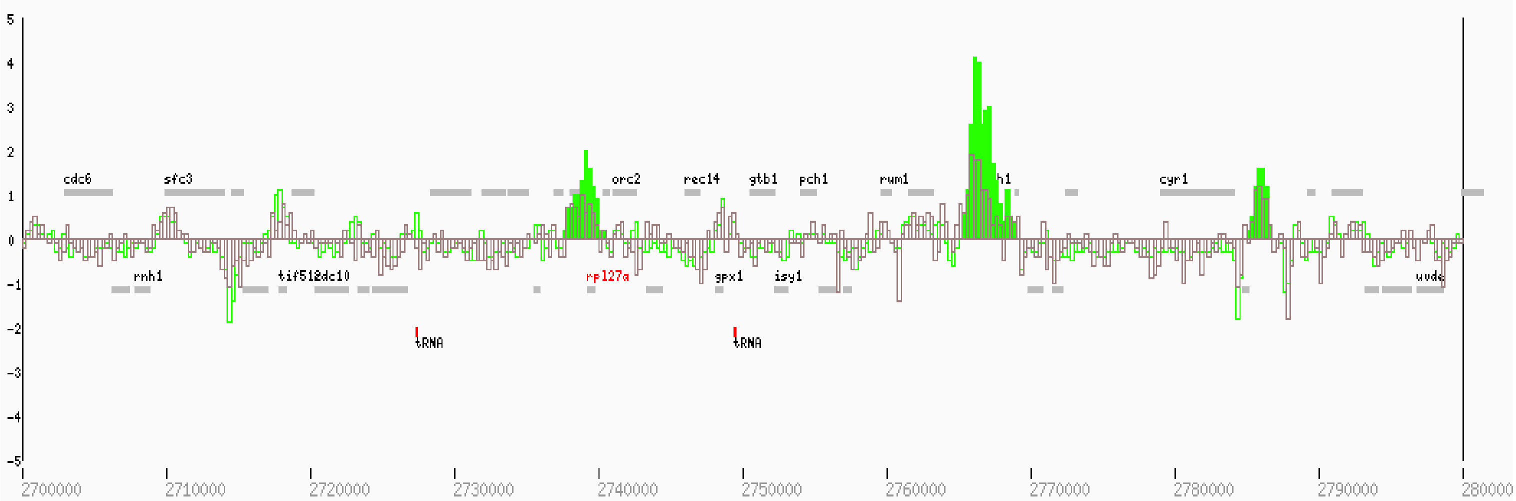

Chromosome 2 (2800000-2900000)

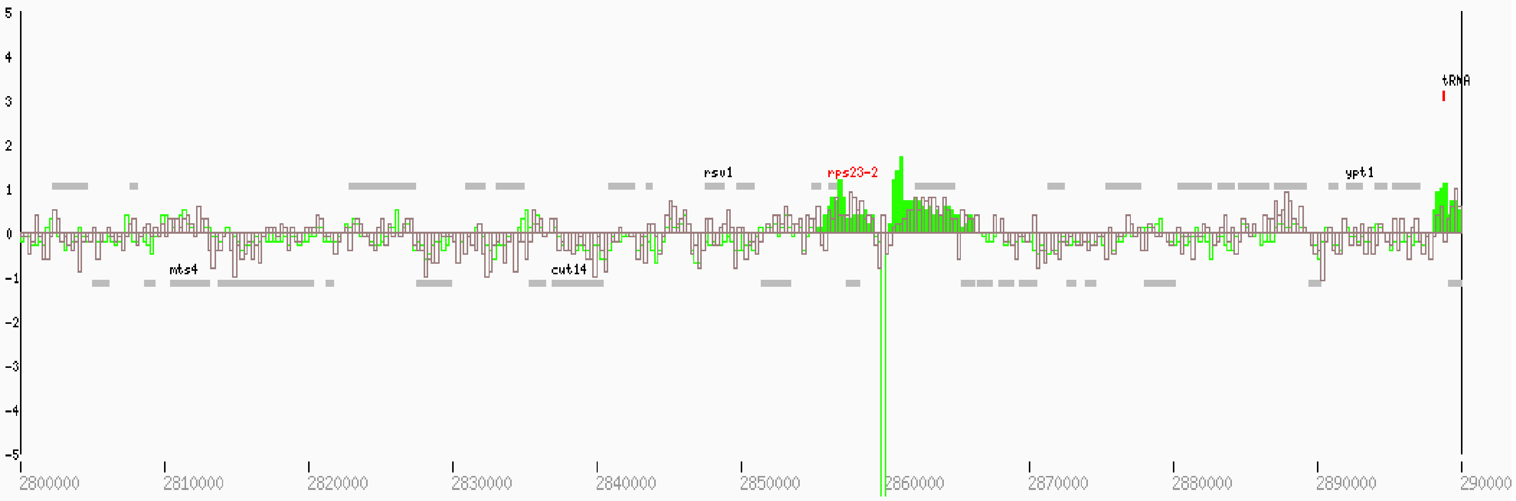


Chromosome 2 (2900000-3000000)

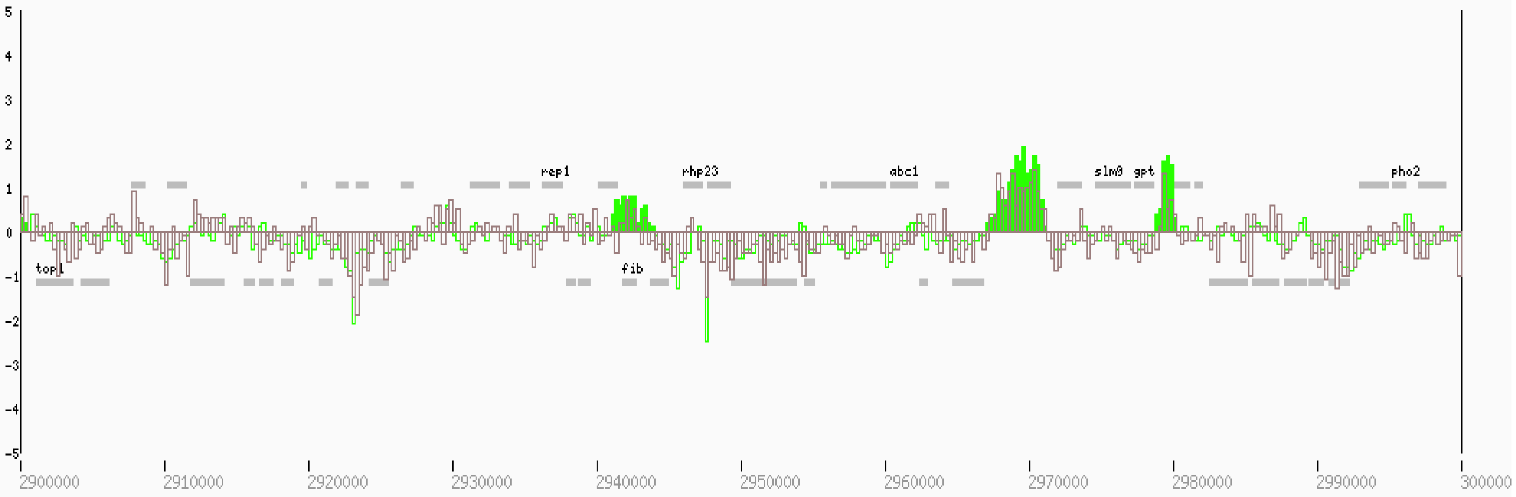


Chromosome 2 (3000000-3100000)

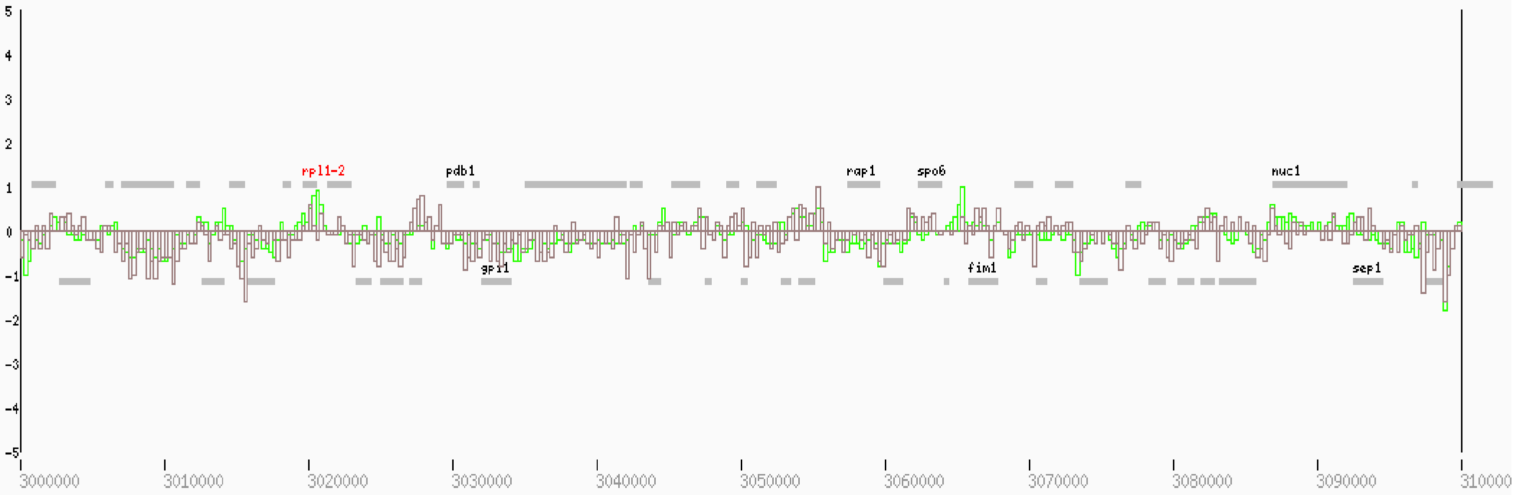


Chromosome 2 (3100000-3200000)

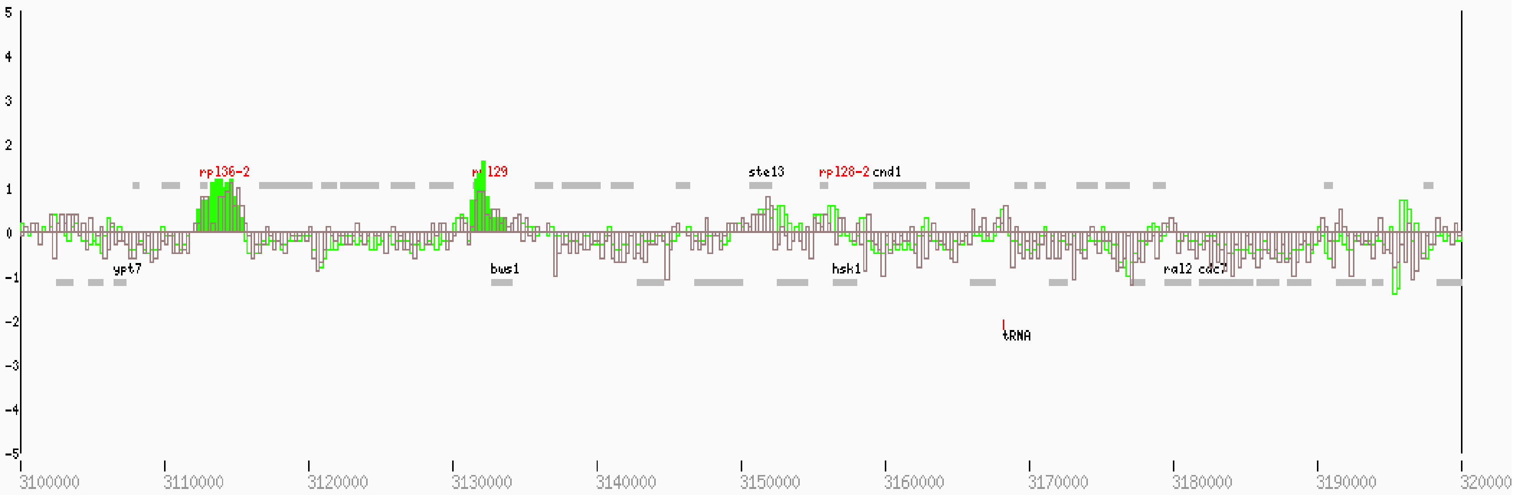

Chromosome 2 (3200000-3300000)

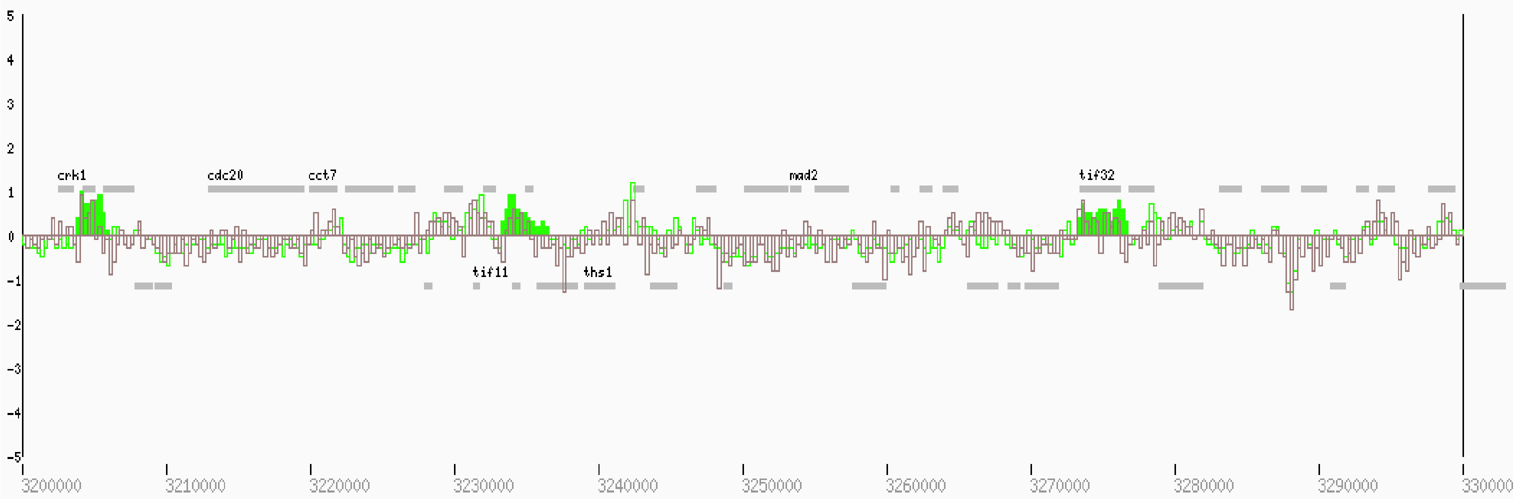


Chromosome 2 (3300000-3400000)

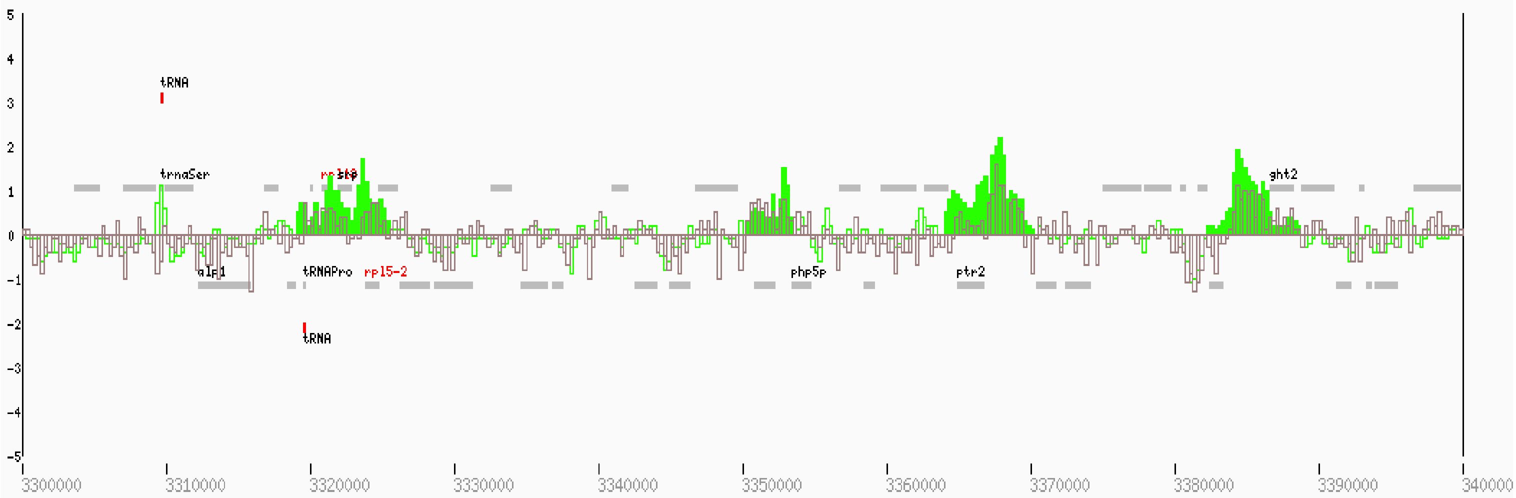


Chromosome 2 (3400000-3500000)

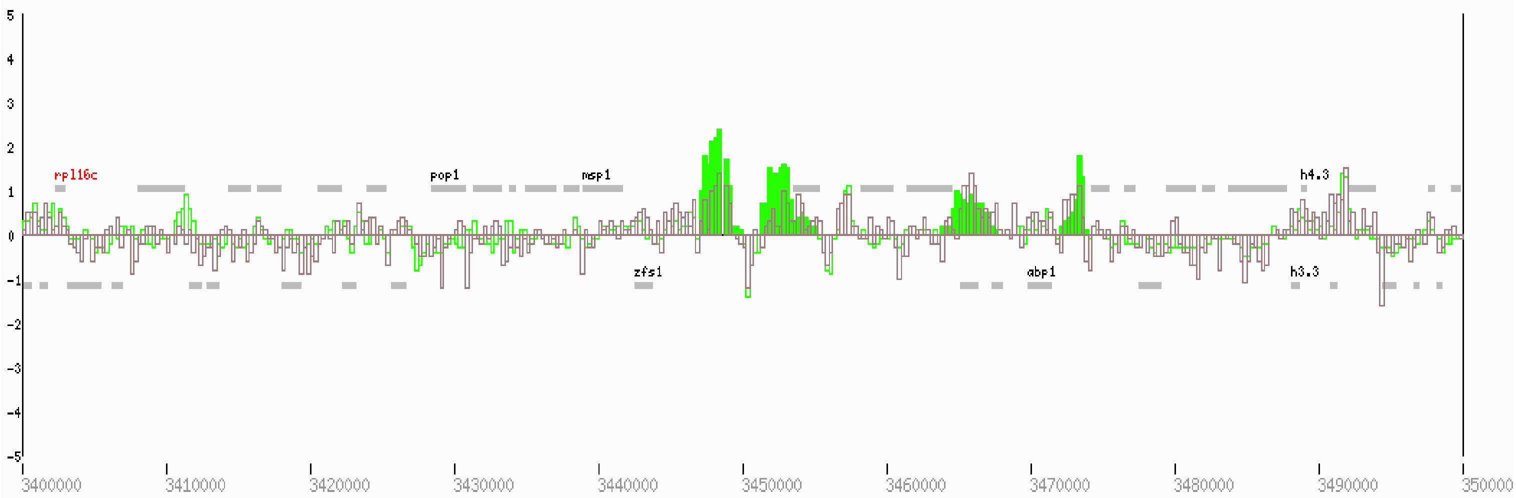


Chromosome 2 (3500000-3600000)

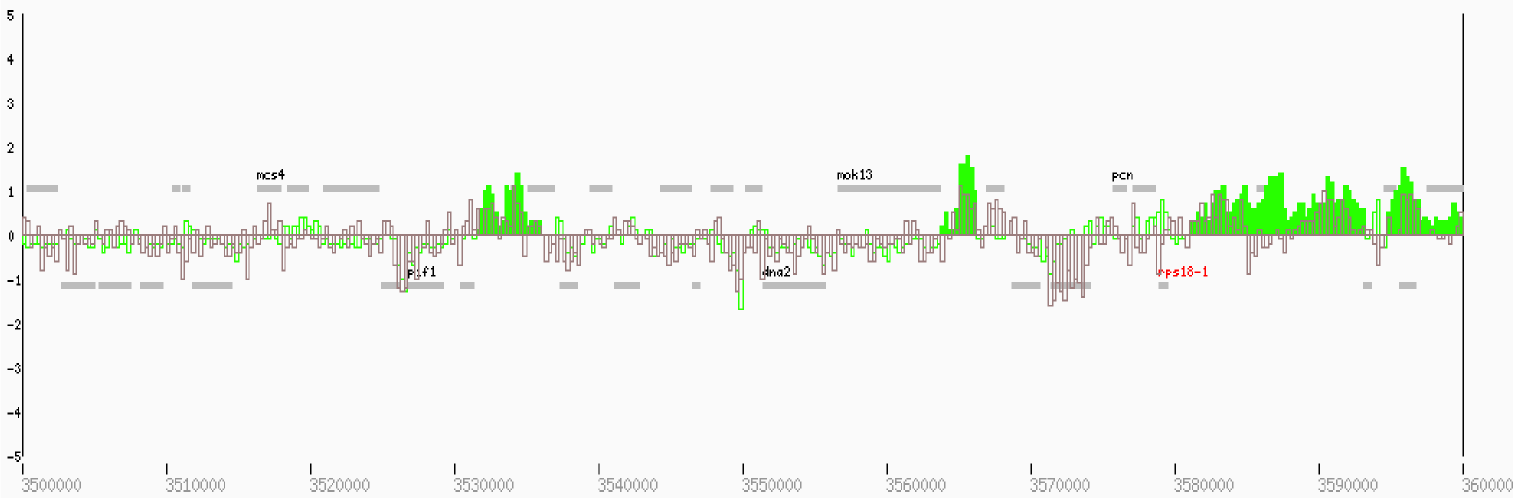

Chromosome 2 (3600000-3700000)

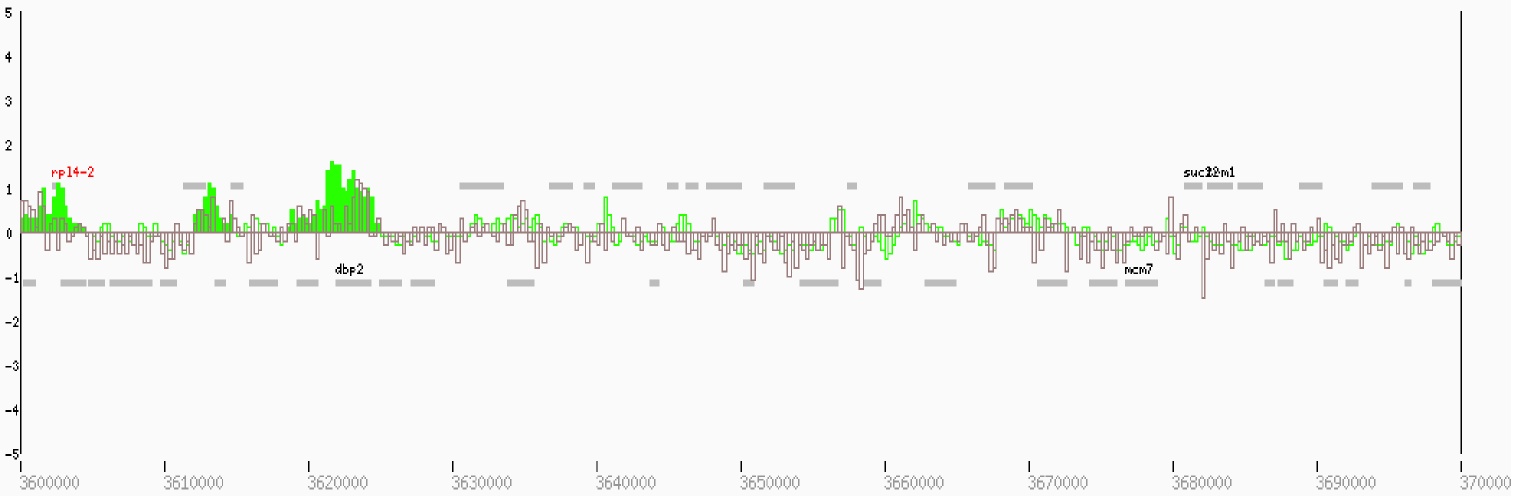


Chromosome 2 (3700000-3800000)

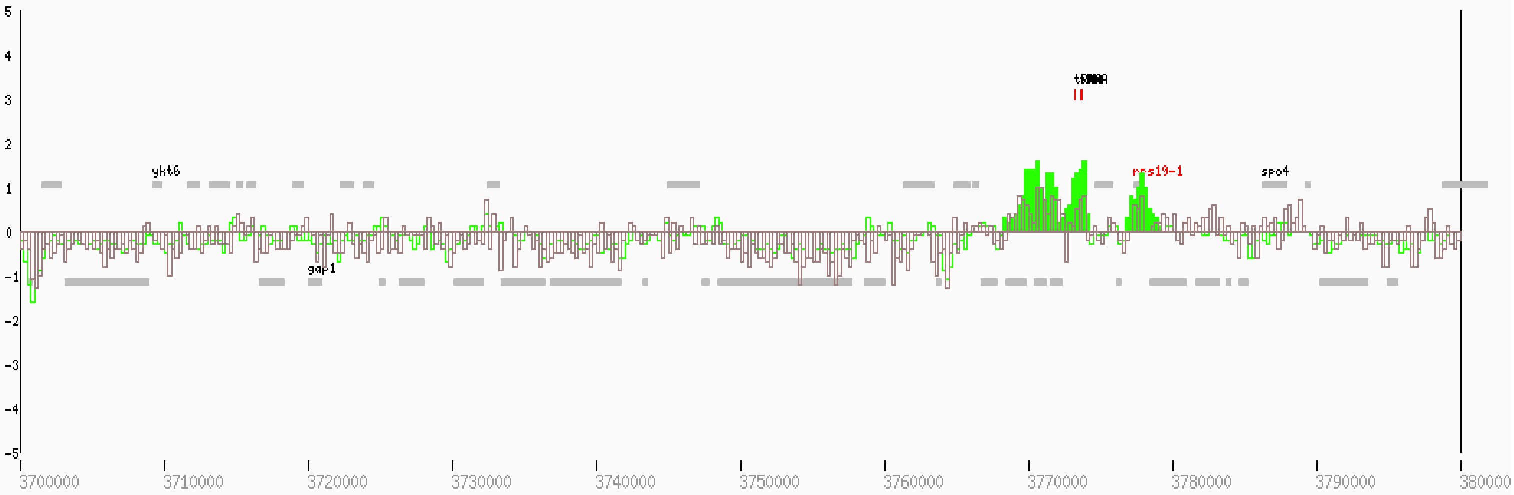


Chromosome 2 (3800000-3900000)

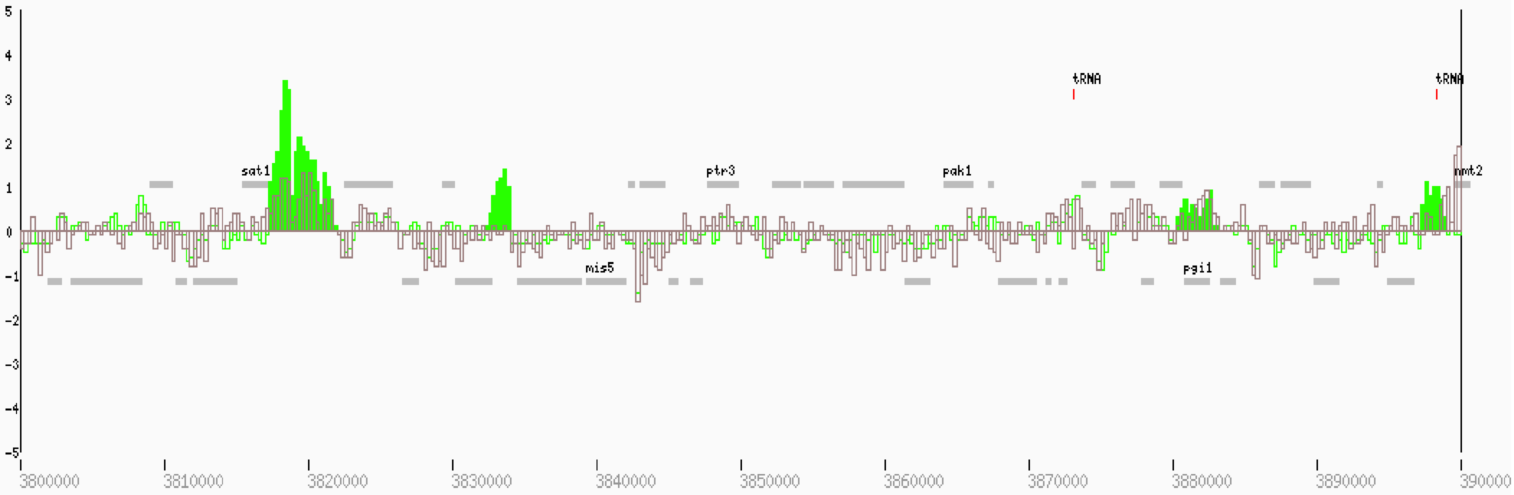


Chromosome 2 (3900000-4000000)

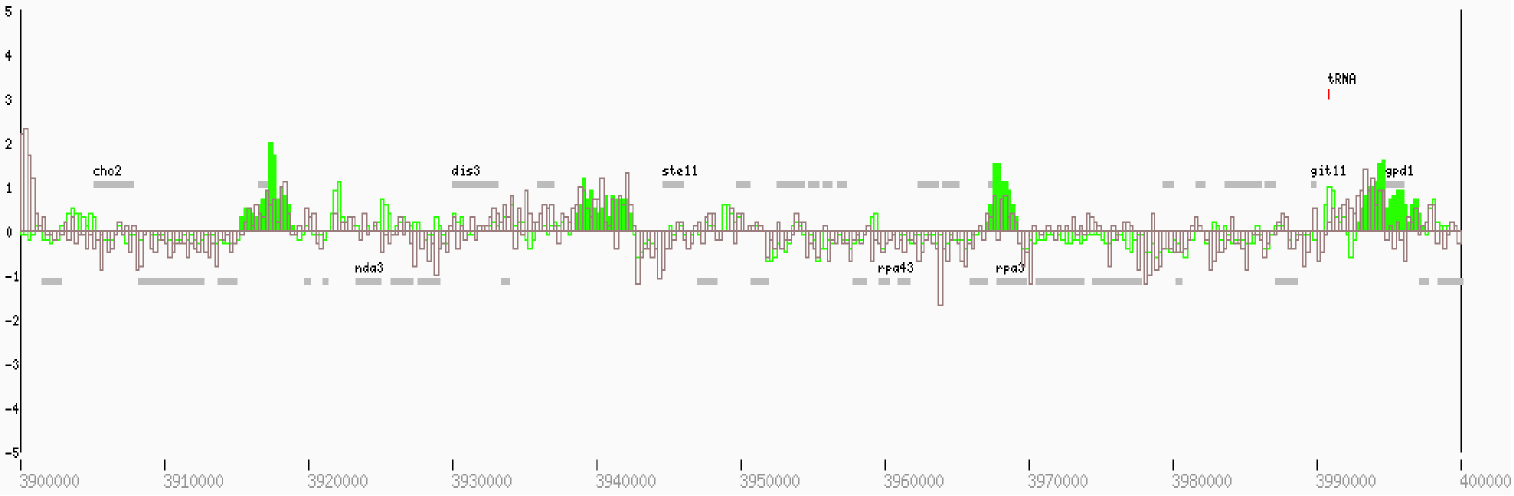

Chromosome 2 (4000000-4100000)

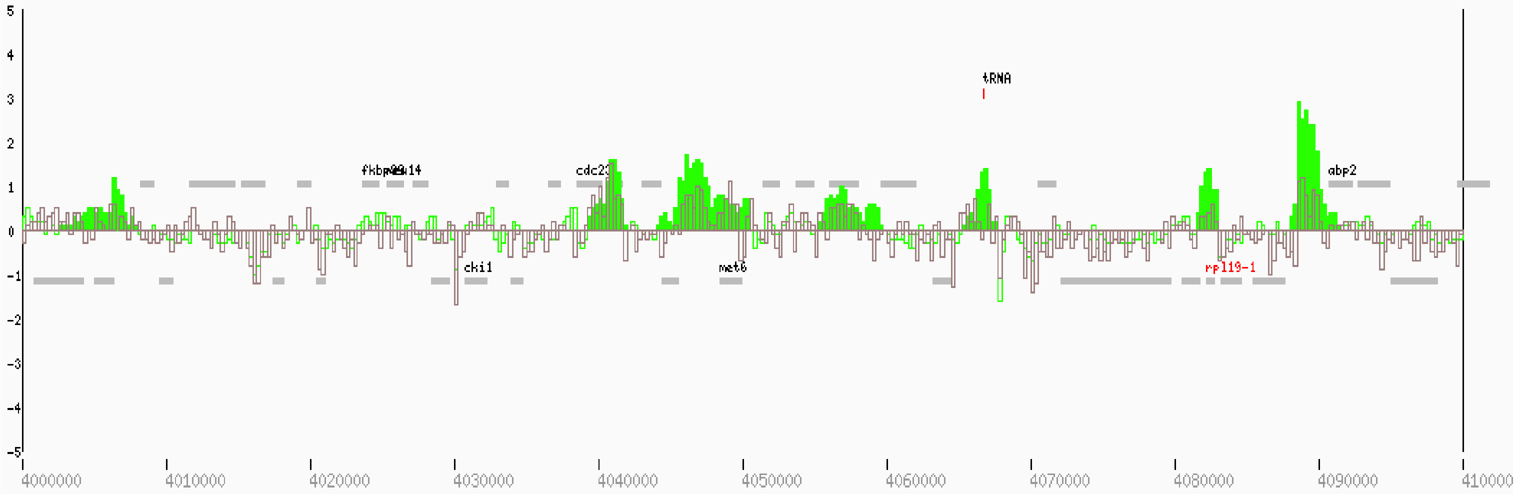


Chromosome 2 (4100000-4200000)

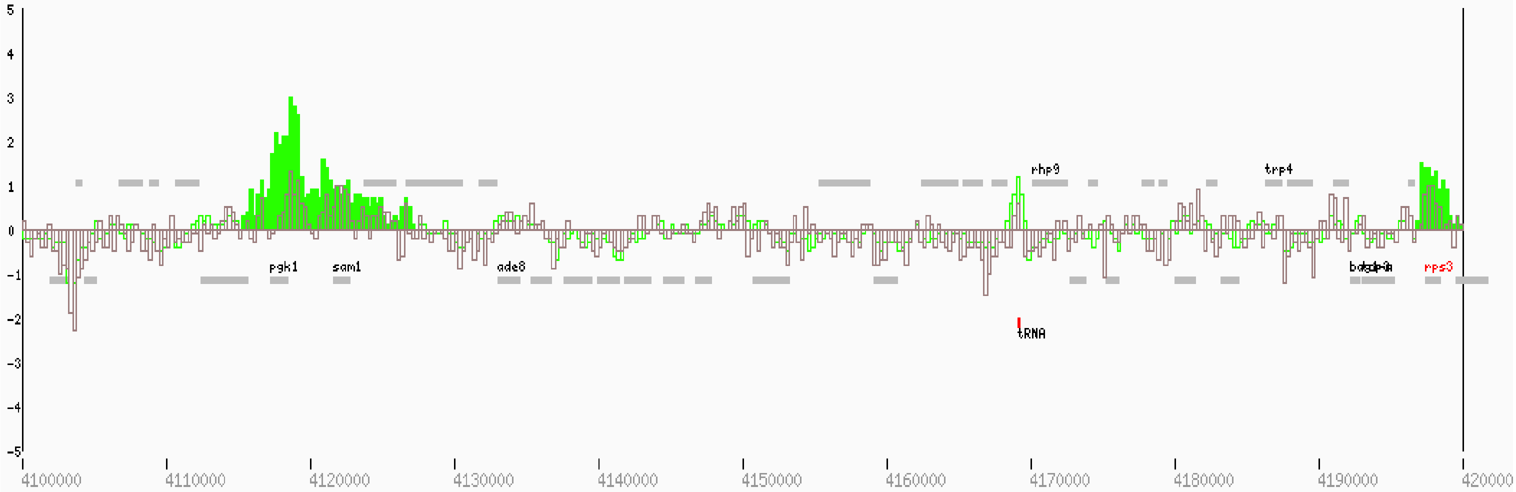


Chromosome 2 (4200000-4300000)

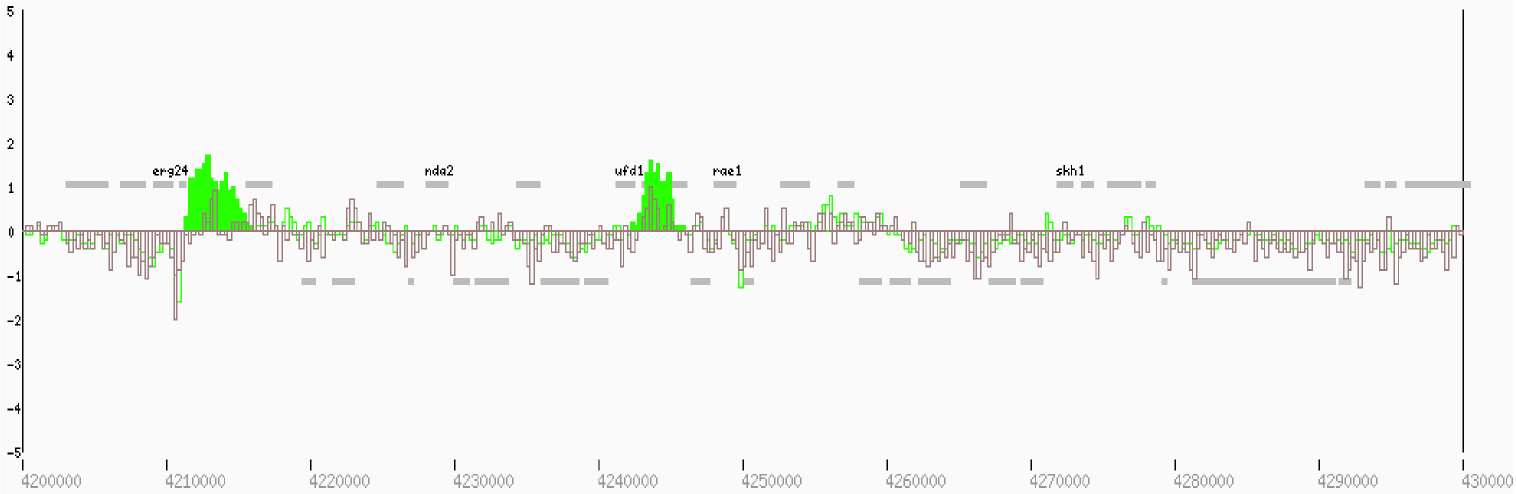


Chromosome 2 (4300000-4400000)

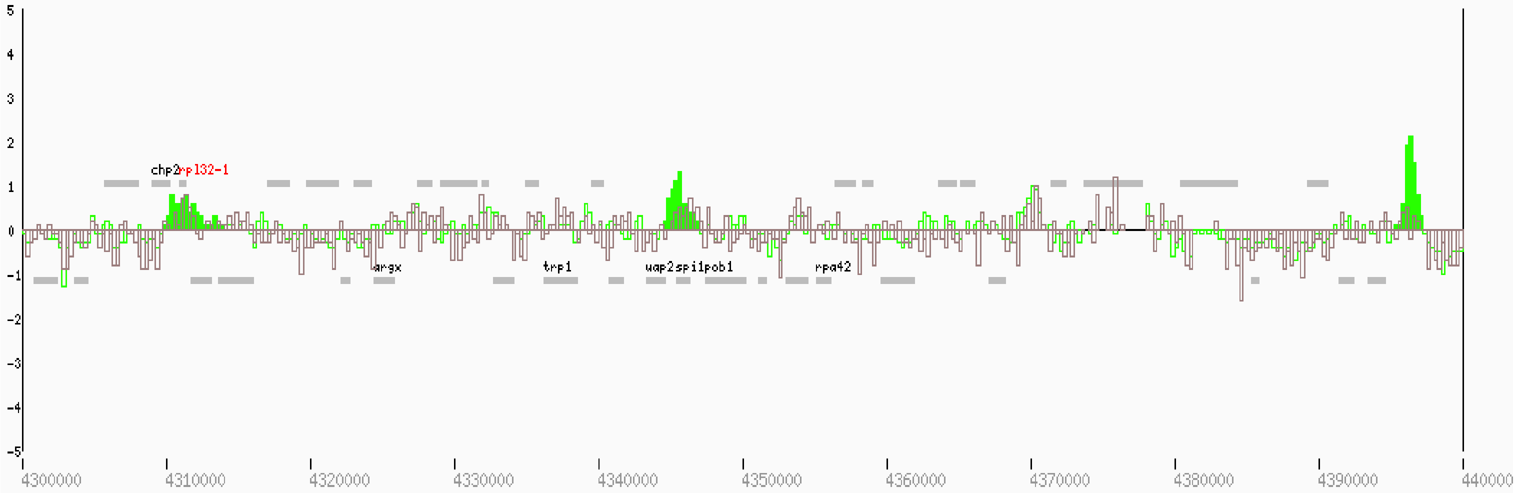

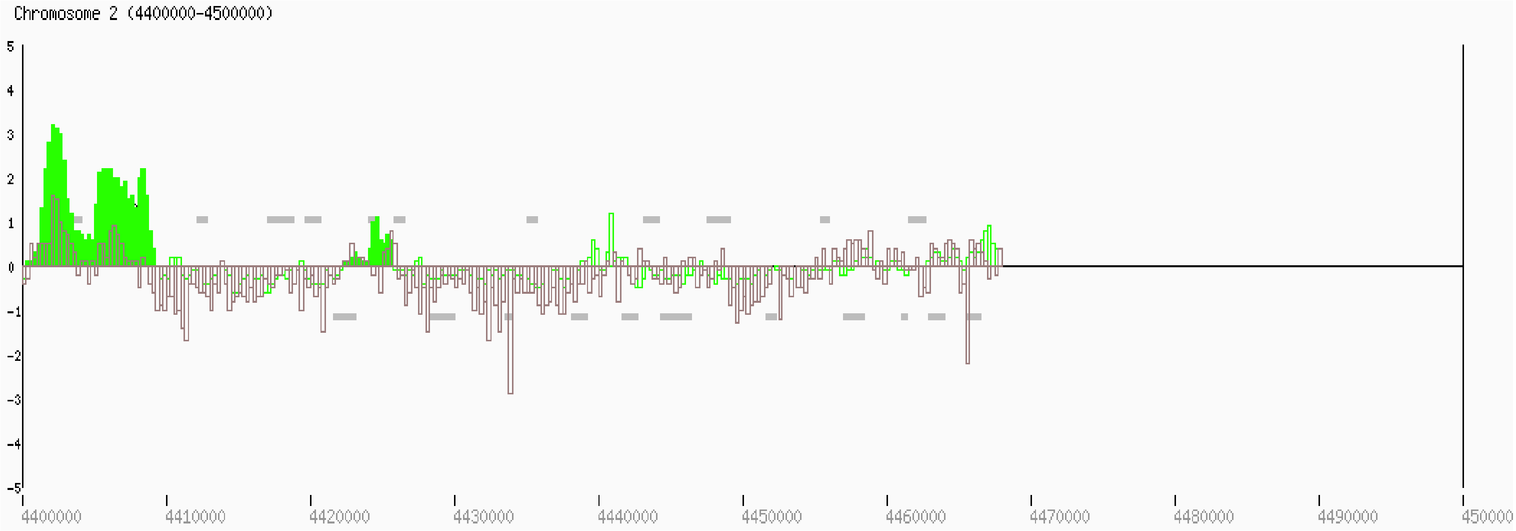

Chromosome 2 (4400000-4500000)
5
4
3
2
1
0
-1
-2
-3
-4
-5
4400000
4410000
4420000
4430000
4440000
4450000
4460000
4470000
4480000
4490000
450000

**Figure S6**

Mis4/Ssl3 binding sites correlate with strongly expressed genes. This graph compares the relative mRNA levels of ribosomal protein genes, Mis4-bound genes other than ribosomal protein genes, and all remaining genes that are not associated with Mis4 along chromosome 2. tRNA genes were excluded from this analysis. Box boundaries mark the $25^{th}$ to $75^{th}$ percentile surrounding the median (bold line). Whiskers extend to 1.5 times the interquartile range. Outliers are indicated as circles. Wilcoxon signed ranks tests suggest that Mis4-bound genes are significantly more strongly expressed than non-bound genes.

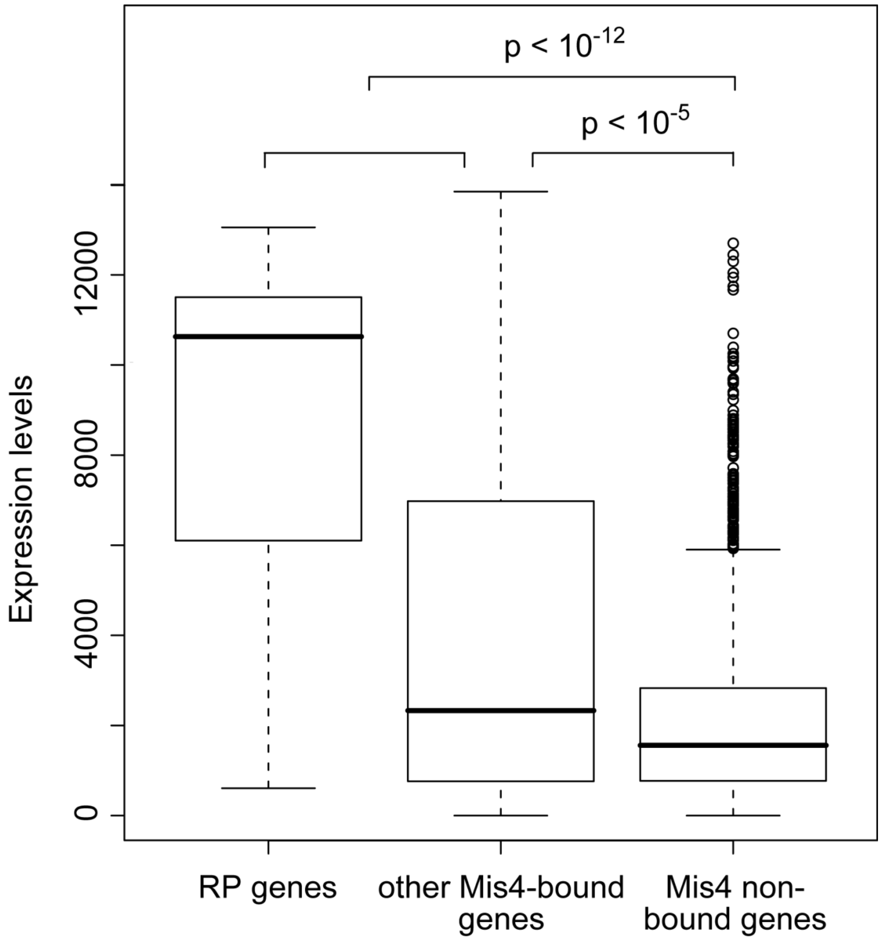

**Figure S7**

The cohesin pattern along chromosome arms remains qualitatively unchanged during cohesin removal in mitosis. Cells were arrested in G2 and synchronously released into mitotic progression. Chromatin immunoprecipitation was performed against the Rad21-$Pk_9$ cohesin subunit. A 100 kb region on the left arm of chromosome 2 in G2-arrested cells is compared to the patterns at the indicated timepoints after release when the majority of cells were in metaphase and in anaphase, respectively (compare Figure 7).

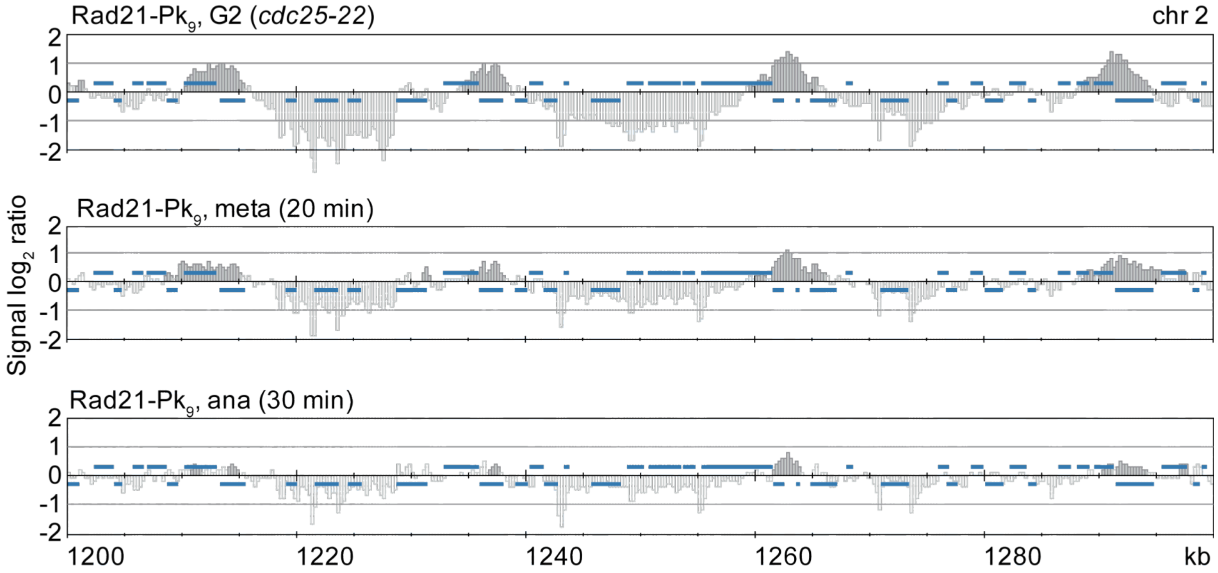

**Table S1**

Yeast strains used in this study.

| Strain | Genotype | Usage |
|---|---|---|
| Y252 | *$h^-$ leu1-32 nda3-KM311 cnd2-$Pk_9$-kanMX6* | Fig. 3 |
| Y2197 | *$h^{90}$ leu1-32 ade6-M216 rad21-$HA_3$-kanMX6* | Fig. 1, A1 |
| Y2447 | *$h^{90}$ leu1-32 psc3-$Pk_9$-kanMX6* | Fig. 1 |
| Y2460 | *$h^{90}$ leu1-32* | Fig. A4 |
| Y2468 | *$h^{90}$ leu1-32 mis4-$Pk_9$-kanMX6* | Fig. 3, 4, 7 A4 |
| Y2699 | *$h^-$ leu1-32 cdc25-22 rad21-$Pk_9$-kanMX6 SV40p-GFP-atb2-LEU2* | Fig. 1, 2, 3, 4, 5, 7, A1, A7 |
| Y2703 | *$h^{90}$ nda3-KM311 rad21-$Pk_9$-kanMX6* | Fig. 5 |
| Y2828 | *$h^-$ pds5-$Pk_9$-kanMX6* | Fig. 1 |
| Y2863 | *$h^-$ leu1-32 cdc10-129 rad21-$Pk_9$-kanMX6* | Fig. 2, 7 |
| Y3071 | *$h^+$ ssl3-$Pk_9$-kanMX6* | Fig. 3, A4 |
| Y3127 | *$h^-$ ura4-D18 rad21-3eGFP-kanMX6 mis4-$Pk_9$-kanMX6* | Fig. 3 |
| Y3169 | *$h^+$ leu1-32 cdc25-22 ssl3-$Pk_9$-kanMX6 SV40p-GFP-atb2-LEU2* | Fig. 6 |
| Y3250 | *$h^{90}$ leu1-32 cdc25-22 cnd2-$Pk_9$-kanMX6 SV40p-GFP-atb2-LEU2* | Fig. 6 |
| Y3272 | *$h^-$ leu1-32 ura4-D18 cdc25-22 rad21-$Pk_9$-kanMX6 SV40p-GFP-atb2-LEU2 swi6::$ura4^+$* | Fig. 2 |
| Y3298 | *$h^{90}$ leu1-32 ura4-D18 his3-D1 lys1 ade6-M210 mis4-$Pk_9$-kanMX6 mis6-GFP-LEU2* | Fig. 6 |
| Y3300 | *$h^{90}$ leu1-32 ura4-D18 rad21-$Pk_9$-kanMX6 mis6-GFP-LEU2* | Fig. 5 |
| Y3303 | *$h^{90}$ leu1-32 his3-D1 cut14-$Pk_9$-kanMX6 mis6-GFP-LEU2* | Fig. 6 |
| Y3350 | *$h^{90}$ leu1-32 mis4-$Pk_9$-kanMX6 fhl1-GFP-hygMX6* | Fig. 3 |
| Y3351 | *$h^{90}$ leu1-32 mis4-$Pk_9$-kanMX6 sfc3-GFP-hygMX6* | Fig. 3 |
| Y3359 | *$h^{90}$ leu1-32 sfc6-$Pk_9$-kanMX6* | Fig. 3 |
| Y3384 | *$h^{90}$ leu1-32 mis4-GFP-hygMX6 cnd2-$Pk_9$-kanMX6* | Fig. 3 |
| Y3385 | *$h^-$ leu1-32 mis4-GFP-hygMX6 ssl3-$Pk_9$-kanMX6* | Fig. 3 |
| Y3403 | *$h^{90}$ leu1-32 fhl1-$Pk_9$-kanMX6* | Fig. 3 |
| Y3586 | *$h^-$ leu1-32 ark1-as3-hygMX6 cdc25-22 rad21-Pk9-kanMX6 SV40p-GFP-atb2-LEU2* | Fig. 7 |
| Y3650 | *$h^-$ leu1-32 ura4-D18 ade6-M210 mis4-$Pk_9$-kanMX6 SPBTRNAGLY.05Δ SPBTRNAARG.04Δ* | Fig. 4 |
| Y3724 | *$h^{90}$ leu1-32 ura4-D18 cdc25-22 rad21-$Pk_9$-kanMX6 SPBTRNAGLY.05Δ SPBTRNAARG.04Δ* | Fig. 4 |
| Y3863 | *$h^{90}$ leu1-32 mis4-$Pk_9$-kanMX6 cdc10-12* | Fig. A5 |

Generation of these strains was based on previously described strains and constructs: *cdc25-22* [54], *nda3-KM311* [55], *cdc10-129* [56], *SV40p-GFP-atb2-LEU2* [57], and *ark1-as3* [41]. A *mis6-GFP-LEU2* strain was a kind gift from Kazu Tomita.
